# Gene expression profiling of long-lived dwarf mice: longevity-associated genes and relationships with diet, gender and aging

**DOI:** 10.1186/1471-2164-8-353

**Published:** 2007-10-03

**Authors:** William R Swindell

**Affiliations:** 1 Department of Pathology, University of Michigan, 3118 BSRB, Ann Arbor, MI, USA

## Abstract

**Background:**

Long-lived strains of dwarf mice carry mutations that suppress growth hormone (GH) and insulin-like growth factor I (IGF-I) signaling. The downstream effects of these endocrine abnormalities, however, are not well understood and it is unclear how these processes interact with aging mechanisms. This study presents a comparative analysis of microarray experiments that have measured hepatic gene expression levels in long-lived strains carrying one of four mutations (*Prop1*^*df*/*df*^, *Pit1*^*dw*/*dw*^, *Ghrhr*^*lit*/*lit*^, *GHR-KO*) and describes how the effects of these mutations relate to one another at the transcriptional level. Points of overlap with the effects of calorie restriction (CR), CR mimetic compounds, low fat diets, gender dimorphism and aging were also examined.

**Results:**

All dwarf mutations had larger and more consistent effects on IGF-I expression than dietary treatments. In comparison to dwarf mutations, however, the transcriptional effects of CR (and some CR mimetics) overlapped more strongly with those of aging. Surprisingly, the *Ghrhr*^*lit*/*lit *^mutation had much larger effects on gene expression than the *GHR-KO *mutation, even though both mutations affect the same endocrine pathway. Several genes potentially regulated or co-regulated with the IGF-I transcript in liver tissue were identified, including a DNA repair gene (Snm1) that is upregulated in proportion to IGF-I inhibition. A total of 13 genes exhibiting parallel differential expression patterns among all four strains of long-lived dwarf mice were identified, in addition to 30 genes with matching differential expression patterns in multiple long-lived dwarf strains and under CR.

**Conclusion:**

Comparative analysis of microarray datasets can identify patterns and consistencies not discernable from any one dataset individually. This study implements new analytical approaches to provide a detailed comparison among the effects of life-extending mutations, dietary treatments, gender and aging. This comparison provides insight into a broad range of issues relevant to the study of mammalian aging. In this context, 43 longevity-associated genes are identified and individual genes with the highest level of support among all microarray experiments are highlighted. These results provide promising targets for future experimental investigation as well as potential clues for understanding the functional basis of lifespan extension in mammalian systems.

## Background

Long-lived dwarf strains of laboratory mice offer promising tools for advancing our understanding of aging mechanisms and the basis of extended lifespan in mammals. Dwarf models have been found to exhibit an average lifespan increase of more than 50% compared to wildtype control strains, and thus represent genetic manipulations with an impact on longevity that is comparable to calorie restricted diets [1]. In addition to lifespan extension, long-lived dwarf strains exhibit superior health at advanced ages [2], and are less susceptible to age-related declines in memory, learning ability, and locomotion [3]. Dwarf mice experience lower incidence of kidney disease, cataracts and joint disease, as well as fatal neoplastic disease, such as lymphoma and adenocarcinoma [4-6]. Earlier studies have also suggested that development of transplanted tumors is impeded in dwarf mice, and cancer rates are reduced following exposure to chemical carcinogens [7,8]. The loss of immune function and progression of collagen cross-linking that normally occurs with advancing age is diminished in some dwarf strains [9], and at the cellular level, fibroblasts of dwarf mice are resistant to several forms of stress, including oxidative stress, ultraviolet light, toxic metals and heat [10,11]. Aside from improving our knowledge of aging mechanisms, therefore, understanding the unique features of dwarf mice may provide insight into a broad range of mechanisms relevant to health and disease-prevention in mammals.

Long-lived dwarf mouse strains carry mutations that suppress the growth hormone (GH)/insulin-like growth factor I (IGF-I) endocrine pathway [1]. In Ames (*Prop-1*^*df*/*df*^) and Snell (*Pit-1*^*dw*/*dw*^) mutants, for instance, development of the anterior pituitary is inhibited completely. This results in reduced GH and IGF-I levels, as well as other hormonal abnormalities, such as deficiency of thyroid stimulating hormone and prolactin [12,13]. Ames and Snell dwarfs exhibit considerably reduced body size, but also have dramatic lifespan extension of 40–69% on average. In the Little (*Ghrhr*^*lit*/*lit*^) mouse, lifespan extension is less substantial (23–25%), and only occurs when mice are provided a low fat diet [9]. Hormonal abnormality of Little mice is limited to circulating GH levels, which are reduced because the *lit/lit *mutation renders the pituitary unresponsive to GH releasing hormone [14]. It is somewhat surprising that genetic alterations downstream of the *lit/lit *mutation promote a larger lifespan increase than that observed in little mice. In particular, GH-receptor knock-out mice (GHR-KO) have elevated serum GH levels, but are growth hormone resistant and exhibit considerable lifespan increases of 38–55% on average [15]. Taken together, these findings from four long-lived dwarf models firmly establish an endocrine basis of lifespan extension in mammals. Nevertheless, it remains unclear why such endocrine abnormalities affect lifespan, since inhibition of GH/IGF-I signaling is associated with extensive downstream effects, and only a fraction of these effects may be linked to mechanisms of aging [16].

Identification of genes affected by GH and IGF-I suppression is an important step towards understanding how this axis impacts longevity. Microarray studies of long-lived dwarf mice have been especially useful in this regard [17-20]. Previous studies have focused primarily on liver tissue, since this is the major manufacturing site of IGF-I. Early investigations used cDNA arrays to detect expression differences between dwarf and normal mice, and found that 13 of 265 surveyed transcripts (4.9%) were differentially expressed in Ames dwarfs [17], while 60 of 2352 (2.5%) surveyed transcripts were differentially expressed in the Snell model [18]. Subsequent studies have used Affymetrix oligonucleotide arrays to screen a larger fraction of the genome. For instance, Amador-Noguez et al. [19] found that approximately 1100 of 14-thousand Affymetrix probesets (8.1%) were differentially expressed in Ames and Little dwarfs, respectively, while 547 (4.0%) transcripts were differentially expressed in both models. Boyleston et al. [20] performed a similar study using Ames and Snell dwarf mice, but focused only on probesets differentially expressed at every age group examined (6 – 24 months). This approach highlighted 785 such probesets in the Ames dwarf (1.9% of those surveyed), along with 205 probesets (1.7%) in the Snell dwarf, with 49 probesets satisfying the criterion in both long-lived models.

Previous microarray studies of long-lived dwarf mice have used varying statistical methodologies and criteria for identifying candidate genes, which complicates comparisons among studies based upon published results. Such comparisons would be useful, since genes differentially expressed with respect to multiple long-lived dwarf models are especially likely to play a role in aging and lifespan determination [17,18]. Endocrine abnormalities differ to some degree among dwarf models, but presumably, lifespan extension in each model results from shared alterations that affect the GH/IGF-I pathway. Genes underlying extended lifespan should therefore be identified with respect to all long-lived models, while genes involved in pathways unrelated to lifespan extension should be specific to particular models. Furthermore, from a statistical perspective, a fraction of genes identified with respect to any one long-lived model in a single study are expected to be false-positives [21]. However, since P-values combine multiplicatively when results are pooled across studies [22], genes differentially expressed in more than one study are less likely to be false positive identifications. From biological and statistical standpoints, therefore, it is desirable to utilize data from independent studies to identify genes commonly induced among multiple long-lived dwarf models.

This study presents a comparative analysis of microarray experiments that have examined hepatic gene expression differences between long-lived dwarf mice and normal controls. The results provide a detailed view of how the effects of different dwarf mutations relate to one another in terms of gene expression, and describe how these effects relate to those associated with aging, gender dimorphism, low fat diets, caloric restriction (CR) and several different CR mimetic compounds. A main goal of the analysis was to identify longevity-associated genes with the highest level of support based on currently available microarray data. Applying consistent statistical methodology, therefore, genes exhibiting parallel differential expression patterns in four long-lived dwarf models were identified (Ames, Snell, Little and GHR-KO). Since mechanisms of lifespan extension in long-lived dwarf models may overlap with those conferring increased longevity under CR [23-25], genes exhibiting parallel transcriptional changes in multiple long-lived models and under CR were also identified. Further steps were taken to evaluate the role of each candidate gene with respect to aging and longevity, and to determine which genes provided the most promise for future experimental investigation.

## Results

### Datasets and IGF-I expression patterns

Expression data was examined from four dwarf models that have previously been associated with significant lifespan extension in laboratory studies, including the Ames (*Prop1*^*df*/*df*^), Snell (*Pit1*^*dw*/*dw*^), Little (*Ghrhr*^*lit*/*lit*^), and GHR-KO (*GHR-KO*) mutants. Genetic alterations with effects potentially related to those of life-extending dwarf mutations were also considered. For instance, the effects of two GHR knock-in models with disruptions of GHR receptor residues 391 or 569 were examined [26]. Gene expression levels of the B6.C3H-6T congenic mouse were considered because this model exhibits a 30–40% reduction in serum IGF-I levels [27]. The effects of gender were examined because female mice live longer than male mice, and also because one study has suggested that dwarf mutations induce a masculine-to-feminine shift in gene expression patterns [20]. Dietary treatments known or postulated to increase lifespan were considered for the purpose of comparison. These treatments included low fat diets, CR and several potential CR mimetic compounds (metformin, glipizide, rosiglitazone, and soy isoflavone extract). The potential CR mimetic compounds are known to either influence insulin sensitivity (metformin, glipizide, rosiglitazone) or suppress tumorigenesis (soy isoflavone extract) [28]. Metformin treatment of female mice has been found to increase mean and maximum lifespan by 8% and 13%, respectively [29].

The analysis is based on a series of *contrasts *comparing gene expression levels between two experimental treatments. Table 1 provides descriptions and representative symbols for all contrasts examined. Each contrast involved a pair of treatments designated *A *and *B*. The *A *treatment is a genotype or dietary manipulation known or postulated to be associated with increased longevity. The corresponding *B *treatment is an appropriate control that permitted evaluation of how treatment *A *affects gene expression. The analysis was based on 8525 probesets that could be reliably matched among all three platforms (using Affymetrix best match tables). For each contrast, the null hypothesis of *H*_0_: *μ*_*Ai *_= *μ*_*Bi *_was tested for each probeset, where *μ*_*Ai *_and *μ*_*Bi *_represent the mean expression levels of gene *i *in treatments *A *and *B*, respectively (see Methods).

**Table 1:** Treatment Contrasts

Contrast Symbol	Treatment *A*	Treatment *B*
snell5^a^	*Pit1*^*dw*/*dw *^males, age 4–6 months, *n *= 4	*Pit1*^*dw*/*? *^control males, age 4–6 months, *n *= 4
snell25^a^	*Pit1*^*dw*/*dw *^males, age 24–26 months, *n *= 3	*Pit1*^*dw*/*? *^control males, age 24–26 months, *n *= 3
ames5A^b^	*Prop1*^*df*/*df *^males, age 4–6 months, *n *= 5	*Prop1*^+/+ ^control males, age 4–6 months, *n *= 5
ames13A^b^	*Prop1*^*df*/*df *^males, age 12–14 months, *n *= 5	*Prop1*^+/+ ^control males, age 12–14 months, *n *= 5
ames25A^b^	*Prop1*^*df*/*df *^males, age 24–27 months, *n *= 5	*Prop1*^+/+ ^control males, age 24–27 months, *n *= 5
ames3B^c^	*Prop1*^*df*/*df *^males, age 3 months, *n *= 3	*Prop1*^+/+ ^control males, age 3 months, *n *= 5
ames6B^c^	*Prop1*^*df*/*df *^males, age 6 months, *n *= 3	*Prop1*^+/+ ^control males, age 6 months, *n *= 5
ames12B^c^	*Prop1*^*df*/*df *^males, age 12 months, *n *= 3	*Prop1*^+/+ ^control males, age 12 months, *n *= 5
ames24B^c^	*Prop1*^*df*/*df *^males, age 24 months, *n *= 3	*Prop1*^+/+ ^control males, age 24 months, *n *= 5
little3^c^	*Ghrhr*^*lit*/*lit *^males, age 3 months, *n *= 3	*Ghrhr*^*lit*/+ ^males, age 3 months, *n *= 3
little6^c^	*Ghrhr*^*lit*/*lit *^males, age 6 months, *n *= 3	*Ghrhr*^*lit*/+ ^males, age 6 months, *n *= 3
little12^c^	*Ghrhr*^*lit*/*lit *^males, age 12 months, *n *= 3	*Ghrhr*^*lit*/+ ^males, age 12 months, *n *= 3
little24^c^	*Ghrhr*^*lit*/*lit *^males, age 24 months, *n *= 3	*Ghrhr*^*lit*/+ ^males, age 24 months, *n *= 3
GHR-KO^d^	GHR(-/-) males, age 42 days, *n *= 3	wild type males, age 42 days, *n *= 3
GHR-KI1^d^	GHR knock-in mutant 569, males, age 42 days, *n *= 3	wild type males, age 42 days, *n *= 3
GHR-KI2^d^	GHR knock-in mutant 391, males, age 42 days, *n *= 3	wild type males, age 42 days, *n *= 3
B6^e^	C57BL/6J (B6) females, age 2 months, *n *= 3 (20–30% reduced serum IGF-I)	C3H/HeJ (C3H) females, age 2 months, *n *= 3
Gender^f^	wild type females, age 3–6 months, *n *= 6	wild type males, age 3–6 months, *n *= 6
cr(2,6)^g^	4 months of 30% calorie restriction, initiated age 2, tissue harvest age 6, wild type females, *n *= 8	wild type littermates of treatment *A *fed on control diet, *n *= 7
cr(2,6)df^g^	4 months of 30% calorie restriction, initiated age 2, tissue harvest age 6, *Prop1*^*df*/*df *^females, *n *= 8	*Prop1*^*df*/*df *^female littermates of treatment *A *with control diet, *n *= 8
cr(20,22)^h^	2 months of 40% calorie restriction, initiated age 20, tissue harvest age 22, strain B6C3F1 males, *n *= 4	strain B6C3F1 males fed on control diet, tissue harvest age 22, *n *= 4
cr(5,22)^h^	17 months 40% calorie restriction, initiated age 5, tissue harvest age 22, strain B6C3F1 males, *n *= 4	strain B6C3F1 males fed on control diet, tissue harvest age 22, *n *= 4
met^h^	metformin, 2100 mg per kg diet, tissue harvest age 22, strain B6C3F1 males, *n *= 4	control diet, tissue harvest age 22, strain B6C3F1 males, *n *= 4
met(db/db)^i^	metformin, 400 mg/kg, C57BL/ksj – *db/db *males, *n *= 5	placebo, C57BL/ksj – *db/db *males, *n *= 5
glip^h^	glipizide, 1050 mg per kg diet, tissue harvest age 22, strain B6C3F1 males, *n *= 4	control diet, tissue harvest age 22, strain B6C3F1 males, *n *= 4
gm^h^	metformin & glipizide, met dose: 1050 mg per kg diet, glip dose: 525 mg per kg diet, tissue harvest age 22, strain B6C3F1 males, *n *= 4	control diet, tissue harvest age 22, strain B6C3F1 males, *n *= 4
ros^h^	rosiglitazone, 80 mg per kg diet, tissue harvest age 22, strain B6C3F1 males, *n *= 4	control diet, tissue harvest age 22, strain B6C3F1 males, *n *= 4
soy^h^	soy isoflavone extract, 0.25%, tissue harvest age 22, strain B6C3F1 males, *n *= 4	control diet, tissue harvest age 22, strain B6C3F1 males, *n *= 4
lowfat1^j^	4.5% fat diet by weight, congenic C57BL/6J males, *n *= 3	21% fat diet by weight, congenic C57BL/6J males, *n *= 3
lowfat2^k^	very low fat diet, strain C57BL/6J males, *n *= 5	normal diet, strain C57BL/6J males, *n *= 5
age^l^	age 4 months, wild type, *n *= 4	age 32 months, wild type, *n *= 4

Each contrast corresponds to a test of differential expression and set of genes identified as significantly upregulated or downregulated. The *A *column lists treatments known or hypothesized to be associated with increased lifespan. The *B *column lists comparable control treatments associated with normal lifespan. All contrasts were of the form *A *– *B*, such that upregulated genes exhibit increased expression in treatment *A *(relative to treatment *B*) while downregulated genes exhibit decreased expression in treatment *A *(relative to treatment *B*). The RNA source for all treatments was liver and the value *n *refers to the number of independent biological replicates available for each treatment.

^a^GSE3129, MG-U74Av2, Boyleston et al. [20]

^b^GSE3150, MG 430 2.0, Boyleston et al. [20]

^c^EMEXP153, MOE 430A, Amador-Noguez et al. [19]

^d^GSE988, MG-U74Av2, Rowland et al. [26]

^e^GSE5959, MG-U74Av2, Adamo et al. [27]

^f^EMEXP347, MOE 430A, Amador-Noguez et al. [87]

^g^GSE1093, MG-U74Av2, Tsuchiya et al. [24]

^h^GSE2431, MG-U74Av2, Dhahbi et al. [28]

^i^EMEXP490, MG-U74Av2, Heishi et al. [88]

^j^GSE363, MG-U74Av2, Recinos et al. [89]

^k^GSE3889, MG 430 2.0, Flowers et al. [90]

^l^EMEXP839, MG 430 2.0, Niedernhofer et al. [91]

**Figure 1:**
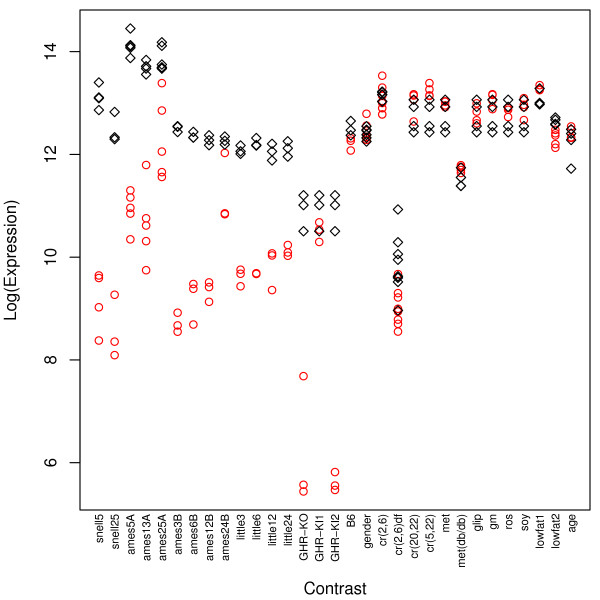
**IGF-I expression**. The vertical axis corresponds to log-transformed gene expression values. The 31 contrasts listed in Table 1 are ordered along the horizontal axis. Red circles correspond to expression values of replicate observations associated with the *A *treatment of each contrast, while black diamonds indicate replicate expression values associated with the control *B *treatment of each contrast (see Table 1).

The Snell, Ames and Little dwarf mutants were associated with large affects on gene expression at every age examined. Of 8525 probesets, the total number of genes differentially expressed with respect to these dwarf mutants ranged from 151 (contrast snell25) to as many as 987 (contrast ames3b) (see Table 2). IGF-I expression was significantly downregulated in all contrasts involving the Snell, Ames and Little dwarfs (Fig. 1, Table 2). IGF-I suppression, however, may account for only a small fraction of genes differentially expressed in these models. This is because GHR-KO mice exhibited significant IGF-I transcript downregulation of larger magnitude than that observed in the Snell, Ames and Little models, but nevertheless, only 46 genes exhibited differential expression in GHR-KO mice (see Table 2). This suggests that a relatively small number of genes may be specifically affected by IGF-I suppression in mouse liver. It is interesting to note, moreover, that IGF-I transcript levels are significantly downregulated with respect to the GHR-KI2 contrast, but not with respect to the GHR-KI1 contrast (Fig. 1, Table 2). This difference is most likely attributable to the STAT5 signaling pathway (signal transducer and activator of transcriptions 5) downstream of GHR. GHR-KI1 mice lack GHR residue 569, but exhibit only slight loss of hepatic IGF-1 transcript because STAT5 signaling is partially functional [26]. GHR-KI2 mice, however, lack GHR residue 391 and have no STAT5 signaling capacity, resulting in substantial loss of IGF-1 transcript in liver [26].

**Table 2:** Overview of Differential Expression Results

Contrast Symbol	IGF1 [log_2_(FC)]	Upregulated	Downregulated
snell5	-3.96*	129	187
snell25	-3.91*	65	86
ames4A	-3.20*	347	273
ames10A	-3.05*	314	214
ames22A	-1.55*	229	171
ames3B	-3.79*	567	420
ames6B	-3.18*	501	470
ames12B	-2.92*	424	307
ames24B	-1.03*	305	193
little3	-2.46*	359	291
little6	-2.54*	328	314
little12	-2.23*	200	310
little24	-1.99*	206	269
GHR-KO	-4.68*	8	38
GHR-KI1	-0.40	5	5
GHR-KI2	-5.29*	24	13
B6	-0.28	1	7
Gender	0.05	243	284
cr(2, 6)	-0.08	93	67
cr(2, 6)df	-0.85*	22	24
cr(20, 22)	0.26	65	116
cr(5, 22)	0.49	279	529
met	0.24	127	172
met (db/db)	0.20	166	523
glip	0.03	80	49
gm	0.32	114	107
ros	0.11	51	65
soy	0.17	45	32
lowfat1	0.20	6	8
lowfat2	-0.31	96	54
age	0.20	494	458

For each contrast, the number of probesets significantly upregulated and downregulated at a significance level of 0.05 is listed. The log-transformed fold-change [log_2_(FC)] associated with the insulin-like growth factor I (IGF1) transcript is also listed.

*test for differential expression significant with P < 0.05

**Figure 2:**
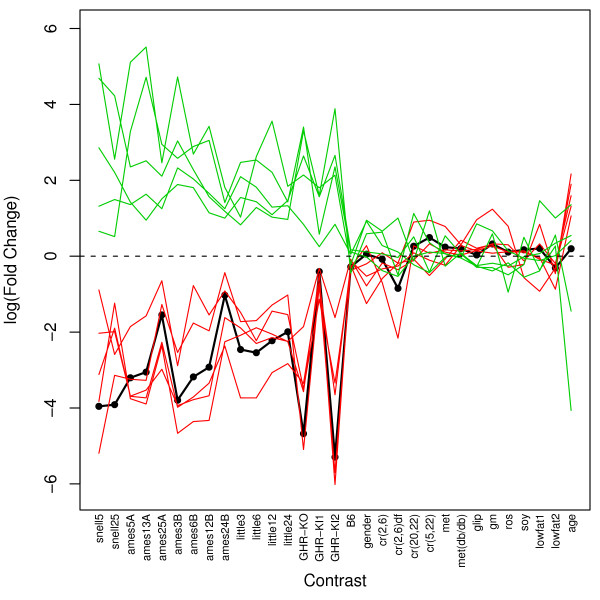
**Potential IGF-I regulated or co-regulated genes**. The vertical axis corresponds to log-transformed fold-change. The 31 contrasts listed in Table 1 are ordered along the horizontal axis. The thick black line represents fold-changes associated with IGF-I across the 31 contrasts. Red lines represent five genes for which fold-changes across contrasts are most positively associated with those of IGF-I (Mup3, Es31, Igfals, Keg1, Socs2). Green lines represent five genes for which fold-changes across contrasts are most negatively associated with those of IGF-I (Scd2, Slc16a7, Pcp4l1, Snm1, Igfbp1). Additional files 1 and 2 provide plots for the top 40 genes most positively and negatively associated with the IGF-I expression pattern among all contrasts.

IGF-I expression was far more sensitive to the dwarf mutations than any of the dietary treatments examined, including CR (see Fig. 1). IGF-I was significantly downregulated in all contrasts involving dwarf mutants, but only one of four CR treatments resulted in significant IGF-I downregulation [contrast cr(2,6)df]. For all other dietary treatments, such as low fat diets and CR mimetics, IGF-I transcript levels were not significantly affected, and in fact, were slightly upregulated in most cases (Fig. 1, Table 2). Several genes exhibited expression patterns among all contrasts that were positively or negatively related to IGF-I expression (Fig. 2 and Additional files 1 and 2). These genes are potentially regulated by or coregulated with the IGF-I transcript in mouse liver tissue.

### Differential expression signatures

The *differential expression signature *associated with a given contrast was determined by testing *H*_0_: *μ*_*Ai *_= *μ*_*Bi *_for all *i *= 1,..., 8525 probesets included in the analysis. Genes for which *H*_0_: *μ*_*Ai *_= *μ*_*Bi *_was not rejected were assigned a score of 0. Genes for which *H*_0_: *μ*_*Ai *_= *μ*_*Bi *_was rejected were assigned a score of ± 1, depending on whether the gene was upregulated (mean expression higher in treatment *A *relative to *B*) or downregulated (mean expression lower in treatment *A *relative to *B*). The pattern of 0, 1, and -1 scores among all *i *= 1, .., 8525 genes defined the differential expression signature of each contrast.

**Figure 3:**
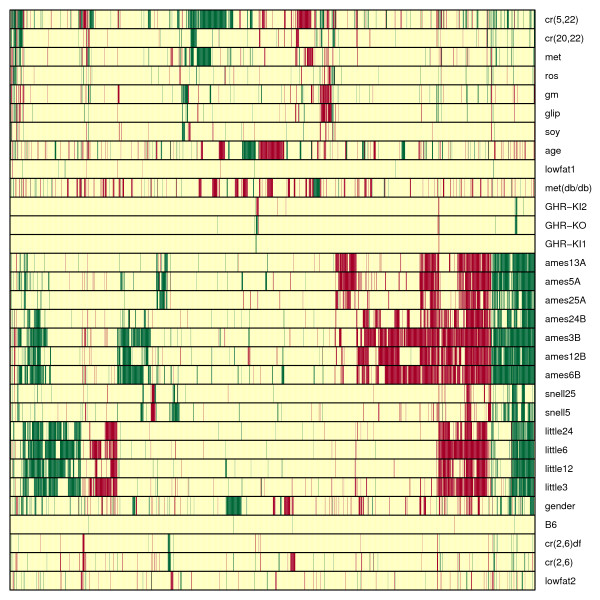
**Differential Expression Signatures**. Each row corresponds to one of the contrasts listed in Table 1. Each column corresponds to one of 2192 genes differentially expressed with respect to more than one contrast. Rows have been ordered to correspond to the dendrogram shown in Figure 4. Red coloring indicates that a gene is upregulated (P < 0.05), while green coloring indicates that a gene is downregulated (P < 0.05).

**Figure 4:**
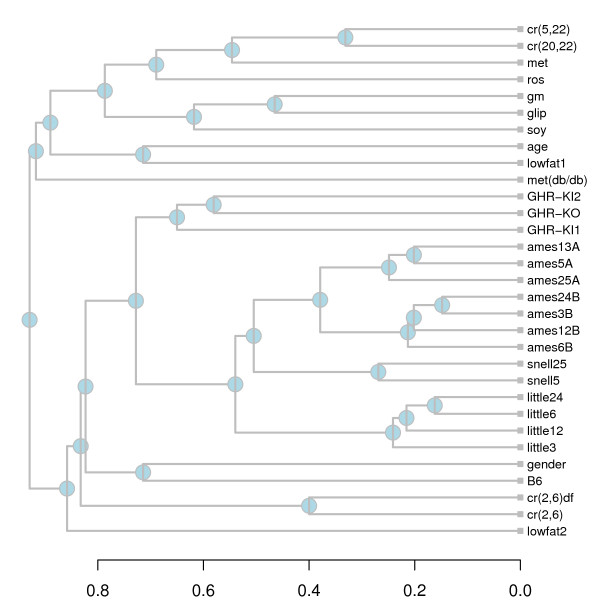
**Hierarchical cluster analysis**. Each branch corresponds to a differential expression signature shown in Figure 3. The horizontal axis indicates the average level of similarity at which two clusters were joined (see Equation 1 in Methods).

Three of the four dwarf mutants (Snell, Ames, Little) were associated with similar differential expression signatures (Fig. 3). While the GHR-KO mutation was associated with only a small number of transcriptional changes, most of these changes were also found in the other three long-lived models (Fig. 3). This similarity among long-lived models is reflected by a hierarchical cluster analysis of differential expression signatures (see Methods). All signatures associated with long-lived dwarf models clustered together in a single branch, and sub-branches within this single branch are joined at high levels of similarity (see Fig. 4). Dietary treatments, on the other hand, clustered together at lower levels of similarity and in some cases were placed in separate branches. It is noteworthy, for instance, that signatures associated with contrasts cr(2,6)df and cr(2,6) clustered in a branch apart from the signatures of cr(5,22) and cr(20,22). This reflects the degree to which the affect of CR on gene expression depends on how CR was carried out and, more particularly, on the age at which necropsies are performed.

**Figure 5:**
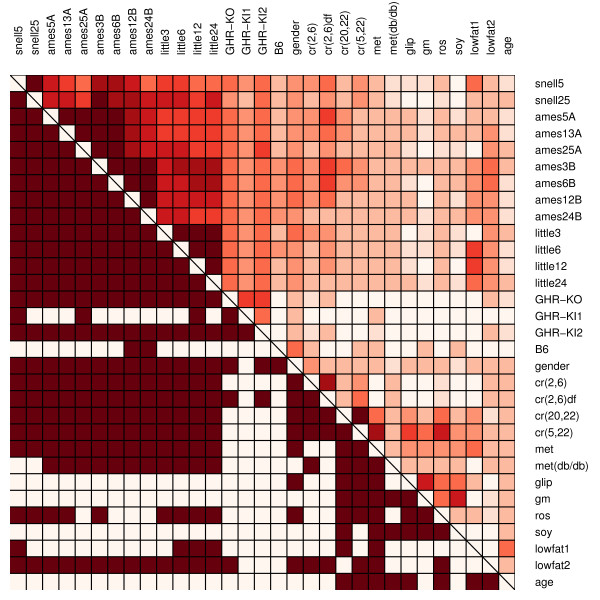
**Differential expression signature similarity matrix**. In the upper-right triangle region, dark red colors indicate high similarity between signatures associated with two contrasts (indicated by row and column labels). This similarity is defined by Equation (1) in the Methods section. The binary coding in the lower-left triangle region indicates whether signatures associated with two contrasts exhibit a significant level of similarity. The statistical procedure used to evaluate similarity is described in the Methods section. Contrast pairs with significant similarity (P < 0.05) are coded dark red, while pairs with non-significant similarity have no coloring.

There were many cases in which the similarity between signatures associated with two different contrasts was significantly greater than expected on the basis of chance (Fig. 5) (see Methods). This was generally true with regard to comparisons between any two dwarf mutation contrasts (e.g., snell5 vs. little3), but was less often the case with respect to comparisons between any two dietary treatments (e.g., ros vs. lowfat1). For every contrast, however, there was some evidence that its signature overlapped significantly with that of at least one other contrast (Fig. 5). The effects of gender overlapped significantly with those of the dwarf mutations as well as the four CR treatments. Additionally, differential expression signatures associated with lowfat diet treatments were significantly similar to those of dwarf mutations (especially the *Ghrhr*^*lit*/*lit *^mutation) and multiple CR treatments (see Fig. 5).

The "age" contrast provides an indication of how young livers (4 months) differ from aged livers (32 months) at the gene expression level. Genetic alterations or dietary manipulations with signatures similar to that of the age contrast can be viewed as inducing a "reversal" of the aging process, at least insofar as gene expression levels in mouse liver are concerned. From Figure 3, it is clear that the age signature has fairly weak overlap with those of other contrasts. Overall, however, the age signature clusters together with dietary manipulations in a branch that includes CR and CR mimetic compounds (Fig. 3). This suggests that, in comparison to the dwarf mutations, the effects of CR and some CR mimetics more closely resemble a shift of the liver transcriptome towards a youthful state. Additionally, whereas the differential expression signature of the age contrast was significantly similar to those of some CR treatments, this wasn't the case for any contrasts involving dwarf mutants (see Fig. 5).

### Longevity-associated genes I: long-lived dwarf mutants

**Figure 6:**
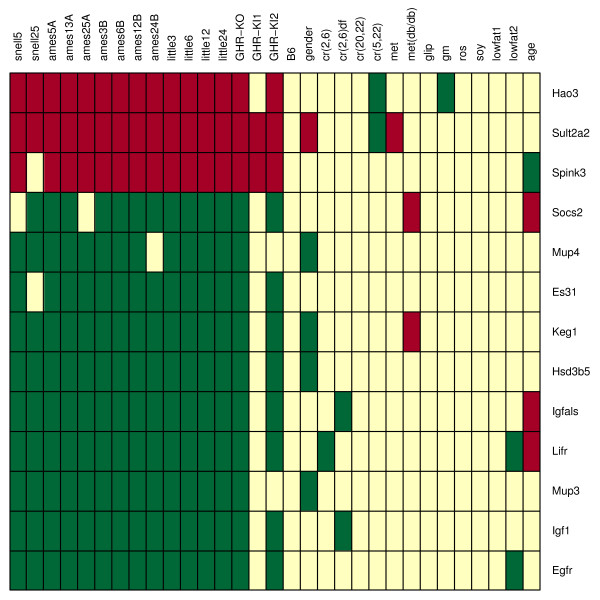
**Longevity-associated genes I**. Listed genes are those that are differentially expressed with respect to each of four-long lived dwarf models (Snell, Ames, Little, GHR-KO). Each row corresponds to an individual candidate gene, while each column corresponds to one of the contrasts listed in Table 1. Red squares indicate significant upregulation, while green squares indicate significant downregulation.

A total of 13 genes were differentially expressed with respect to all four long-lived mouse models (Fig. 6). Most of these genes (10/13) were downregulated with respect to all four models, while only three genes were upregulated (Hao3, Sult2a2, Spink3). Six of the identified genes are localized to extracellular space, which is a significantly unlikely result based on gene ontology cellular component analysis (P < 0.01). Within the extracellular space, these six genes influence in a diversity of processes, including protein binding and transport (Mup4, Spink3), signaling pathways that regulate cell proliferation (Igf1, Lifr, Igfals) and detoxification (Es31). Significantly overrepresented biological process gene ontology terms included steroid metabolism, positive regulation of cell proliferation, positive regulation of cellular process, enzyme linked receptor protein signaling pathway, electron transport, and cellular morphogenesis (P < 0.05). The molecular function gene ontologies pheromone binding and oxiodreductase activity (acting on CH-OH group of donors) were also significantly overrepresented (P < 0.01).

**Figure 7:**
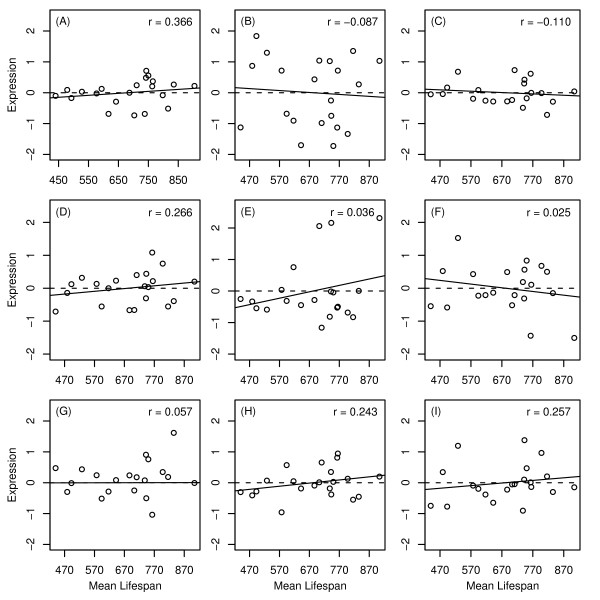
**Candidate gene expression versus mean lifespan I**. The expression level of nine candidate genes was examined among 21 BxD mouse strains. Expression data was generated by Williams et al. [30] (GEO series GSE6621). Lifespans of BxD strains were assayed by Gelman et al. [31]. The dashed horizontal line indicates the average gene expression level for each gene, while the solid line represents the least-squares regression estimate. Individual plots are shown for (A) Hao3, (B) Sult2a2, (C) Spink3, (D) Socs2, (E) Mup4, (F) Igfals, (G) Lifr, (H) Igf1 and (I) Efgr. The spearman rank correlation between expression and mean lifespan is shown in the upper right corner of each plot.

Williams et al. [30] have recently reported on baseline expression levels of liver organs from 31 BxD mouse strains (GEO Series GSE6621). The lifespan of 21 of these strains had previously been measured by Gelman et al. [31]. Using these two data sources, the relationship between expression levels of candidate genes and mean lifespan across BxD mouse strains was examined (Fig. 7). This analysis provided some slight, though non-significant, additional support for a role of Hao3 in lifespan determination (Fig. 7a). Hao3 is a peroxisome-targeted hydroxyacid oxidase [32], and was significantly upregulated with respect to all four long-lived dwarf models. Correspondingly, long-lived BxD strains exhibited higher Hao3 expression (*r*_*s *_= 0.366). Taken alone, this correlation was marginally significant (P = 0.051), but was non-signficant following Benjamini-Hochberg adjustments for multiple testing (P = 0.322).

### Longevity-associated genes II: dwarf mutants and CR

The same mechanisms underlying extended lifespan in dwarf mutants may contribute to the longevity extension that results from CR [23-25]. Of genes differentially expressed in all four long-lived models, only three were also differentially expressed with respect to at least one CR contrast (Igf1, Igfals and Lifr) (Fig. 6). To the extent that longevity extension in dwarf mutants has a common basis with longevity extension by CR, these three genes are the most well supported longevity-associated genes identified by this analysis.

**Figure 8:**
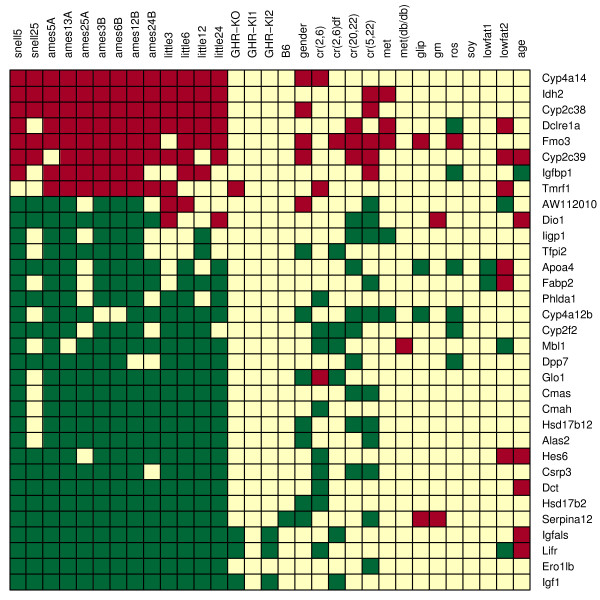
**Longevity-associated genes II**. Listed genes are those that are differentially expressed with respect to at least three of four long-lived dwarf models (Snell, Ames, Little, GHR-KO), and with respect to at least one of the four caloric restriction contrasts. Each row corresponds to an individual candidate gene, while each column corresponds to one of the contrasts listed in Table 1. Red squares indicate significant upregulation and green squares indicate significant downregulation.

Previous criteria was altered to obtain an expanded list of gene candidates that are differentially expressed with respect to long-lived dwarf models and under CR. In particular, genes differentially expressed with respect to at least three of four long-lived models, as well as with respect to at least one of the four CR contrasts were identified, which yielded 30 additional candidate genes (Fig. 8). Significantly overrepresented biological process gene ontology terms among these genes included electron transport, secondary metabolism, amine metabolism, steroid biosynthesis, and establishment of localization (P < 0.05). The cellular components endoplasmic reticulum, microsome, membrane fraction and extracellular space were also significantly overrepresented (P < 0.01). A wide range of molecular function gene ontology terms were overrepresented, the most significant of which were monooxygenase activity, heme binding, iron ion binding and insulin-like growth factor binding (P < 0.01).

**Figure 9:**
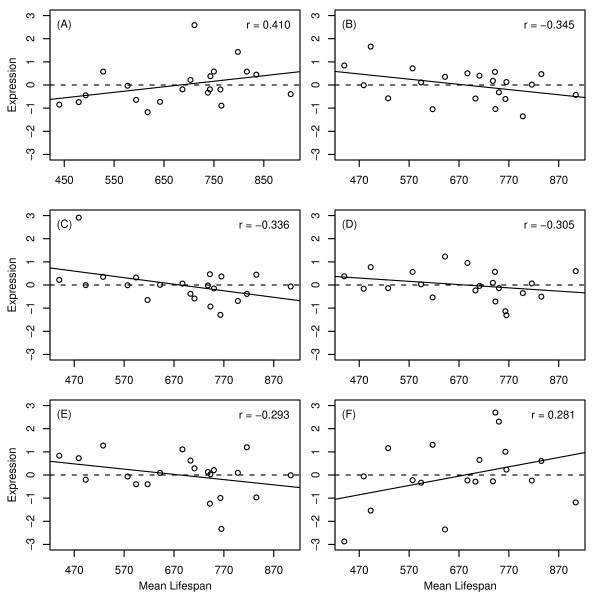
**Candidate gene expression versus mean lifespan II**. The expression level of six candidate genes was examined among 21 BxD mouse strains (see Fig. 6 caption). The dashed horizontal line indicates the average gene expression level for each gene, while the solid line indicates the estimates least-squares regression estimate. Individual plots are shown for (A) Fmo3, (B) Ero11b, (C) Serpina12, (D) Hes6, (E) Cyp2f2 and (F) Cyp4a14. The spearman rank correlation between expression and mean lifespan is shown in the upper right corner of each plot.

The expression level of flavin-containing monoxygenase 3 (Fmo3) was positively correlated with mean lifespan among 21 BxD strains (*r*_*s *_= 0.410, see Fig. 9A). This correlation was significant when taken alone (P = 0.033), but not following p-value adjustment for multiple testing among all 33 genes considered (P = 0.366). In addition to Fmo3, five other genes listed in Figure 7 exhibited marginally significant correlations prior to multiple testing adjustment (Ero11b, Serpina12, Hes6, Cyp2f2 and Cyp4a14) (0.063 < P < 0.108; see Fig. 9B–9F). In each case, the relationship between baseline expression level and mean lifespan was consistent with that expected based on differential expression patterns in dwarf models and under CR (compare Fig. 8 to Fig. 9).

## Discussion

Comparative analysis of multiple microarray datasets may provide insight not obtainable through analysis of any one dataset individually. This study presented a side-by-side comparison of microarray datasets generated from more than ten different studies of mouse liver organs, along with an analysis of each dataset using a consistent statistical methodology. The results provide a comprehensive view of how the effects of life-extending dwarf mutations, caloric restriction (CR), CR mimetic compounds, low fat diets, gender and aging are related at the level of gene expression. Comparisons among these various effects, based on expression patterns of more than 8500 genes, are of interest from multiple perspectives and shed light on a range of issues related to mammalian aging [e.g., see discussions in [16,19,23,24,33-35]]. A total of 43 candidate longevity-associated genes were identified based upon common differential expression patterns among four long-lived dwarf models, or between dwarf models and CR treatments. Each gene was identified across multiple independent experiments, and is therefore very unlikely to be a false positive identification. The set of longevity-associated genes identified in this analysis therefore provides well-supported targets for future experimental investigation as well as potential clues for understanding the functional basis of lifespan extension in mammalian systems.

The *GHR-KO *mutation was associated with much smaller transcriptional effects than mutations carried by Snell, Ames and Little mice. This result is surprising, particularly since GHR-KO and Little mice both carry mutations that specifically affect GH signaling. Little mice are GH deficient because the pituitary is unresponsive to GHRH, while GHR-KO mice have elevated GH levels and lack GH receptor. Transcriptional changes associated with both mutants should therefore reflect inhibition of GH signaling and, on this basis, it might be expected that the *lit *and *GHR-KO *mutations have similar effects on gene expression patterns. This was not the case, however, given that 500–600 genes were differentially expressed with respect to Little mice, while fewer than 50 genes were differentially expressed with respect to GHR-KO mice. This difference does not reflect sample size and statistical power disparities between experiments (see Table 2), and moreover, agrees with an earlier study that found very few genes (10 of 2352) differentially expressed between GHR-KO mice and normal controls [23]. This observation is also consistent with phenotypic data, which indicate that Little and GHR-KO mice exhibit dissimilar magnitudes of lifespan extension [9,15], as well as differing developmental weight-gain patterns [15]. It is possible that some disparity between differential expression signatures of GHR-KO and Little mice can be attributed to technical differences in sample preparation and array hybridizations. Another possibility is that the difference is due to levels of circulating growth hormone, which is nearly absent in Little mice and elevated in GHR-KO mice [9,15]. This difference would influence gene expression patterns in liver if GH has systemic or local effects independent of the GH receptor. These considerations suggest that, in comparison to other dwarf mutants, GHR-KO mice may provide a more useful model for the purpose of elucidating the mechanisms of longevity extension in mammals. GHR-KO mice exhibit lifespan extension that is comparable to that associated with Snell, Ames and Little mice. However, since the downstream transcriptional effects of the GHR-KO mutation are much less extensive than those of other long-lived models, understanding how this mutation contributes to longevity extension may prove less difficult.

Dwarf mutations and CR both extend lifespan in mice, but the degree to which this effect is mediated by common mechanisms remains unclear [23-25]. With regard to mouse liver tissue, this study reveals both similarities and differences between the effects of dwarf mutations and CR. The overall similarity between differential expression signatures associated with dwarf mutations and CR is significantly larger than expected on the basis of chance (Fig. 5). This is reflected by the 33 genes that exhibit parallel differential expression patterns in most long-lived mutants and under at least one CR treatment (Fig. 8). On the other hand, results of this analysis suggest that dwarf mutations have a much larger and more consistent effect on IGF-I expression than CR treatments (as well as potential CR mimetic compounds). This result was surprising, since several studies have shown that IGF-I expression and protein levels decline under CR [36-38]. It was further surprising that of the four CR treatments, IGF-I expression was only decreased significantly when CR was applied to dwarf mice (which already exhibit IGF-I deficiency). Taken together, these results suggest that life-extending dwarf mutations have mechanisms in common with CR, but that IGF-I suppression may not necessarily be included among these shared mechanisms. A limitation related to this inference is that the effects of CR treatments on expression patterns vary considerably among different studies, depending on the duration of caloric restriction, age at which necropsies are performed and the laboratory in which CR is carried out (see Figs. 3 and 4). A robust generalization regarding how the transcriptional effects of dwarf mutations relate to those of CR would therefore require even more data from CR mice than was analyzed in the present study.

Candidate longevity-associated genes were identified based on one of either two premises. The first premise is that lifespan extension in Snell, Ames, Little and GHR-KO mice is due to shared GH/IGF-I alterations, such that genes exhibiting parallel transcriptional changes in all four models are likely to play an important role in longevity. The second premise is that longevity extension in dwarf models and under CR is achieved through common mechanisms, suggesting that genes with similar expression changes in multiple dwarf models and under CR are potentially important for longevity. There is evidence in favor of both of these premises, and both have been endorsed in previous analyses [17,18,20,23]. It should be noted, however, that since the functional basis of mammalian lifespan is not well understood, the validity of each premise has not been established with certainty. Furthermore, all data examined in this study were generated from liver tissue. Liver tissue is the primary manufacturing site of IGF-I, but it is nonetheless possible that expression patterns in other tissues are of equal or greater importance in determining longevity. For instance, fibroblasts of long-lived mice are more stress-resistant than those of normal controls, suggesting that dwarf mutations affect a wide range of tissue and cell types [10,11]. In fact, reduced IGF-I levels in circulation may be the main factor behind extended lifespan in dwarf mice, such that with respect to liver, the only expression change consequential for longevity determination is that of the IGF-I transcript. These considerations should be weighed when evaluating the potential role of candidate genes in determining lifespan and rates of aging in mice and other mammalian species.

Only three genes were differentially expressed with respect to all four long-lived dwarf models with corresponding effects under at least one CR treatment (IGF-I, Igfals and Lifr). Both Igf1 and Igfals have a well-documented role in lifespan determination [39,40], but a potential role of leukemia inhibitory factor receptor (Lifr) in longevity has not been widely explored. Lifr expression was downregulated at all ages in four long-lived dwarf models, by short-term CR and by low-fat diet. Determining whether Lifr downregulation in liver contributes to longevity extension represents a difficult task, since Lifr is highly pleiotropic and has been associated with a wide range of biological effects. While there are several molecules that Lifr may interact with [41-43], Lifr expression is a primary determinant of cellular responsiveness to leukemia inhibitory factor (Lif) [44]. The Lif glycoprotein is a member of the IL-6 type cytokine family and exhibits a wide range of effects among different cell types. At a systemic level, excess Lif has been associated with low body weight, hypermotility, overgrowth of bone, calcification of several organs, loss of spermatocytes in males and severe loss of adipose tissue [44], and in the liver specifically, Lif has been found to stimulate triglyceride secretion [45]. Interestingly, however, *Lif(-/-) *mutants have been found to develop normally and possess generally good health, although pregnancy does not occur in females due to problems with blastocyst implantation [46]. To some degree, inhibition of Lif/Lifr signaling may be countered in long-lived mice by down-regulation of Socs2 (suppressor of cytokine signaling) [47,48], which was also down-regulated in all four long-lived dwarf models. Declines in Lifr expression may increase sensitivity to drug-induced liver disease [49], which in general is contrary to the stress resistance characteristics of long-lived mice [11], but is consistent with one study that found decreased resistance to acetaminophen toxicity in multiple dwarf strains [50].

Fmo3 and Cyp2f2 were differentially expressed with respect to most (3/4) long-lived dwarf models and also under the majority (3/4) of CR treatments that were examined. In addition, the expression level of both genes varied with mean lifespan among 21 BxD recombinant inbred mouse strains in a direction consistent with differential expression analyses. Since both genes are monooxygenases with established roles in drug metabolism, their expression in liver could mediate the life-extending effects of dwarf-mutations and CR through similar mechanisms. Fmo3 is a flavin containing monoxygenase for which expression levels are elevated in long-lived dwarf mutants, female mice, CR mice and mice treated with each of three different CR mimetic compounds (metaformin, glipizide and rosiglitazone). The Fmo3 gene has received considerable attention in studies of human populations, since it exhibits extensive polymorphism among individuals and may be a factor promoting differential drug response [51]. With regard to aging processes, a plausible hypothesis is that elevated Fmo3 levels in liver contributes to extended longevity by increasing stores of glutathione (GSH) and glutathione-S-transferase (GST), which then leads to enhanced resistance to oxidative stress. This hypothesis is appealing, since Fmo3 has an important role in sulfoxidation of methionine [52,53], and it has been found that long-lived Ames mice exhibit elevated methionine metabolism [54], which may underlie increased GSH, GST and oxidative stress resistance in long-lived models [54].

Cyp2f2 is a cytochrome P450 enzyme, and in contrast to Fmo3, its expression was downregulated in long-lived and CR mice. Several other cytochrome P450 enzymes exhibited differential expression, with slightly less consistency, among multiple long-lived models and under CR treatments (Cyp4a14, Cyp2c38, Cyp2c39 and Cyp4a12b). Although Cyp2f2 displayed the most consistent differential expression pattern, it is unlikely that Cyp2f2 would have a unique role in mammalian aging apart from other cytochrome P450 enzymes. Cytochrome P450 enzymes are involved in xenobiotic detoxification and are catalysts for a large number of metabolic reactions. It is unclear how this system may influence aging in mammals, but multiple P450 enzymes are also differentially expressed in long-lived *C. elegans daf-2 *mutants, which has led to the suggestion that P450 enzymes slow aging by reducing damage generated from toxic compounds [55,56]. From the standpoint of mammalian aging, it is of interest that some P450 enzymes, including Cyp2f2, are regulated by peroxisome proliferator-activated receptors (PPARs) [57,58]. Cytochrome P450 enzymes may therefore be part of a broader cellular response that has previously been associated with longevity in long-lived and CR mice [59]. An interesting pattern is that, in many cases, P450 enzyme expression changes in long-lived mice and under CR were mirrored by expression differences in females relative to males. In a comprehensive analysis of 41 cytochrome P450 enzyme genes, it was found that 14 genes exhibited this pattern to some degree (data not shown). This result may be attributable to the role of GH as a factor regulating cytochrome P450 expression [60].

The correlation structure of expression patterns among genes may be as informative as differential expression analyses but is often an underexploited aspect of microarray datasets. Similarity among expression patterns across many conditions can be used, for example, to make inferences and generate testable hypotheses regarding interactions between genes [61,62]. Along these lines, genes with expression patterns among contrasts that closely corresponded with those of the IGF-I transcript were identified in this study. This analysis identified Snm1 (DNA cross-link repair 1a; also called Dclre1a) as a potential IGF-I regulated or co-regulated gene (see Fig. 2 and additional file 2). The induction pattern of Snm1 is opposite that of IGF-I across contrasts, such that its expression is increased in proportion to IGF-I inhibition. *Snm1(-/-) *mice have reduced lifespan, which is primarily due to elevated mortality from bacterial infection and cancer, suggesting that Snm1 is a tumor suppressor with an immunological role [63]. At the cellular level, overexpression of Snm1 in yeast increases resistance to genotoxic stress agents that induce DNA cross-links [64], and stem cells derived from *Snm1(-/-) *mice are sensitive to the cross-linking agent mitomycin C [65]. These results suggest that elevated Snm1 expression may be a causal factor underlying resistance to the DNA-alkylating agent methyl methanesulfonate that has previously been found in cell lines derived from long-lived mice [10,11]. It would thus be worthwhile to evaluate whether Snm1 expression is elevated in such cell lines (as in hepatic tissue), and if so, whether these cells are resistant to DNA cross-link inducing agents (e.g., nitrogen mustard, cisplatin). Interestingly, recent studies report that DNA repair mutations leading to accelerated senescence phenotypes are, like long-lived dwarf mutations, associated with IGF-I inhibition [66]. It is therefore surprising that in long-lived and CR mice, IGF-I inhibition is associated with elevated Snm1 expression, which may enhance DNA repair and promote genomic stability.

The present study has demonstrated analytical approaches for the comparative analysis of microarray datasets that may have application in other contexts. Microarray analyses based upon individual datasets often identify an exceptionally large number of genes, which limits the utility of microarray data as a tool for selecting candidates in follow-up studies. Recently, however, public repositories of high-quality microarray data have been established [67,68], and statistical methods aimed at comparative analysis of these resources continue to be developed [69-72]. Increasingly, therefore, comparative analysis provides a feasible approach for filtering out false-positive identifications and identifying transcripts most consistently supported across multiple experiments. This yields a set of candidate genes that is necessarily smaller and more tractable for subsequent experimental investigation, and moreover, each identified gene is more likely to represent a statistically significant finding. Furthermore, apart from the identification of individual gene candidates, comparison of expression datasets provides unique insight into genome-wide patterns among studies.

The maximum human lifespan is approximately twice as large as the maximum chimpanzee lifespan [73], which is more than ten times larger than the maximum mouse lifespan [2]. The evolutionary lineage connecting humans to chimpanzees to mouse may therefore represent a remarkable instance of lifespan extension and delayed rates of aging. Inhibition of IGF-I signaling is (at present) the only known genetic manipulation that extends lifespan in multiple species, so it is tempting to ask whether this pathway has contributed to evolutionary extension of lifespan within the mammalian lineage. It is interesting to note that, contrary to longevity extension via nearly all IGF-I signaling mutations, lifespan extension in the mouse-chimp-human lineage has been accompanied by an *increase *in body size. This observation is encouraging with regard to the possibility of developing interventions that delay the onset of age-related disease in humans without undesirable consequences (e.g., dwarfism). Clarke et al. [74] used sequence data and *d*_*N*_/*d*_*S *_ratios to identify mouse-chimp-human orthologs that exhibit more rapid evolutionary change than expected based upon neutral substitution models (i.e., positive selection). Supplemental data from their study provides no indication of positive selection with respect to IGF-I, providing little indication that IGF-I sequence changes have been consequential during human evolution. It is interesting to note, however, that Dio1 (iodothyronine deiodinase), which is downregulated in most dwarf mouse strains and under CR (see Fig. 8), has been associated with positive selection and accelerated evolution within the mouse-chimp-human lineage (see supplemental data from [74]). This is also the case for Papp-A (pregnancy associated plasma protein A) [74], which was not identified in the present study, but is of importance since Papp-A-KO mice exhibit diminished IGF-I bioactivity and extended lifespan [75]. These results are suggestive, but given the many phenotypic differences between mice, chimpanzees and humans (besides lifespan), this evolutionary criterion does not establish the functional significance of Dio1, Papp-A or other positively selected genes.

These findings provide a useful reference point for future experimental studies of long-lived dwarf mice and mammalian aging. It would be of interest, for example, to determine whether genes identified in the present study are differentially expressed with respect to other long-lived mouse strains for which data is not currently available. Similar gene expression changes may be found in liver tissue of p66^shc ^knockouts [76], IGF-IR knockouts [77], Klotho transgenic mice [78] and Papp-A-KO mice [75], since each of these long-lived models carry mutations that also inhibit the IGF-I signaling pathway. Because these mutations inhibit IGF-I signals at points further downstream than those considered in this study, expression data generated from these models could be combined with results of the present study to assemble a more comprehensive picture of hepatic IGF-I signaling. Since the currently known life-extending mutations are maintained on differing genetic backgrounds [1], evaluating potential background effects on lifespan and gene expression patterns will also be critical for elucidating hepatic IGF-1 signaling pathways [79]. Ultimately, however, it is important to develop a systemic model of GH/IGF-I signaling, with further evaluation of the respective roles of IGF-I and GH in extended longevity. IGF-I is manufactured in a wide variety of tissue types, and in some cases IGF-I production may be independent of GH signaling [80]. It is therefore important to consider not only local effects of GH reduction on gene expression in liver, but also effects of GH/IGF-I in other tissue types. Studies of long-lived C. elegans mutants, for instance, have suggested that IGF-I signaling in nervous tissue may be consequential in determining lifespan [81]. It would therefore be useful to evaluate whether genes identified in the present study are also differentially expressed in non-hepatic tissues of Ames, Snell, Little or GHR-KO mice.

## Conclusion

Several mutations are known to increase longevity in mouse, but most of these mutations are associated with pleiotropic effects, which include traits that are undesirable from a therapeutic perspective (e.g., dwarfism). A key challenge in future work towards potential longevity-promoting therapeutic compounds is to decouple the positive life-extending effects of IGF-I inhibition from diminutions in growth and body size. Determining whether this is a possibility requires a more complete functional understanding of longevity extension in long-lived mutant mouse models. The present study has taken steps in this direction by presenting comparisons between the transcriptional effects of dwarf mutations and those of dietary treatments, gender and aging. Among other findings, these comparisons reveal that *lit/lit *and *GHR-KO *mutations exhibit transcriptional effects of surprisingly different magnitude, transcriptional effects of CR (and some CR mimetics) resemble aging more closely then those of dwarf mutations, and that the transcriptional effects of gender and lowfat diets overlap significantly with those of dwarf mutations and CR. A total of 43 genes with the highest levels of support as longevity-associated transcripts were identified in this context. The main value of identified genes is the potential that, as putative downstream elements in the IGF-I signaling pathway, some genes may be more directly (and functionally) involved in longevity determination than upstream IGF-I signaling components. Experimental studies focusing on identified genes may therefore enhance our functional understanding of how mutations affecting IGF-I signaling lead to extended lifespan and deceleration of aging in mammals.

## Methods

Gene expression datasets analyzed in this study were selected from those available in the Gene Expression Omnibus (GEO) and ArrayExpress depositories for MIAME-compliant microarray data [67,68]. Expression datasets were generated using one of three Affymetrix microarray platforms (MG-U74A, MOE430A, 430 2.0). Expression values were calculated by either the MAS 5.0 algorithm or Robust Multichip Average [82]. When raw CEL files were available, MAS 5.0 generated datasets were re-normalized using RMA.

Differential expression testing was performed using the Limma linear modeling package available in the R Bioconductor software suite [83]. This approach fits a linear model to expression values associated with each individual gene, and the distribution of sample residual variances among all genes is used to stabilize the residual variance estimates of individual genes by shrinkage towards a prior value. This limits false-positive gene identifications arising from underestimated residual variances for datasets with low levels of replication in each experimental treatment. Separate linear model analyses were conducted for contrast groups sharing the same superscript in Table 1. Expression values were not combined, therefore, when expression was assayed using different platforms or when experiments were performed in different laboratories. For each contrast, P-values were adjusted across genes using the Benjamini-Hochberg method [84]. When multiple contrasts were specified for a single dataset, the nested-F test approach was used to evaluate the significance of moderated t-statistics associated with each individual contrast [83].

Longevity-associated genes were identified based on common differential expression patterns among contrasts using criteria described in the Results section. The GOstats R Bioconductor package was used evaluate whether particular gene ontology terms were overrepresented with respect to identified genes [85]. The gene universe in each test was defined as the 8525 probesets included in the analysis, i.e., those that could be matched among the MG-U74A, MOE430A and 430 2.0 Affymetrix platforms [85].

### Differential expression signature similarity metric

A similarity measure was developed to compare gene expression signatures associated with different contrasts. This similarity is intuitive in terms of Venn diagrams, where the similarity between two signatures is proportional to the overlap between sets of genes differentially expressed with respect to each contrast individually. This notion was generalized in order to define a measure of similarity that can be used for clustering signatures associated with different contrasts. Consider two signatures that have been generated by two contrasts *α *and *β*. Using the notations defined in Table 3, the signatures contain *n*_+,+ _+ *n*_-,- _genes with identical differential expression patterns, *n*_+,- _+ *n*_-,+ _genes with opposite differential expression patterns, and *n*_+,0 _+ *n*_0,+ _+ *n*_-,0 _+ *n*_0,- _genes differentially expressed with respect to just one of the two contrasts. Given these values, Equation (1) defines a measure of similarity (*s*) between two different signatures, where 0 ≤ *s *≤ 1.

**Table 3:** Notations associated with Equation (1)

		Contrast *α*		
		
		Upregulated	Downregulated	*H*_0 _not rejected
Contrast *β*	Upregulated	*n*_+,+_	*n*_-,+_	*n*_0,+_
	Downregulated	*n*_+,-_	*n*_-,-_	*n*_0,-_
	*H*_0 _not rejected	*n*_+,0_	*n*_-,0_	*n*_0,0_

The null hypothesis *H*_0_: *μ*_*Ai *_= *μ*_*Bi *_was evaluated for *N *genes with respect to contrasts *α *and *β *(see text). With respect to each contrast individually, genes are either upregulated, downregulated, or *H*_0 _is not rejected. With respect to both contrasts *α *and *β*, therefore, all *N *genes are classified into one of nine categories. The number of genes assigned to each category is indicated by the values of *n *given in the table.

s=n+,++n−,−n+,++n−,−+Min[(n+,0+n−,0),(n0,++n0,−)]
 MathType@MTEF@5@5@+=feaafiart1ev1aaatCvAUfKttLearuWrP9MDH5MBPbIqV92AaeXatLxBI9gBaebbnrfifHhDYfgasaacH8akY=wiFfYdH8Gipec8Eeeu0xXdbba9frFj0=OqFfea0dXdd9vqai=hGuQ8kuc9pgc9s8qqaq=dirpe0xb9q8qiLsFr0=vr0=vr0dc8meaabaqaciaacaGaaeqabaqabeGadaaakeaacqWGZbWCcqGH9aqpdaWcaaqaaiabd6gaUnaaBaaaleaacqGHRaWkcqGGSaalcqGHRaWkaeqaaOGaey4kaSIaemOBa42aaSbaaSqaaiabgkHiTiabcYcaSiabgkHiTaqabaaakeaacqWGUbGBdaWgaaWcbaGaey4kaSIaeiilaWIaey4kaScabeaakiabgUcaRiabd6gaUnaaBaaaleaacqGHsislcqGGSaalcqGHsislaeqaaOGaey4kaSIaeeyta0KaeeyAaKMaeeOBa4Maei4waSLaeiikaGIaemOBa42aaSbaaSqaaiabgUcaRiabcYcaSiabicdaWaqabaGccqGHRaWkcqWGUbGBdaWgaaWcbaGaeyOeI0IaeiilaWIaeGimaadabeaakiabcMcaPiabcYcaSiabcIcaOiabd6gaUnaaBaaaleaacqaIWaamcqGGSaalcqGHRaWkaeqaaOGaey4kaSIaemOBa42aaSbaaSqaaiabicdaWiabcYcaSiabgkHiTaqabaGccqGGPaqkcqGGDbqxaaaaaa@60A6@

There are several plausible similarity measures between two different signatures. A main advantage of the similarity measure defined by Equation (1) is that similarity not increased by the *n*_0,0 _genes that are not differentially expressed with respect to either contrast. Thus, emphasis is placed only on the minority of genes that are differentially expressed. Additionally, the denominator of Equation (1) is configured such that two signatures may be similar even if they differ greatly in the total number of genes that are differentially expressed, provided that the contrast associated with less differential expression yields approximately *n*_+,+ _+ *n*_-,- _differentially expressed genes. This is sensible in consideration of the fact that the number of differentially expressed genes generated by a given contrast depends on the sample sizes used in the experimental treatments being compared. For signature comparisons in the present study, the value *n*_+,- _+ *n*_-,+ _was negligible and therefore not included in Equation (1). If *n*_+,- _+ *n*_-,+ _had been large, however, it would have been appropriate to add this term to the denominator of Equation (1) when evaluating similarity between signatures.

Differential expression signatures for each contrast listed in Table 1 were compared using the similarity measure defined by Equation (1). The distance between two signatures was defined as 1 – *s *and a hierarchical cluster analysis of signatures associated with the 31 contrasts specified in Table 1 was performed (Fig. 4). Groups of signatures were joined using the average distance method. This yielded a dendrogram providing an indication of which contrasts were associated with similar differential expression patterns.

### Statistical evaluation of overlap between differential expression signatures

A statistical procedure was developed to evaluate whether differential expression signatures associated with two different contrasts exhibited a significant level of similarity.  Among the 31 contrasts, the correspondence between all 31(31-1)/2 = 465 pairwise combinations of signatures was evaluated. The procedure used is similar to that proposed by Smid et al. [86]. The null hypothesis for this statistical test is that, for any two contrasts *α *and *β*, the probability that a gene is differentially expressed with respect to contrast *α *is independent of the probability that a gene is differentially expressed with respect to contrast *β*. It should be noted that the three contrasts with a *d *superscript in Table 1 were defined using a common control treatment as a reference. It is thus expected *a priori *that differential expression signatures of these three contrasts will exhibit association beyond that stated by the null hypothesis defined above. This is also true of the seven contrasts carrying an *h *superscript in Table 1.

The value *T *defined below is proportional to *s *and serves as the test statistic.

*T *= *n*_+,+ _+ *n*_-,-_

A total of *N *genes are considered in the analysis. It is given that n+α
 MathType@MTEF@5@5@+=feaafiart1ev1aaatCvAUfKttLearuWrP9MDH5MBPbIqV92AaeXatLxBI9gBaebbnrfifHhDYfgasaacH8akY=wiFfYdH8Gipec8Eeeu0xXdbba9frFj0=OqFfea0dXdd9vqai=hGuQ8kuc9pgc9s8qqaq=dirpe0xb9q8qiLsFr0=vr0=vr0dc8meaabaqaciaacaGaaeqabaqabeGadaaakeaacqWGUbGBdaqhaaWcbaGaey4kaScabaacciGae8xSdegaaaaa@30C6@ genes upregulated with respect to contrast *α*, n−α
 MathType@MTEF@5@5@+=feaafiart1ev1aaatCvAUfKttLearuWrP9MDH5MBPbIqV92AaeXatLxBI9gBaebbnrfifHhDYfgasaacH8akY=wiFfYdH8Gipec8Eeeu0xXdbba9frFj0=OqFfea0dXdd9vqai=hGuQ8kuc9pgc9s8qqaq=dirpe0xb9q8qiLsFr0=vr0=vr0dc8meaabaqaciaacaGaaeqabaqabeGadaaakeaacqWGUbGBdaqhaaWcbaGaeyOeI0cabaacciGae8xSdegaaaaa@30D1@ genes downregulated with respect to contrast *α*, n+β
 MathType@MTEF@5@5@+=feaafiart1ev1aaatCvAUfKttLearuWrP9MDH5MBPbIqV92AaeXatLxBI9gBaebbnrfifHhDYfgasaacH8akY=wiFfYdH8Gipec8Eeeu0xXdbba9frFj0=OqFfea0dXdd9vqai=hGuQ8kuc9pgc9s8qqaq=dirpe0xb9q8qiLsFr0=vr0=vr0dc8meaabaqaciaacaGaaeqabaqabeGadaaakeaacqWGUbGBdaqhaaWcbaGaey4kaScabaacciGae8NSdigaaaaa@30C8@ genes upregulated with respect to contrast *β*, and n+β
 MathType@MTEF@5@5@+=feaafiart1ev1aaatCvAUfKttLearuWrP9MDH5MBPbIqV92AaeXatLxBI9gBaebbnrfifHhDYfgasaacH8akY=wiFfYdH8Gipec8Eeeu0xXdbba9frFj0=OqFfea0dXdd9vqai=hGuQ8kuc9pgc9s8qqaq=dirpe0xb9q8qiLsFr0=vr0=vr0dc8meaabaqaciaacaGaaeqabaqabeGadaaakeaacqWGUbGBdaqhaaWcbaGaey4kaScabaacciGae8NSdigaaaaa@30C8@ genes downregulated with respect to contrast *β*. Given these quantities, n+αN
 MathType@MTEF@5@5@+=feaafiart1ev1aaatCvAUfKttLearuWrP9MDH5MBPbIqV92AaeXatLxBI9gBaebbnrfifHhDYfgasaacH8akY=wiFfYdH8Gipec8Eeeu0xXdbba9frFj0=OqFfea0dXdd9vqai=hGuQ8kuc9pgc9s8qqaq=dirpe0xb9q8qiLsFr0=vr0=vr0dc8meaabaqaciaacaGaaeqabaqabeGadaaakeaadaWcaaqaaiabd6gaUnaaDaaaleaacqGHRaWkaeaaiiGacqWFXoqyaaaakeaacqWGobGtaaaaaa@3205@ is the probability of upregulation with respect to contrast *α*, n−αN
 MathType@MTEF@5@5@+=feaafiart1ev1aaatCvAUfKttLearuWrP9MDH5MBPbIqV92AaeXatLxBI9gBaebbnrfifHhDYfgasaacH8akY=wiFfYdH8Gipec8Eeeu0xXdbba9frFj0=OqFfea0dXdd9vqai=hGuQ8kuc9pgc9s8qqaq=dirpe0xb9q8qiLsFr0=vr0=vr0dc8meaabaqaciaacaGaaeqabaqabeGadaaakeaadaWcaaqaaiabd6gaUnaaDaaaleaacqGHsislaeaaiiGacqWFXoqyaaaakeaacqWGobGtaaaaaa@3210@ is the probability of downregulation with respect to contrast *α*, n+βN
 MathType@MTEF@5@5@+=feaafiart1ev1aaatCvAUfKttLearuWrP9MDH5MBPbIqV92AaeXatLxBI9gBaebbnrfifHhDYfgasaacH8akY=wiFfYdH8Gipec8Eeeu0xXdbba9frFj0=OqFfea0dXdd9vqai=hGuQ8kuc9pgc9s8qqaq=dirpe0xb9q8qiLsFr0=vr0=vr0dc8meaabaqaciaacaGaaeqabaqabeGadaaakeaadaWcaaqaaiabd6gaUnaaDaaaleaacqGHRaWkaeaaiiGacqWFYoGyaaaakeaacqWGobGtaaaaaa@3207@ is the probability of upregulation with respect to contrast *β*, and n−βN
 MathType@MTEF@5@5@+=feaafiart1ev1aaatCvAUfKttLearuWrP9MDH5MBPbIqV92AaeXatLxBI9gBaebbnrfifHhDYfgasaacH8akY=wiFfYdH8Gipec8Eeeu0xXdbba9frFj0=OqFfea0dXdd9vqai=hGuQ8kuc9pgc9s8qqaq=dirpe0xb9q8qiLsFr0=vr0=vr0dc8meaabaqaciaacaGaaeqabaqabeGadaaakeaadaWcaaqaaiabd6gaUnaaDaaaleaacqGHsislaeaaiiGacqWFYoGyaaaakeaacqWGobGtaaaaaa@3212@ is the probability of downregulation with respect to contrast *β*. Under the null hypothesis stated above, n+αN
 MathType@MTEF@5@5@+=feaafiart1ev1aaatCvAUfKttLearuWrP9MDH5MBPbIqV92AaeXatLxBI9gBaebbnrfifHhDYfgasaacH8akY=wiFfYdH8Gipec8Eeeu0xXdbba9frFj0=OqFfea0dXdd9vqai=hGuQ8kuc9pgc9s8qqaq=dirpe0xb9q8qiLsFr0=vr0=vr0dc8meaabaqaciaacaGaaeqabaqabeGadaaakeaadaWcaaqaaiabd6gaUnaaDaaaleaacqGHRaWkaeaaiiGacqWFXoqyaaaakeaacqWGobGtaaaaaa@3205@ is independent of n+βN
 MathType@MTEF@5@5@+=feaafiart1ev1aaatCvAUfKttLearuWrP9MDH5MBPbIqV92AaeXatLxBI9gBaebbnrfifHhDYfgasaacH8akY=wiFfYdH8Gipec8Eeeu0xXdbba9frFj0=OqFfea0dXdd9vqai=hGuQ8kuc9pgc9s8qqaq=dirpe0xb9q8qiLsFr0=vr0=vr0dc8meaabaqaciaacaGaaeqabaqabeGadaaakeaadaWcaaqaaiabd6gaUnaaDaaaleaacqGHRaWkaeaaiiGacqWFYoGyaaaakeaacqWGobGtaaaaaa@3207@ and n−αN
 MathType@MTEF@5@5@+=feaafiart1ev1aaatCvAUfKttLearuWrP9MDH5MBPbIqV92AaeXatLxBI9gBaebbnrfifHhDYfgasaacH8akY=wiFfYdH8Gipec8Eeeu0xXdbba9frFj0=OqFfea0dXdd9vqai=hGuQ8kuc9pgc9s8qqaq=dirpe0xb9q8qiLsFr0=vr0=vr0dc8meaabaqaciaacaGaaeqabaqabeGadaaakeaadaWcaaqaaiabd6gaUnaaDaaaleaacqGHsislaeaaiiGacqWFXoqyaaaakeaacqWGobGtaaaaaa@3210@ is independent of n−βN
 MathType@MTEF@5@5@+=feaafiart1ev1aaatCvAUfKttLearuWrP9MDH5MBPbIqV92AaeXatLxBI9gBaebbnrfifHhDYfgasaacH8akY=wiFfYdH8Gipec8Eeeu0xXdbba9frFj0=OqFfea0dXdd9vqai=hGuQ8kuc9pgc9s8qqaq=dirpe0xb9q8qiLsFr0=vr0=vr0dc8meaabaqaciaacaGaaeqabaqabeGadaaakeaadaWcaaqaaiabd6gaUnaaDaaaleaacqGHsislaeaaiiGacqWFYoGyaaaakeaacqWGobGtaaaaaa@3212@. Consequently, the probability that a gene is jointly upregulated with respect to contrasts *α *and *β *is given by *p*_1_, while the probability that a gene is jointly downregulated with respect to contrasts *α *and *β *is given by *p*_2_.

p1=n+αn+βN2
 MathType@MTEF@5@5@+=feaafiart1ev1aaatCvAUfKttLearuWrP9MDH5MBPbIqV92AaeXatLxBI9gBaebbnrfifHhDYfgasaacH8akY=wiFfYdH8Gipec8Eeeu0xXdbba9frFj0=OqFfea0dXdd9vqai=hGuQ8kuc9pgc9s8qqaq=dirpe0xb9q8qiLsFr0=vr0=vr0dc8meaabaqaciaacaGaaeqabaqabeGadaaakeaacqWGWbaCdaWgaaWcbaGaeGymaedabeaakiabg2da9maalaaabaGaemOBa42aa0baaSqaaiabgUcaRaqaaGGaciab=f7aHbaakiabd6gaUnaaDaaaleaacqGHRaWkaeaacqWFYoGyaaaakeaacqWGobGtdaahaaWcbeqaaiabikdaYaaaaaaaaa@3AD3@

p2=n−αn−βN2
 MathType@MTEF@5@5@+=feaafiart1ev1aaatCvAUfKttLearuWrP9MDH5MBPbIqV92AaeXatLxBI9gBaebbnrfifHhDYfgasaacH8akY=wiFfYdH8Gipec8Eeeu0xXdbba9frFj0=OqFfea0dXdd9vqai=hGuQ8kuc9pgc9s8qqaq=dirpe0xb9q8qiLsFr0=vr0=vr0dc8meaabaqaciaacaGaaeqabaqabeGadaaakeaacqWGWbaCdaWgaaWcbaGaeGOmaidabeaakiabg2da9maalaaabaGaemOBa42aa0baaSqaaiabgkHiTaqaaGGaciab=f7aHbaakiabd6gaUnaaDaaaleaacqGHsislaeaacqWFYoGyaaaakeaacqWGobGtdaahaaWcbeqaaiabikdaYaaaaaaaaa@3AEB@

Random variable *X *denotes the number of jointly upregulated genes among all *N *genes, and random variable *Y *equals the number of jointly downregulated genes among all *N *genes, such that *T *= *X *+ *Y*. The distributions of *X *and *Y *are binomial under the null hypothesis.

P(X=x|p1)=(Nx)p1x(1−p1)N−x
 MathType@MTEF@5@5@+=feaafiart1ev1aaatCvAUfKttLearuWrP9MDH5MBPbIqV92AaeXatLxBI9gBaebbnrfifHhDYfgasaacH8akY=wiFfYdH8Gipec8Eeeu0xXdbba9frFj0=OqFfea0dXdd9vqai=hGuQ8kuc9pgc9s8qqaq=dirpe0xb9q8qiLsFr0=vr0=vr0dc8meaabaqaciaacaGaaeqabaqabeGadaaakeaacqWGqbaucqGGOaakcqWGybawcqGH9aqpcqWG4baEcqGG8baFcqWGWbaCdaWgaaWcbaGaeGymaedabeaakiabcMcaPiabg2da9maabmaabaqbaeqabiqaaaqaaiabd6eaobqaaiabdIha4baaaiaawIcacaGLPaaacqWGWbaCdaqhaaWcbaGaeGymaedabaGaemiEaGhaaOGaeiikaGIaeGymaeJaeyOeI0IaemiCaa3aaSbaaSqaaiabigdaXaqabaGccqGGPaqkdaahaaWcbeqaaiabd6eaojabgkHiTiabdIha4baaaaa@4A67@

P(Y=y|p2)=(Ny)p2y(1−p2)N−y
 MathType@MTEF@5@5@+=feaafiart1ev1aaatCvAUfKttLearuWrP9MDH5MBPbIqV92AaeXatLxBI9gBaebbnrfifHhDYfgasaacH8akY=wiFfYdH8Gipec8Eeeu0xXdbba9frFj0=OqFfea0dXdd9vqai=hGuQ8kuc9pgc9s8qqaq=dirpe0xb9q8qiLsFr0=vr0=vr0dc8meaabaqaciaacaGaaeqabaqabeGadaaakeaacqWGqbaucqGGOaakcqWGzbqwcqGH9aqpcqWG5bqEcqGG8baFcqWGWbaCdaWgaaWcbaGaeGOmaidabeaakiabcMcaPiabg2da9maabmaabaqbaeqabiqaaaqaaiabd6eaobqaaiabdMha5baaaiaawIcacaGLPaaacqWGWbaCdaqhaaWcbaGaeGOmaidabaGaemyEaKhaaOGaeiikaGIaeGymaeJaeyOeI0IaemiCaa3aaSbaaSqaaiabikdaYaqabaGccqGGPaqkdaahaaWcbeqaaiabd6eaojabgkHiTiabdMha5baaaaa@4A77@

When *Np*_1 _and *Np*_2 _are large, *P*(*X *= *x*) and *P*(*Y *= *y*) and their convolution *T *= *X *+ *Y *are approximately Normal under *H*_0_. When *Np*_1 _and *Np*_2 _are small, *P*(*X *= *x*) and *P*(*Y *= *y*) and their convolution *T *= *X *+ *Y *are approximately Poisson under *H*_0_. The significance of observed *T *values for each pair of contrasts by comparison to either Normal or Poisson cumulative distribution functions, depending on the magnitude of *Np*_1 _and *Np*_2_. This test was carried out for all pairwise combinations of the 31 contrasts evaluated, yielding a total of 31(31-1)/2 = 465 p-values. All p-values were confirmed by simulation analyses. To control for multiple testing, p-values were adjusted using the Benjamini-Hochberg method.

## Competing interests

The author(s) declares that there are no competing interests.

## Supplementary Material

Additional file 1 **Genes positively associated with IGF-I expression**. This file displays expression response profiles for the top 40 genes most positively associated with IGF-I induction patterns among all contrasts examined in this study (see Fig. 2). In each plot, the black line represents the IGF-I induction pattern among contrasts, and the red line represents the pattern associated with a gene that exhibits a closely matching induction pattern. Genes are presented in order of decreasing similarity to the IGF-I induction pattern. Following appropriate normalization to weight all contrasts equally, similarity was determined based on Euclidean distance between patterns. Click here for file

Additional file 2 **Genes negatively associated with IGF-I expression**. This file displays expression response profiles for the top 40 genes most negatively associated with IGF-I induction patterns among all contrasts examined in this study (see Fig. 2). In each plot, the black line represents the IGF-I induction pattern among contrasts, and the green line represents the pattern associated with a gene that exhibits an opposite induction pattern. Genes were selected by reflecting the induction pattern of IGF-I about the zero horizontal, and finding genes with an induction pattern most similar to this IGF-I reflection. Genes are presented in order of decreasing similarity to the IGF-I reflection. Following appropriate normalization to weight all contrasts equally, similarity was determined based on Euclidean distance. Click here for file
