# Supplementary material for: Gene expression profiling of long-lived dwarf mice: longevity-associated genes and relationships with diet, gender and aging
Source: BMC Genomics. 2007 Oct 3;8:353. doi: 10.1186/1471-2164-8-353 (PMC2094713; doi:10.1186/1471-2164-8-353)
Supplement: Additional file 1 — Genes positively associated with IGF-I expression. This file displays expression response profiles for the top 40 genes most positively associated with IGF-I induction patterns among all contrasts examined in this study (see Fig. 2). In each plot, the black line represents the IGF-I induction pattern among contrasts, and the red line represents the pattern associated with a gene that exhibits a closely matching induction pattern. Genes are presented in order of decreasing similarity to the IGF-I induction pattern. Following appropriate normalization to weight all contrasts equally, similarity was determined based on Euclidean distance between patterns. [file 1471-2164-8-353-S1.pdf]

Additional File 1:

Gene expression profiling of long−lived dwarf mice: longevity−associated genes and relationships with diet, gender and aging

William R. Swindell

This file displays expression response profiles for the top 40 genes most positively associated with IGF−1 induction patterns among all contrasts examined in this study (see Fig. 2 of paper). These genes are listed on pages 2−3 and plotted individually on pages 4−43 of this file. In each plot, the black line represents the IGF−1 induction pattern among contrasts, and the red line represents the pattern associated with a gene that exhibits a closely matching induction pattern. Genes are presented in order of decreasing similarity to the IGF−1 induction pattern. Following appropriate normalization to weight all contrasts equally, similarity was determined based on Euclidean distance between patterns.

Contact: William R. Swindell, wswindel@med.umich.edu

Symbol	Affymetrix ID	Distance
Mup3	101909_f_at	10.228
Es31	99941_at	10.777
Igfals	97987_at	10.843
Keg1	96938_at	13.074
Socs2	99475_at	13.306
Egfr	101842_g_at	13.626
Lifr	104658_at	13.866
C9	104424_at	14.119
Phlda1	160829_at	15.153
Dct	103597_at	16.138
Mup1	101566_f_at	16.545
Mup5	101635_f_at	16.669
Irf6	92440_at	17.046
Mcm10	103553_at	17.12
Fabp2	97889_at	17.225
Trp53inp2	160376_at	17.495
Serpine2	97487_at	17.521
Csad	99184_at	17.593
C730048C13Rik	103702_i_at	18.066
Hsd17b2	101891_at	18.173

Symbol	Affymetrix ID	Distance
Alas2	92768_s_at	18.371
Gpc1	104614_at	18.769
Hsd3b2	101659_at	18.938
Ero1lb	103531_f_at	19.07
Fabp5	160544_at	19.191
Ela1	93783_at	19.195
Serpina3k	92583_at	19.258
Lrg1	97420_at	19.433
Serpina12	160951_at	19.451
Iigp1	96764_at	19.509
Zap70	93661_at	19.579
Cyp7b1	92898_at	20.036
Comt	98535_at	20.112
Iigp1	103963_f_at	20.285
Orm1	100436_at	20.3
Pfkfb3	160641_at	20.322
Tars	95054_at	20.418
Cfh	101853_f_at	20.537
Mup4	101682_f_at	20.748
Reep5	96115_at	20.753

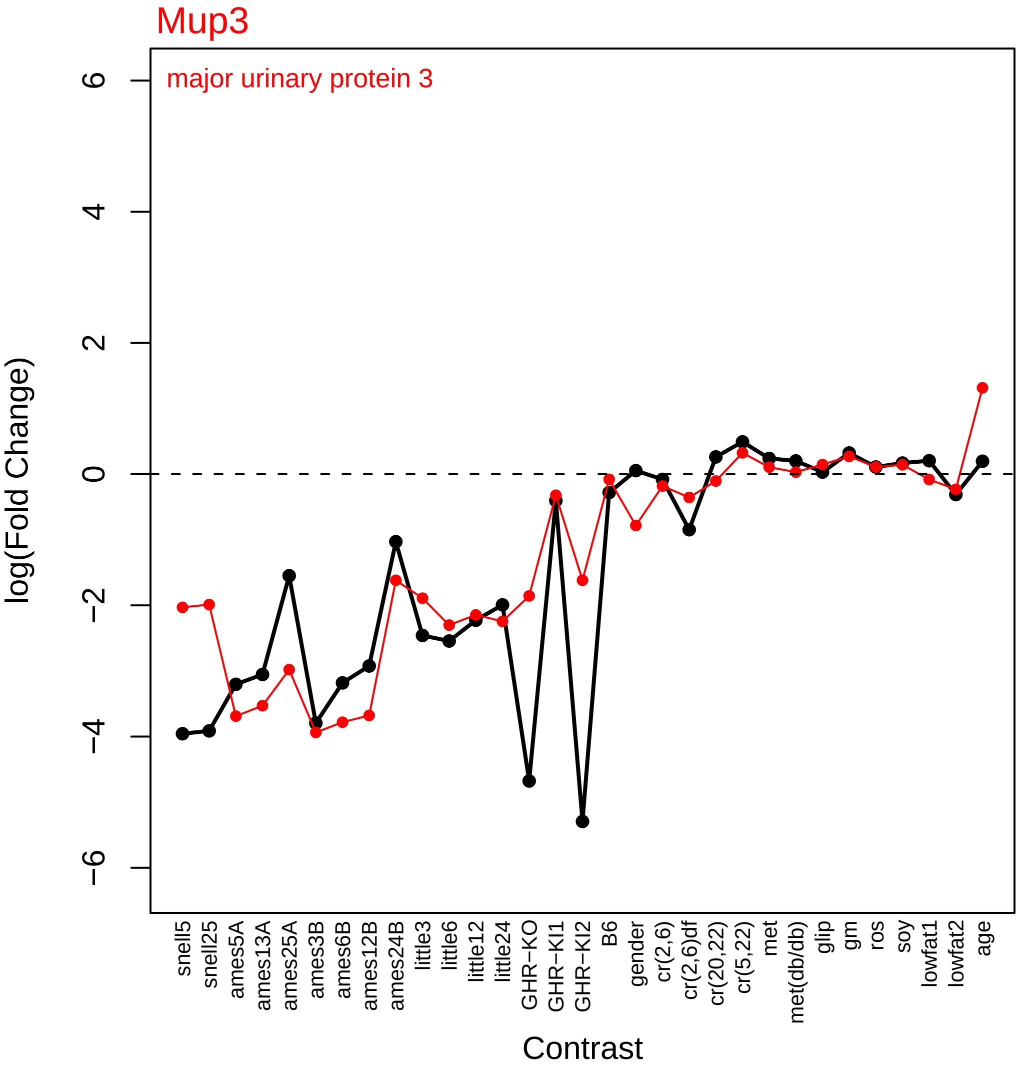
Mup3
major urinary protein 3
log(Fold Change)
6
4
2
0
−2
−4
−6
snell5
snell25
ames5A
ames13A
ames25A
ames3B
ames6B
ames12B
ames24B
little3
little6
little12
little24
GHR−KO
GHR−KI1
GHR−KI2
B6
gender
cr(2,6)
cr(2,6)df
cr(20,22)
cr(5,22)
met
met(db/db)
glip
gm
ros
soy
lowfat1
lowfat2
age
Contrast

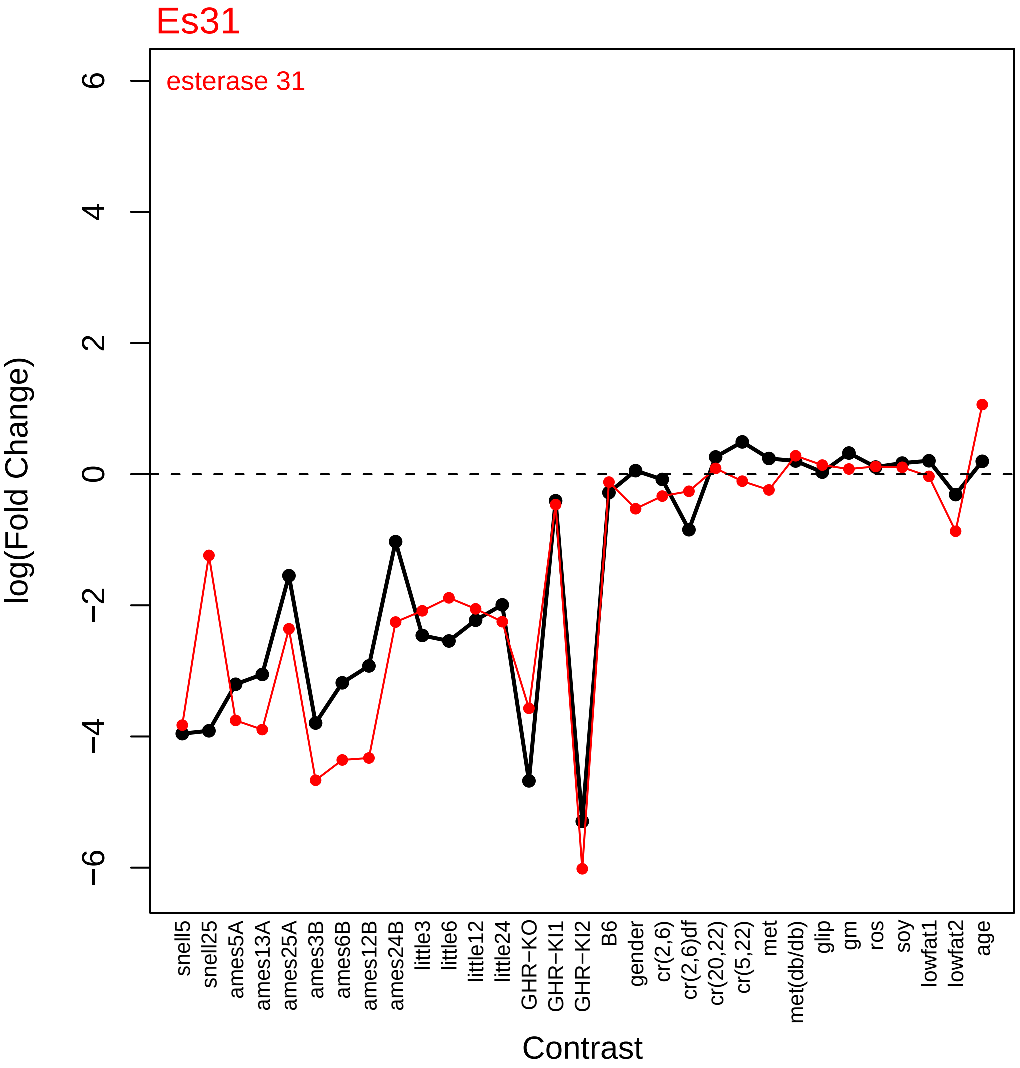

Es31
esterase 31
log(Fold Change)
6
4
2
0
−2
−4
−6
snell5
snell25
ames5A
ames13A
ames25A
ames3B
ames6B
ames12B
ames24B
little3
little6
little12
little24
GHR−KO
GHR−KI1
GHR−KI2
B6
gender
cr(2,6)
cr(2,6)df
cr(20,22)
cr(5,22)
met
met(db/db)
glip
gm
ros
soy
lowfat1
lowfat2
age
Contrast

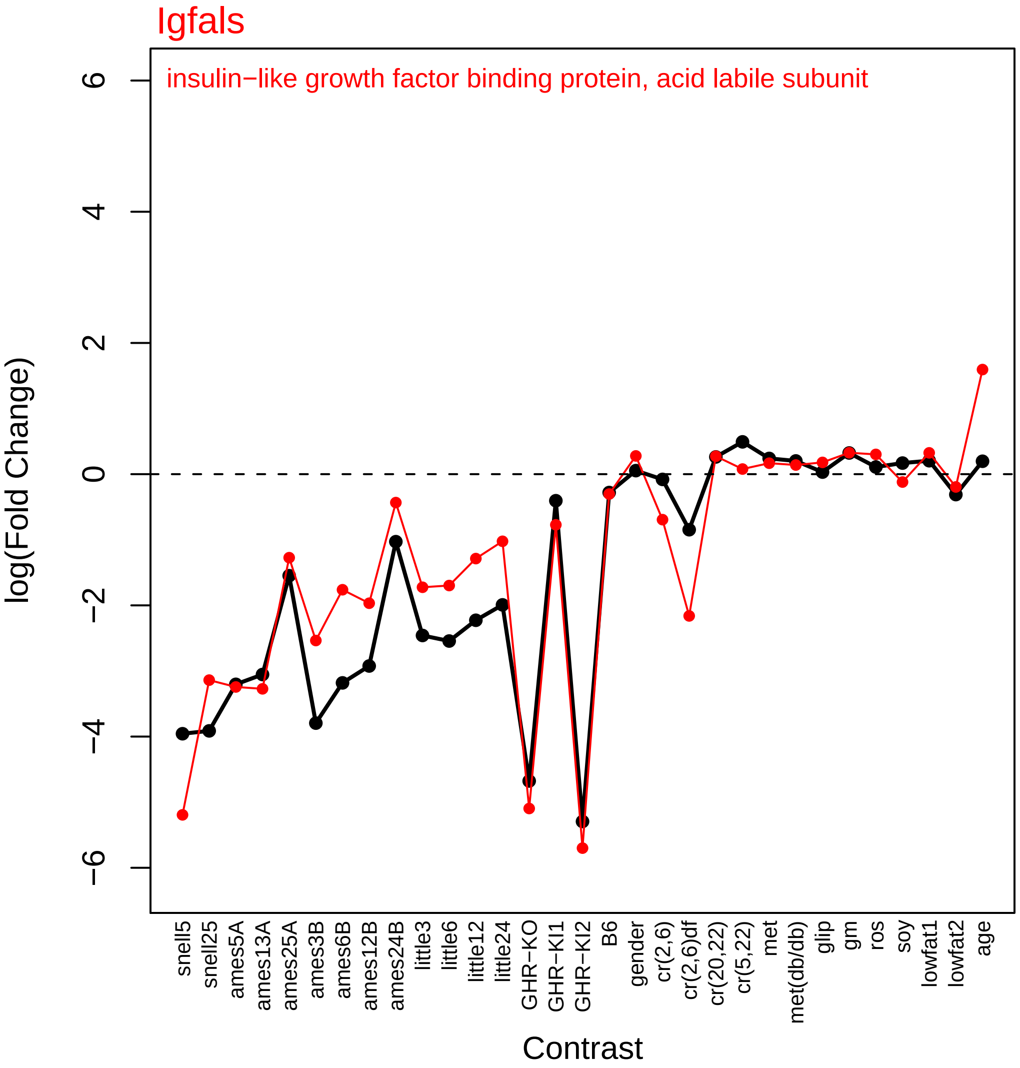

Igfals
insulin−like growth factor binding protein, acid labile subunit
log(Fold Change)
6
4
2
0
−2
−4
−6
snell5
snell25
ames5A
ames13A
ames25A
ames3B
ames6B
ames12B
ames24B
little3
little6
little12
little24
GHR−KO
GHR−KI1
GHR−KI2
B6
gender
cr(2,6)
cr(2,6)df
cr(20,22)
cr(5,22)
met
met(db/db)
glip
gm
ros
soy
lowfat1
lowfat2
age
Contrast

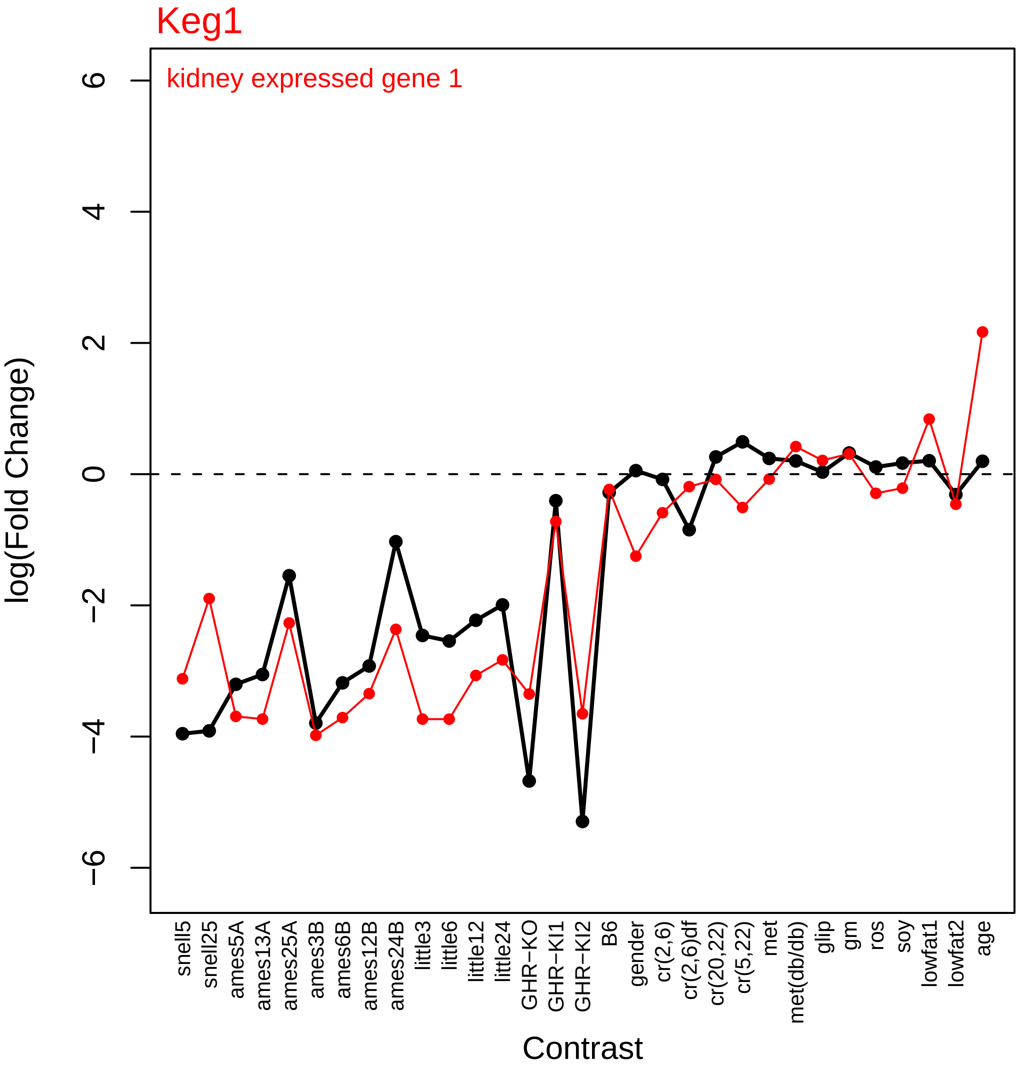

Keg1
kidney expressed gene 1
log(Fold Change)
6
4
2
0
−2
−4
−6
Contrast
snell5
snell25
ames5A
ames13A
ames25A
ames3B
ames6B
ames12B
ames24B
little3
little6
little12
little24
GHR−KO
GHR−KI1
GHR−KI2
B6
gender
cr(2,6)
cr(2,6)df
cr(20,22)
cr(5,22)
met
met(db/db)
glip
gm
ros
soy
lowfat1
lowfat2
age

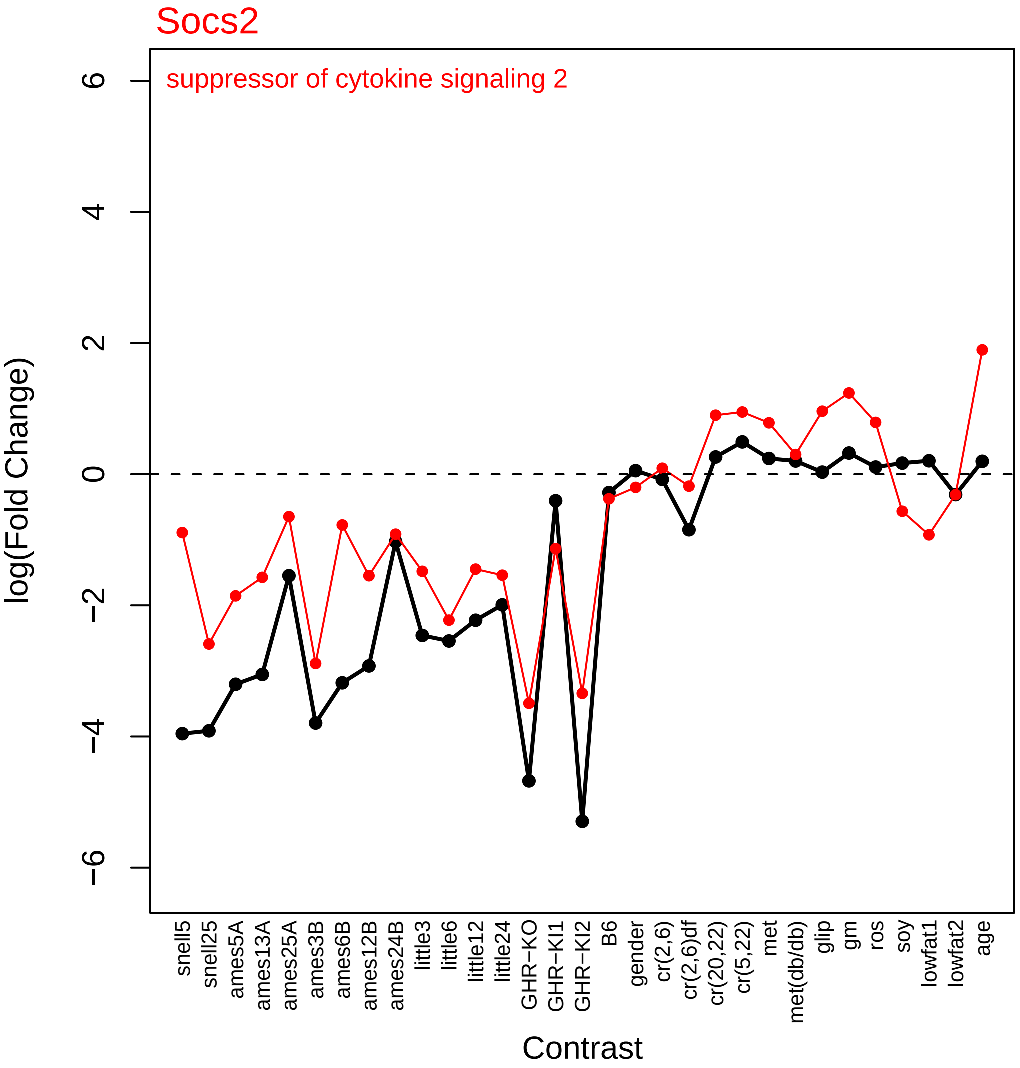
Socs2
suppressor of cytokine signaling 2
log(Fold Change)
Contrast
6
4
2
0
−2
−4
−6
snell5
snell25
ames5A
ames13A
ames25A
ames3B
ames6B
ames12B
ames24B
little3
little6
little12
little24
GHR−KO
GHR−KI1
GHR−KI2
B6
gender
cr(2,6)
cr(2,6)df
cr(20,22)
cr(5,22)
met
met(db/db)
glip
gm
ros
soy
lowfat1
lowfat2
age

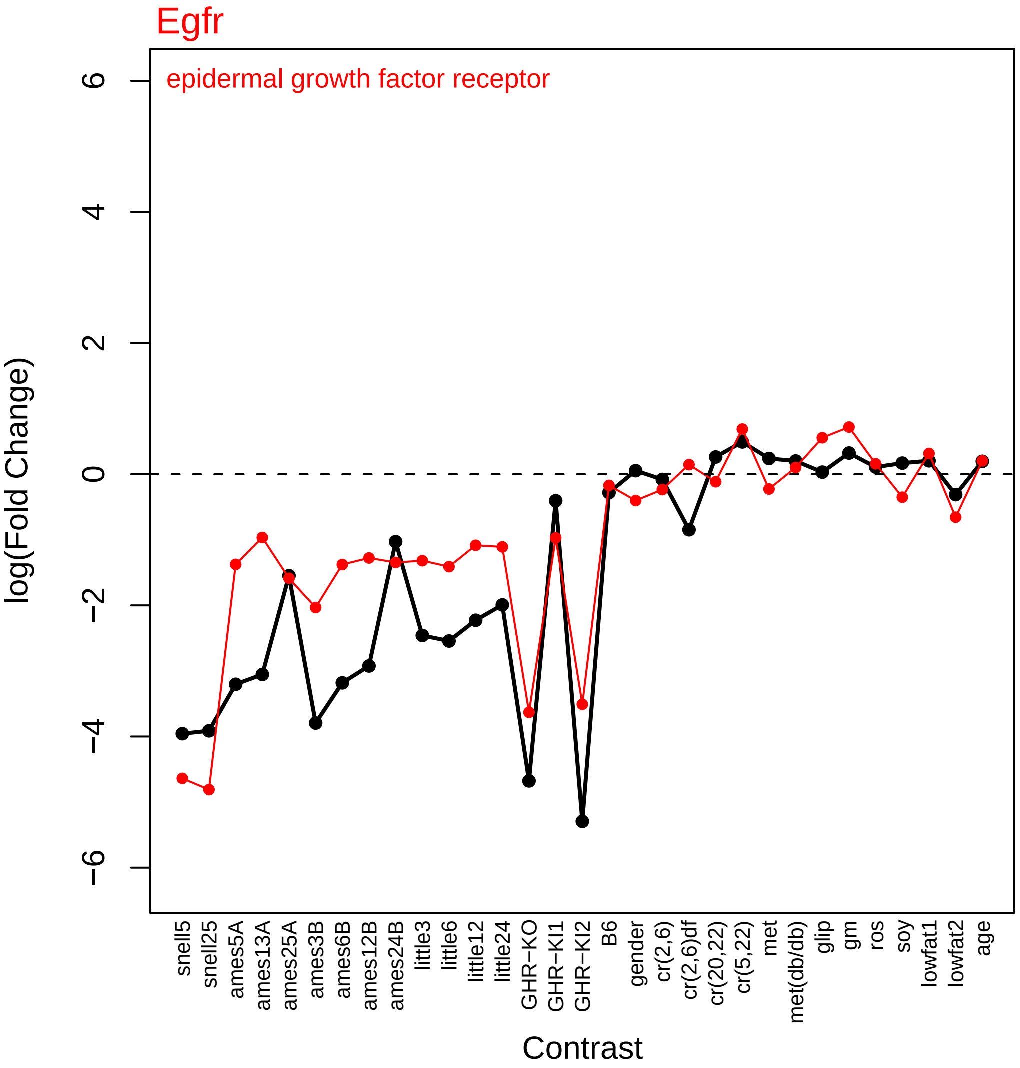

Egfr
epidermal growth factor receptor
log(Fold Change)
6
4
2
0
−2
−4
−6
snell5
snell25
ames5A
ames13A
ames25A
ames3B
ames6B
ames12B
ames24B
little3
little6
little12
little24
GHR−KO
GHR−KI1
GHR−KI2
B6
gender
cr(2,6)
cr(2,6)df
cr(20,22)
cr(5,22)
met
met(db/db)
glip
gm
ros
soy
lowfat1
lowfat2
age
Contrast

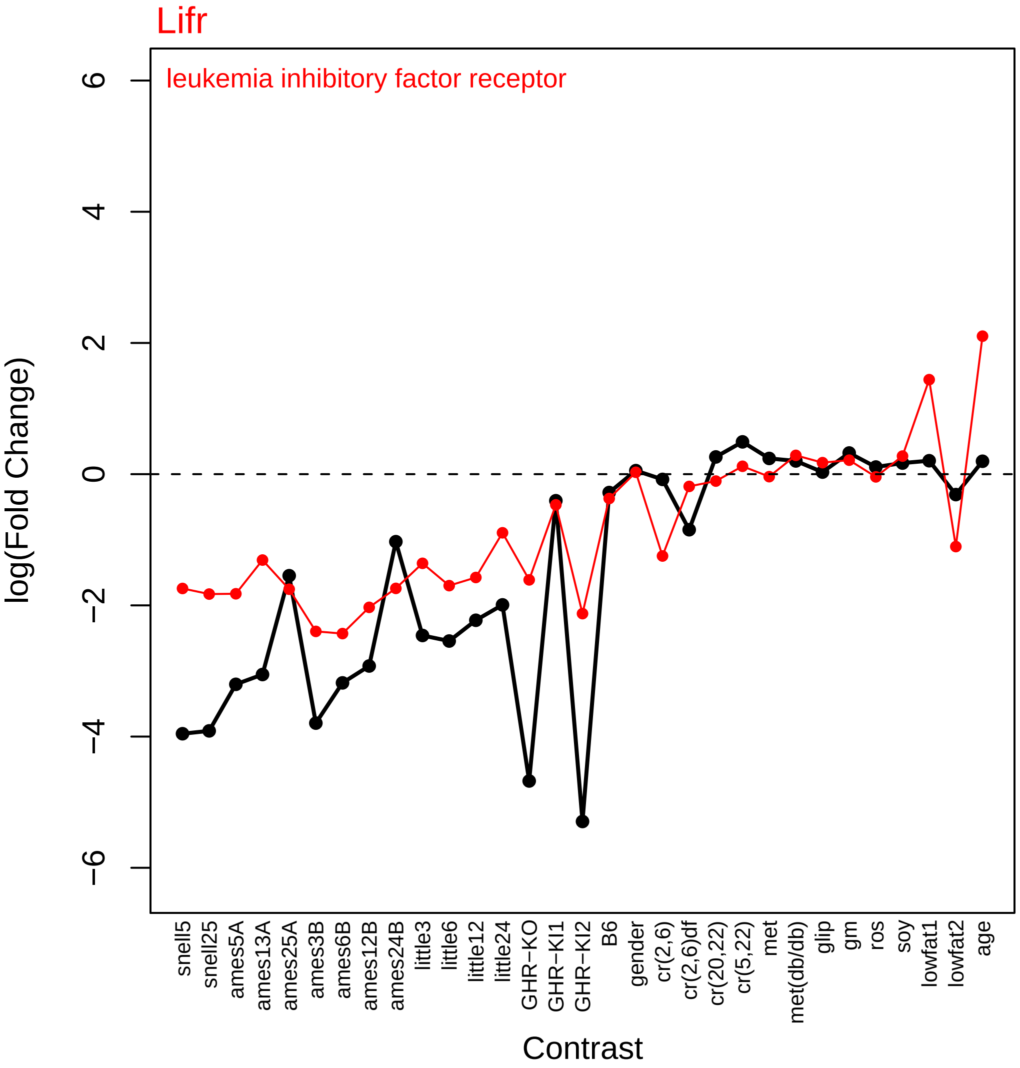

Lifr
leukemia inhibitory factor receptor
log(Fold Change)
6
4
2
0
−2
−4
−6
snell5
snell25
ames5A
ames13A
ames25A
ames3B
ames6B
ames12B
ames24B
little3
little6
little12
little24
GHR−KO
GHR−KI1
GHR−KI2
B6
gender
cr(2,6)
cr(2,6)df
cr(20,22)
cr(5,22)
met
met(db/db)
glip
gm
ros
soy
lowfat1
lowfat2
age
Contrast

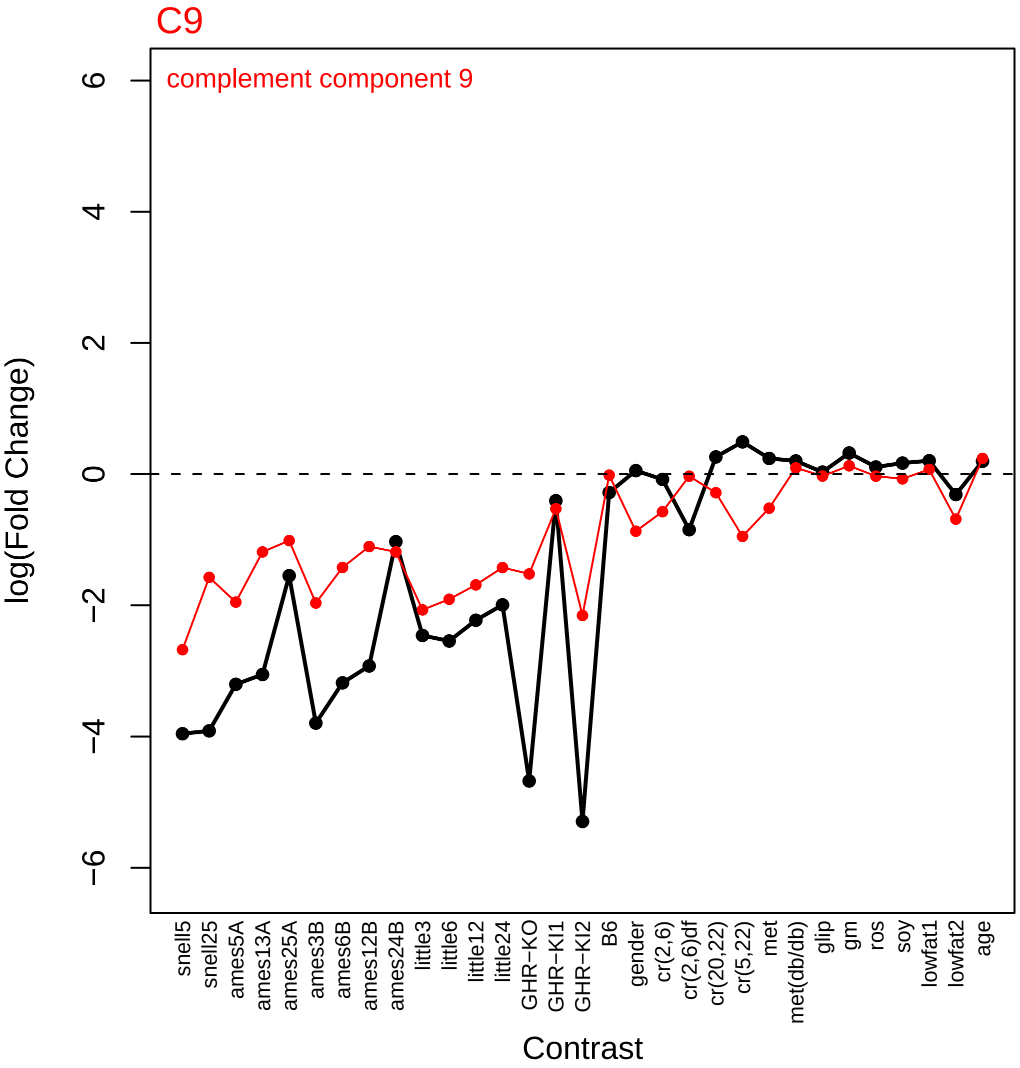
C9
complement component 9
log(Fold Change)
6
4
2
0
−2
−4
−6
snell5
snell25
ames5A
ames13A
ames25A
ames3B
ames6B
ames12B
ames24B
little3
little6
little12
little24
GHR−KO
GHR−KI1
GHR−KI2
B6
gender
cr(2,6)
cr(2,6)df
cr(20,22)
cr(5,22)
met
met(db/db)
glip
gm
ros
soy
lowfat1
lowfat2
age
Contrast

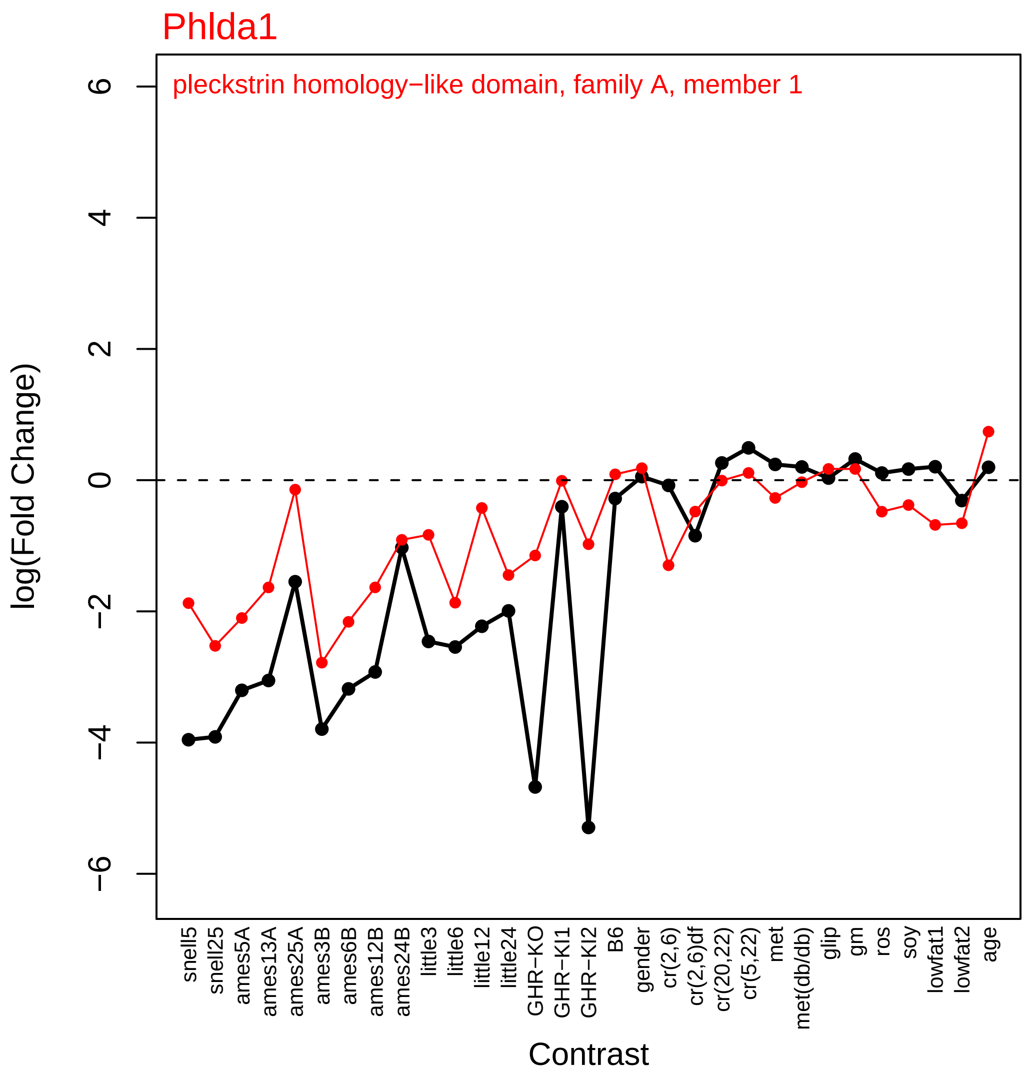
Phlda1
pleckstrin homology−like domain, family A, member 1
log(Fold Change)
6
4
2
0
−2
−4
−6
Contrast
snell5
snell25
ames5A
ames13A
ames25A
ames3B
ames6B
ames12B
ames24B
little3
little6
little12
little24
GHR−KO
GHR−KI1
GHR−KI2
B6
gender
cr(2,6)
cr(2,6)df
cr(20,22)
cr(5,22)
met
met(db/db)
glip
gm
ros
soy
lowfat1
lowfat2
age

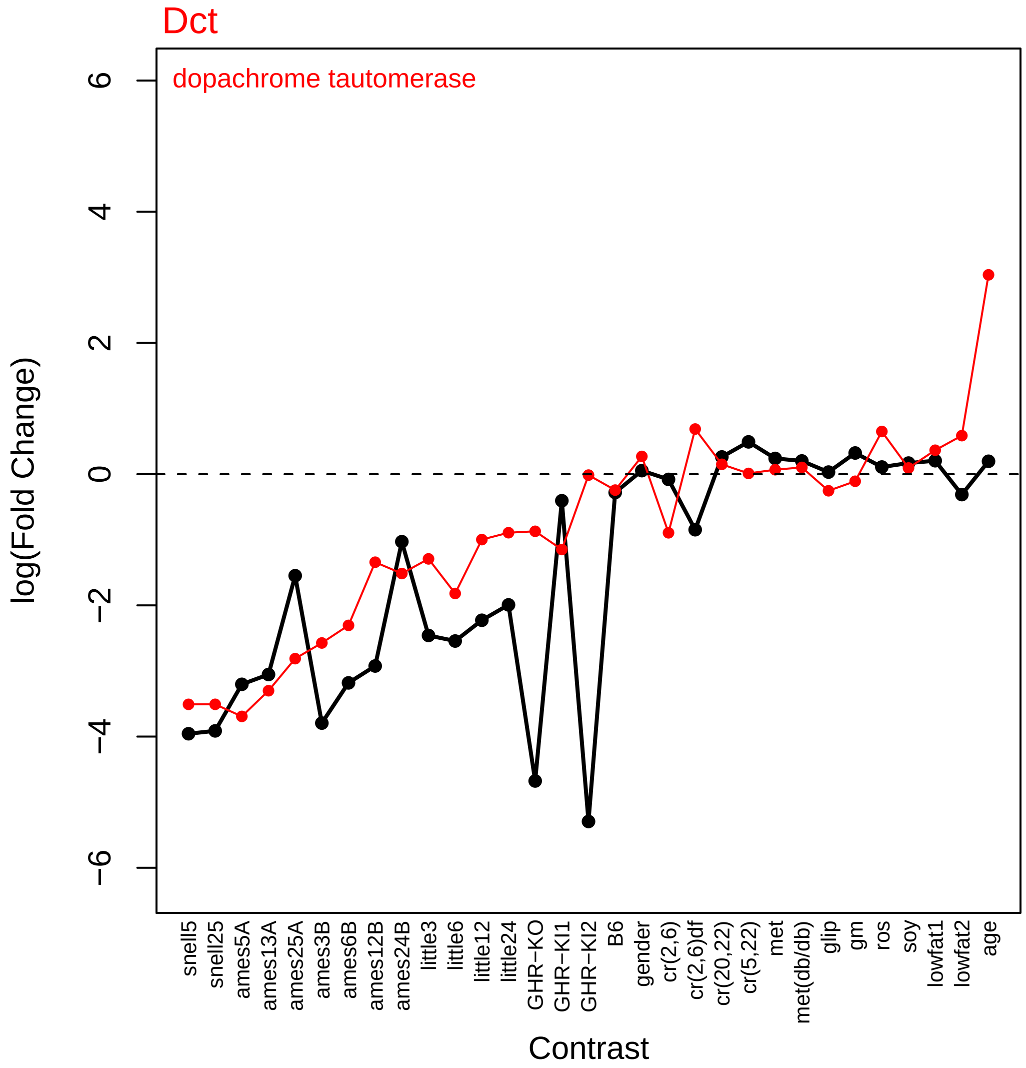
Dct
dopachrome tautomerase
log(Fold Change)
6
4
2
0
−2
−4
−6
snell5
snell25
ames5A
ames13A
ames25A
ames3B
ames6B
ames12B
ames24B
little3
little6
little12
little24
GHR−KO
GHR−KI1
GHR−KI2
B6
gender
cr(2,6)
cr(2,6)df
cr(20,22)
cr(5,22)
met
met(db/db)
glip
gm
ros
soy
lowfat1
lowfat2
age
Contrast

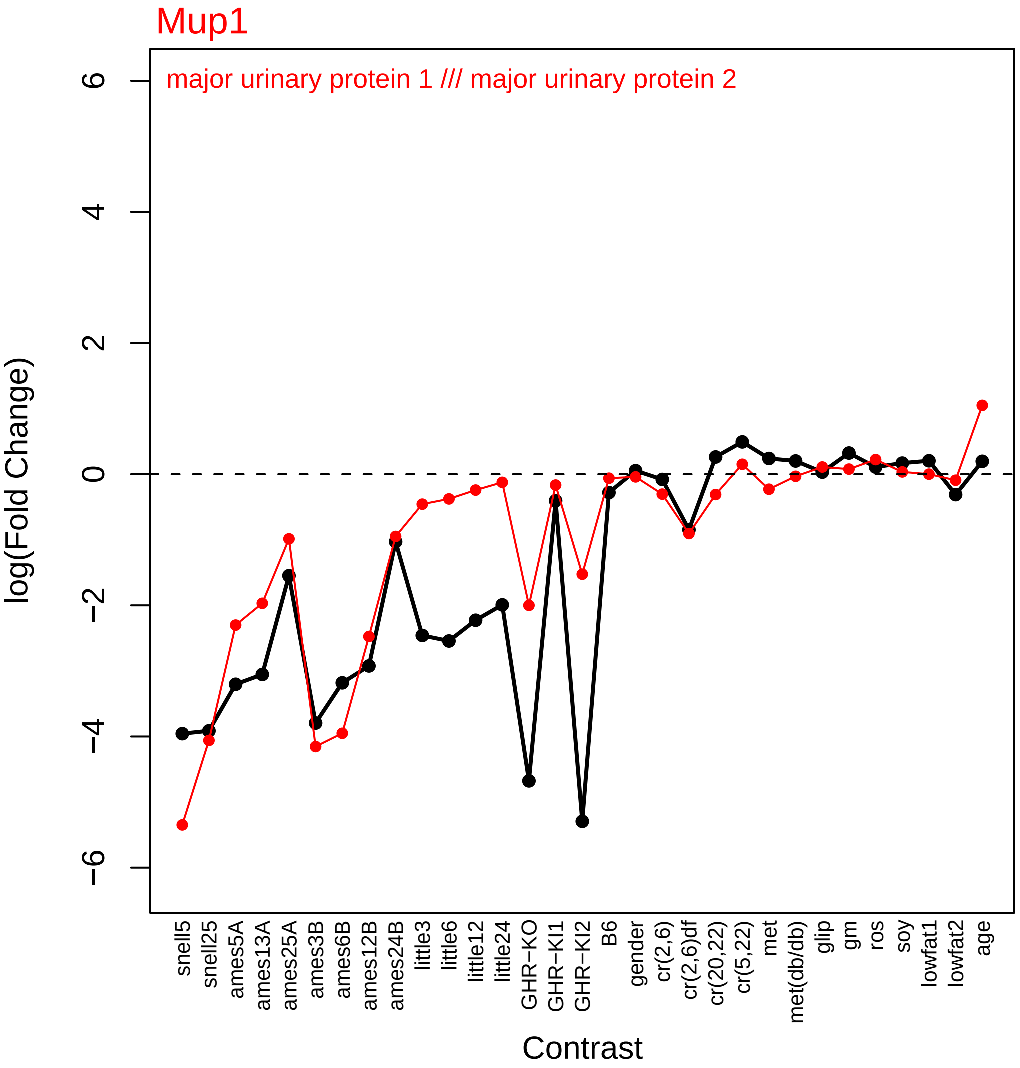
Mup1
major urinary protein 1 /// major urinary protein 2
log(Fold Change)
6
4
2
0
−2
−4
−6
snell5
snell25
ames5A
ames13A
ames25A
ames3B
ames6B
ames12B
ames24B
little3
little6
little12
little24
GHR−KO
GHR−KI1
GHR−KI2
B6
gender
cr(2,6)
cr(2,6)df
cr(20,22)
cr(5,22)
met
met(db/db)
glip
gm
ros
soy
lowfat1
lowfat2
age
Contrast

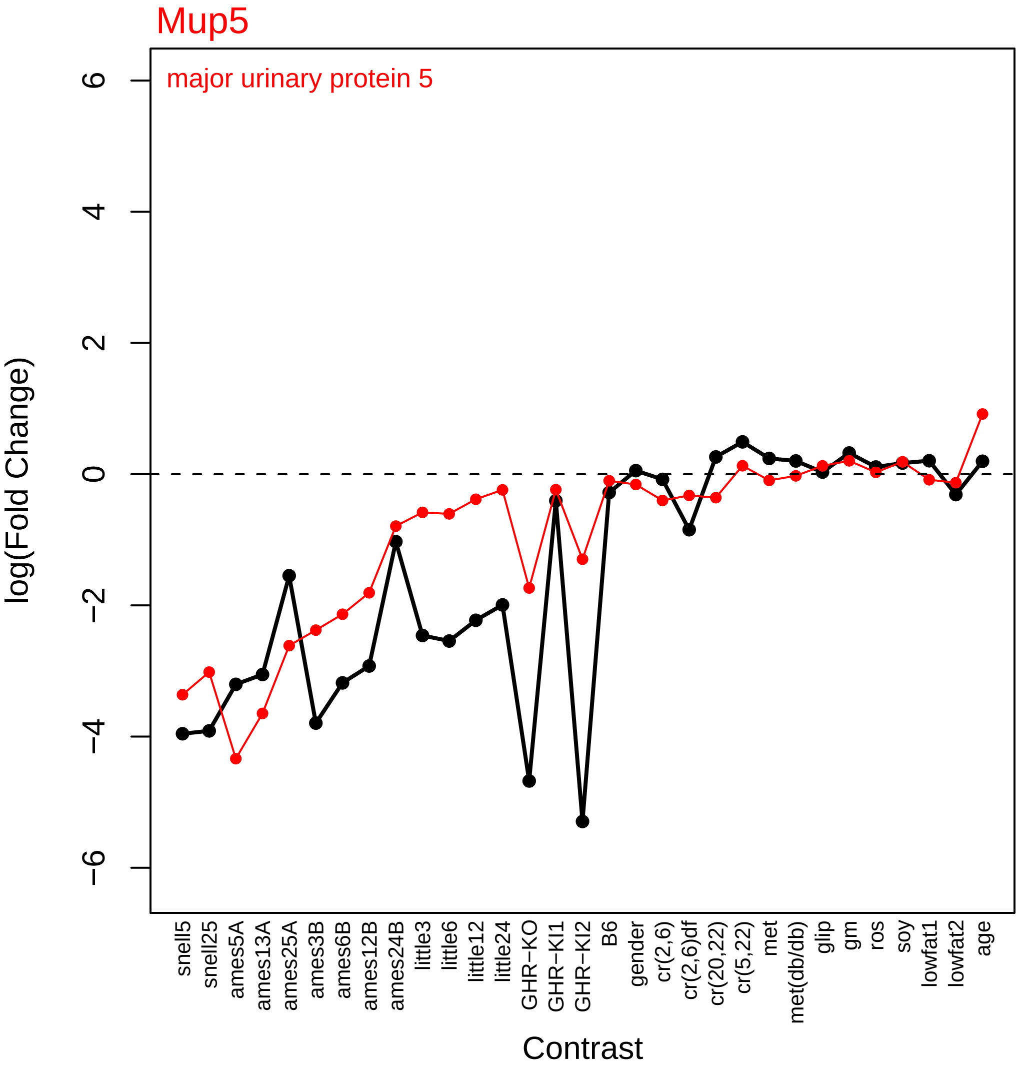
Mup5
major urinary protein 5
log(Fold Change)
6
4
2
0
−2
−4
−6
snell5
snell25
ames5A
ames13A
ames25A
ames3B
ames6B
ames12B
ames24B
little3
little6
little12
little24
GHR−KO
GHR−KI1
GHR−KI2
B6
gender
cr(2,6)
cr(2,6)df
cr(20,22)
cr(5,22)
met
met(db/db)
glip
gm
ros
soy
lowfat1
lowfat2
age
Contrast

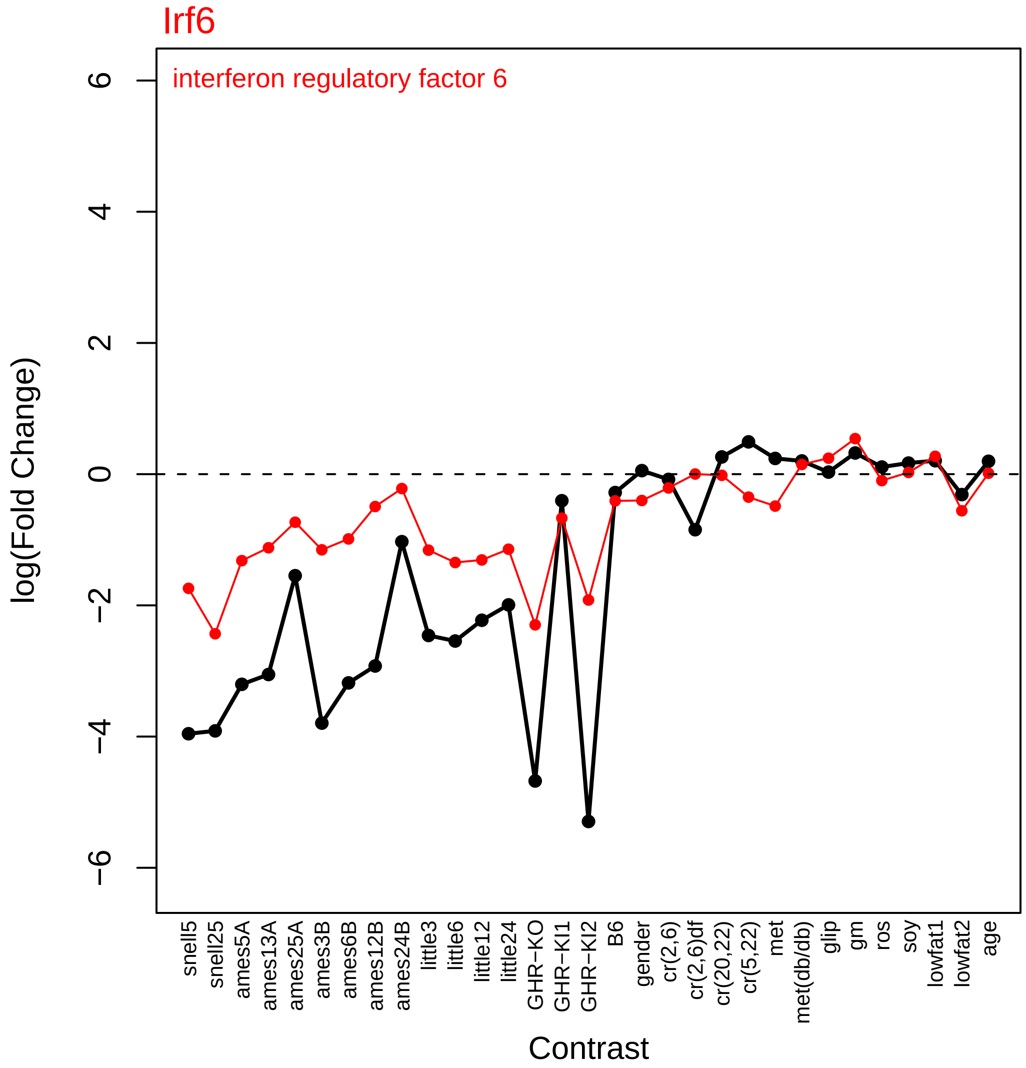

Irf6
interferon regulatory factor 6
log(Fold Change)
Contrast
6
4
2
0
−2
−4
−6
snell5
snell25
ames5A
ames13A
ames25A
ames3B
ames6B
ames12B
ames24B
little3
little6
little12
little24
GHR−KO
GHR−KI1
GHR−KI2
B6
gender
cr(2,6)
cr(2,6)df
cr(20,22)
cr(5,22)
met
met(db/db)
glip
gm
ros
soy
lowfat1
lowfat2
age

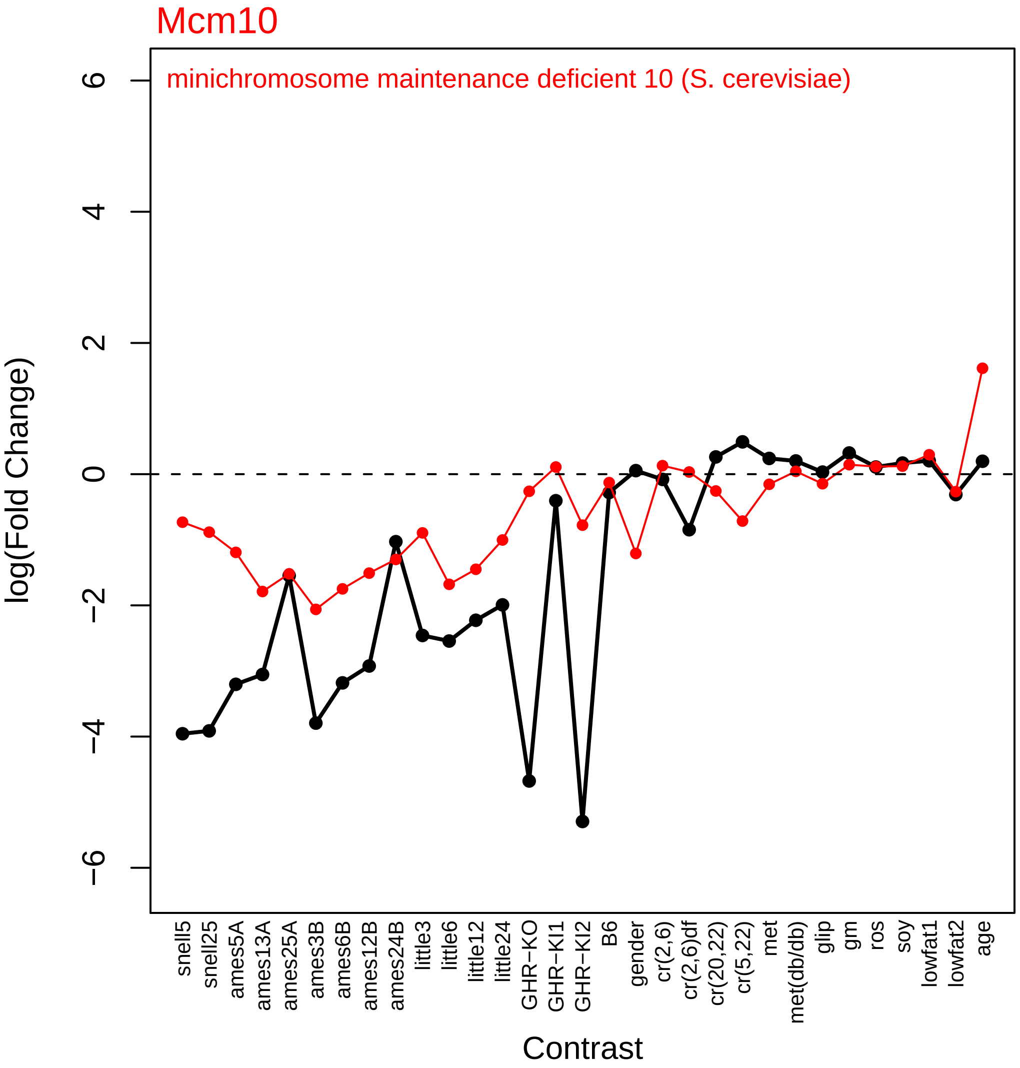

Mcm10
minichromosome maintenance deficient 10 (S. cerevisiae)
log(Fold Change)
6
4
2
0
−2
−4
−6
snell5
snell25
ames5A
ames13A
ames25A
ames3B
ames6B
ames12B
ames24B
little3
little6
little12
little24
GHR−KO
GHR−KI1
GHR−KI2
B6
gender
cr(2,6)
cr(2,6)df
cr(20,22)
cr(5,22)
met
met(db/db)
glip
gm
ros
soy
lowfat1
lowfat2
age
Contrast

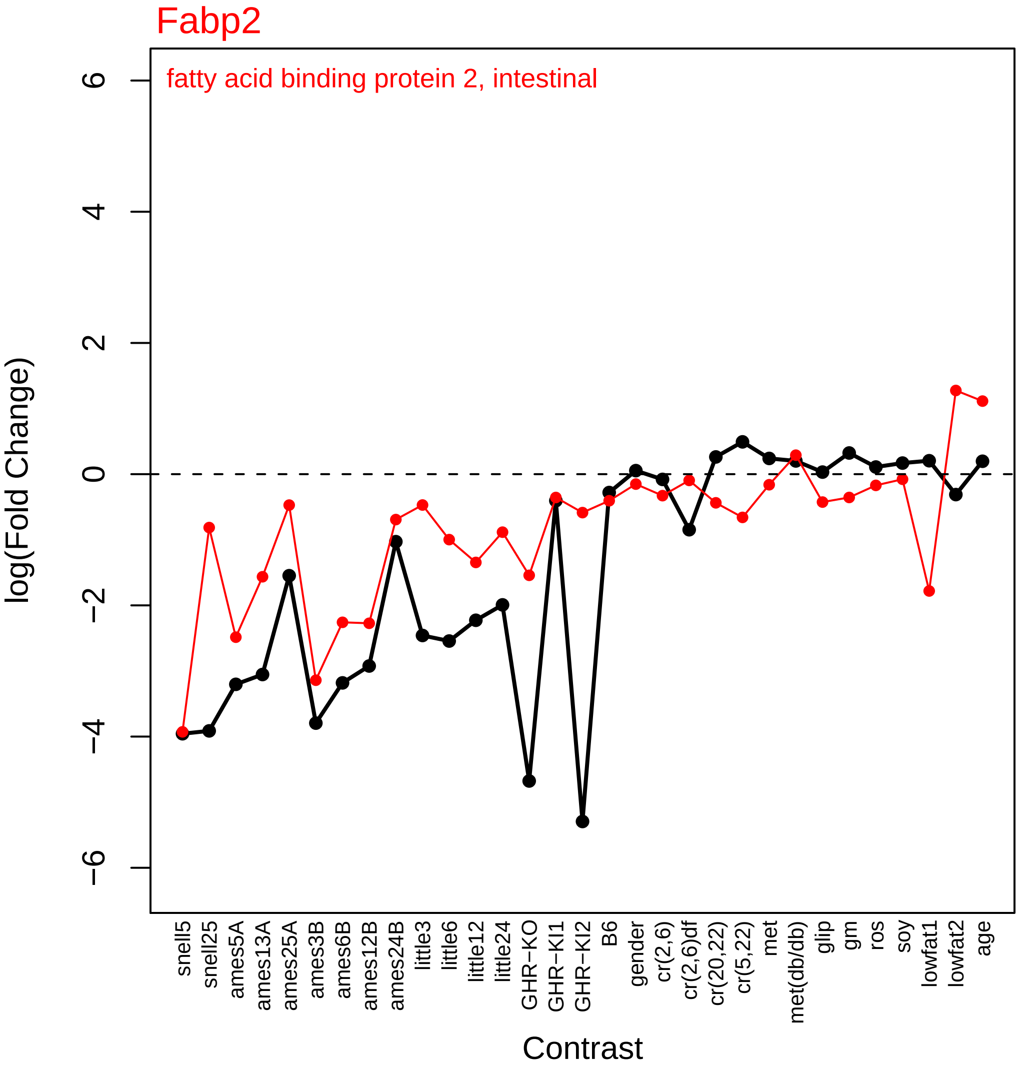

Fabp2
fatty acid binding protein 2, intestinal
log(Fold Change)
6
4
2
0
−2
−4
−6
Contrast
snell5
snell25
ames5A
ames13A
ames25A
ames3B
ames6B
ames12B
ames24B
little3
little6
little12
little24
GHR−KO
GHR−KI1
GHR−KI2
B6
gender
cr(2,6)
cr(2,6)df
cr(20,22)
cr(5,22)
met
met(db/db)
glip
gm
ros
soy
lowfat1
lowfat2
age

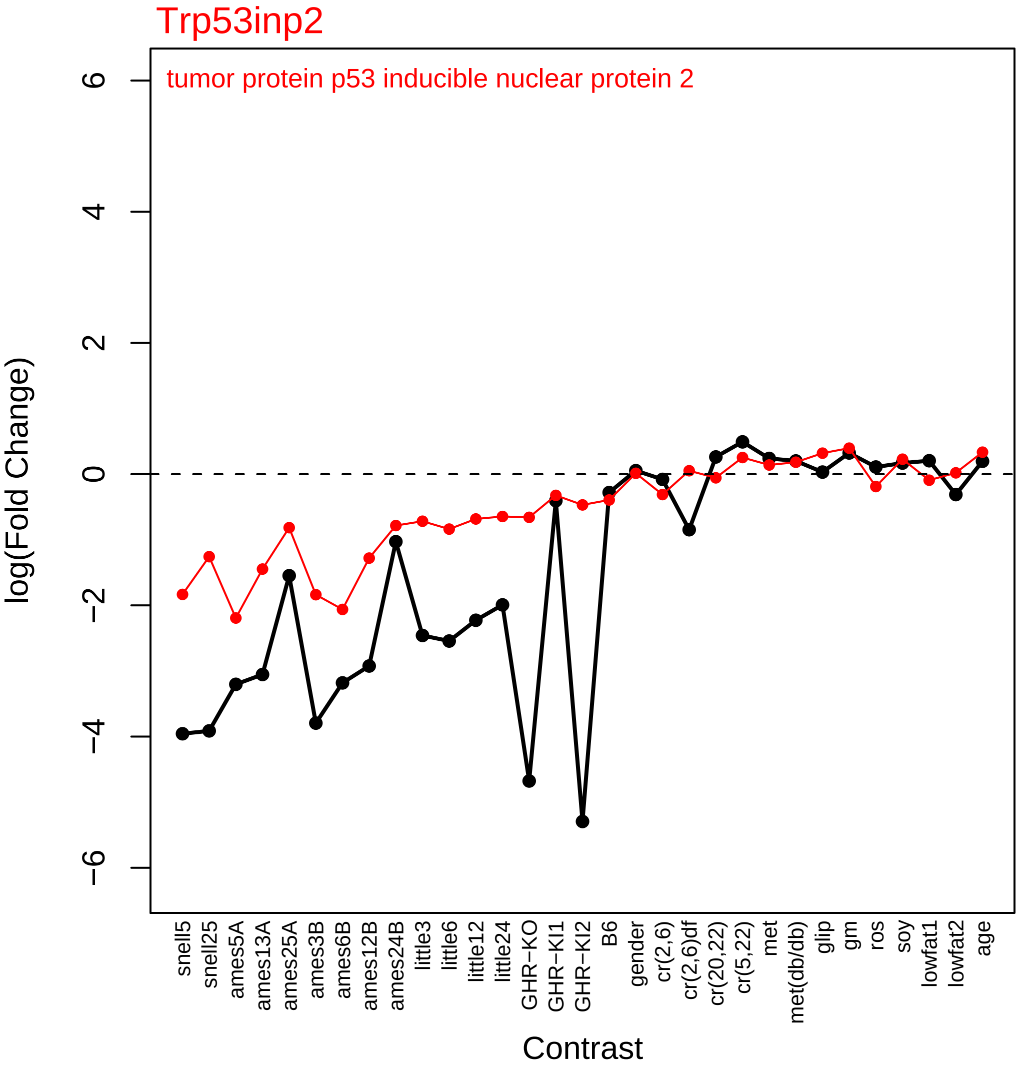
Trp53inp2
tumor protein p53 inducible nuclear protein 2
log(Fold Change)
Contrast
6
4
2
0
−2
−4
−6
snell5
snell25
ames5A
ames13A
ames25A
ames3B
ames6B
ames12B
ames24B
little3
little6
little12
little24
GHR−KO
GHR−KI1
GHR−KI2
B6
gender
cr(2,6)
cr(2,6)df
cr(20,22)
cr(5,22)
met
met(db/db)
glip
gm
ros
soy
lowfat1
lowfat2
age

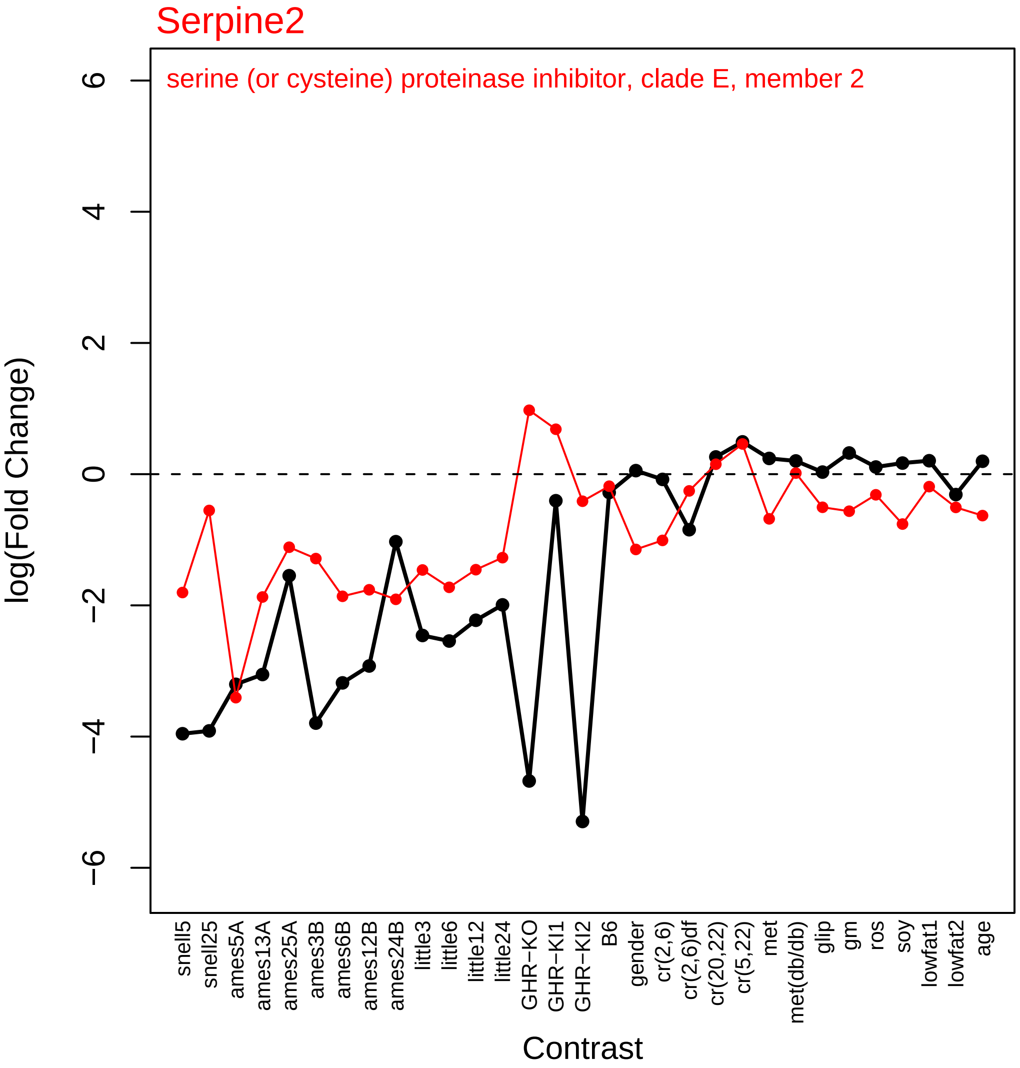

Serpine2
serine (or cysteine) proteinase inhibitor, clade E, member 2
log(Fold Change)
6
4
2
0
−2
−4
−6
snell5
snell25
ames5A
ames13A
ames25A
ames3B
ames6B
ames12B
ames24B
little3
little6
little12
little24
GHR−KO
GHR−KI1
GHR−KI2
B6
gender
cr(2,6)
cr(2,6)df
cr(20,22)
cr(5,22)
met
met(db/db)
glip
gm
ros
soy
lowfat1
lowfat2
age
Contrast

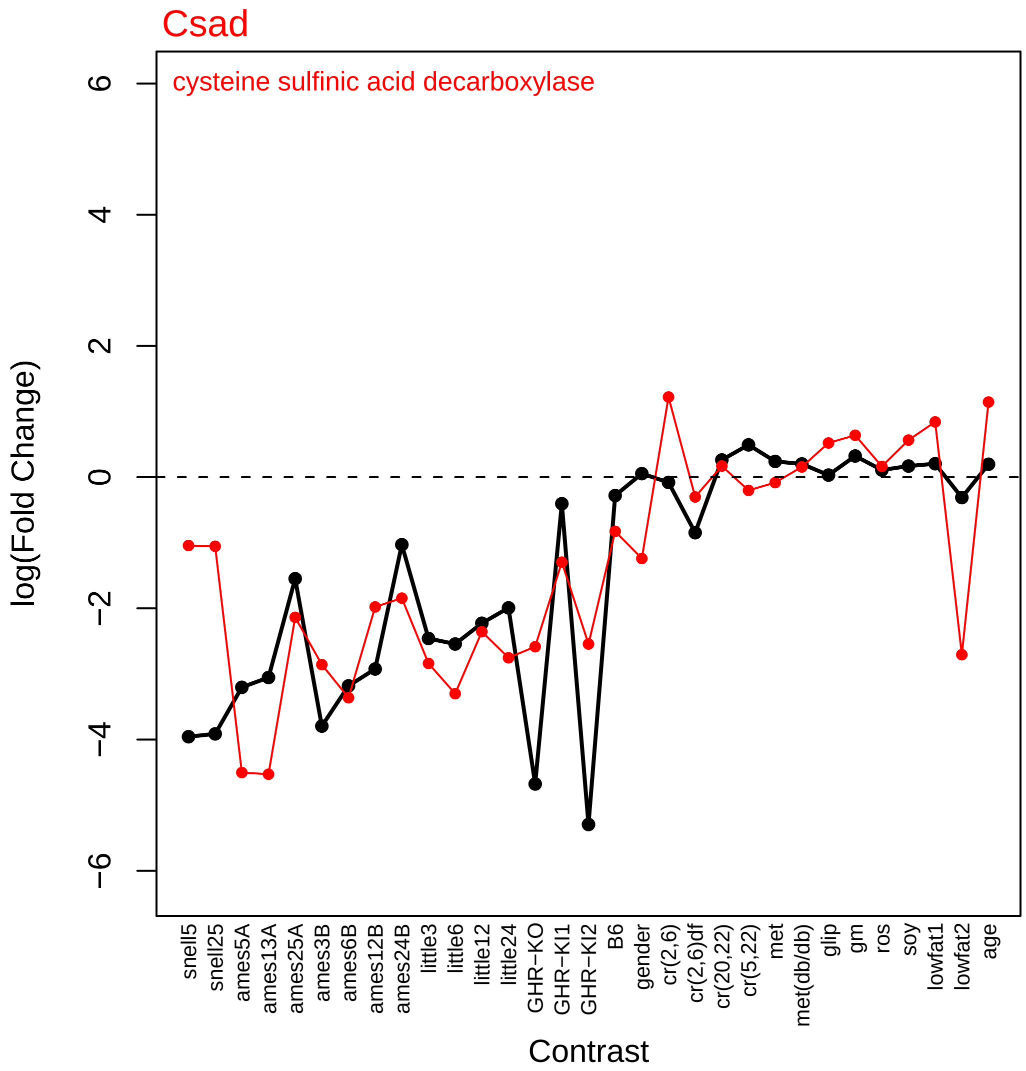

Csad
cysteine sulfinic acid decarboxylase
log(Fold Change)
6
4
2
0
−2
−4
−6
snell5
snell25
ames5A
ames13A
ames25A
ames3B
ames6B
ames12B
ames24B
little3
little6
little12
little24
GHR−KO
GHR−KI1
GHR−KI2
B6
gender
cr(2,6)
cr(2,6)df
cr(20,22)
cr(5,22)
met
met(db/db)
glip
gm
ros
soy
lowfat1
lowfat2
age
Contrast

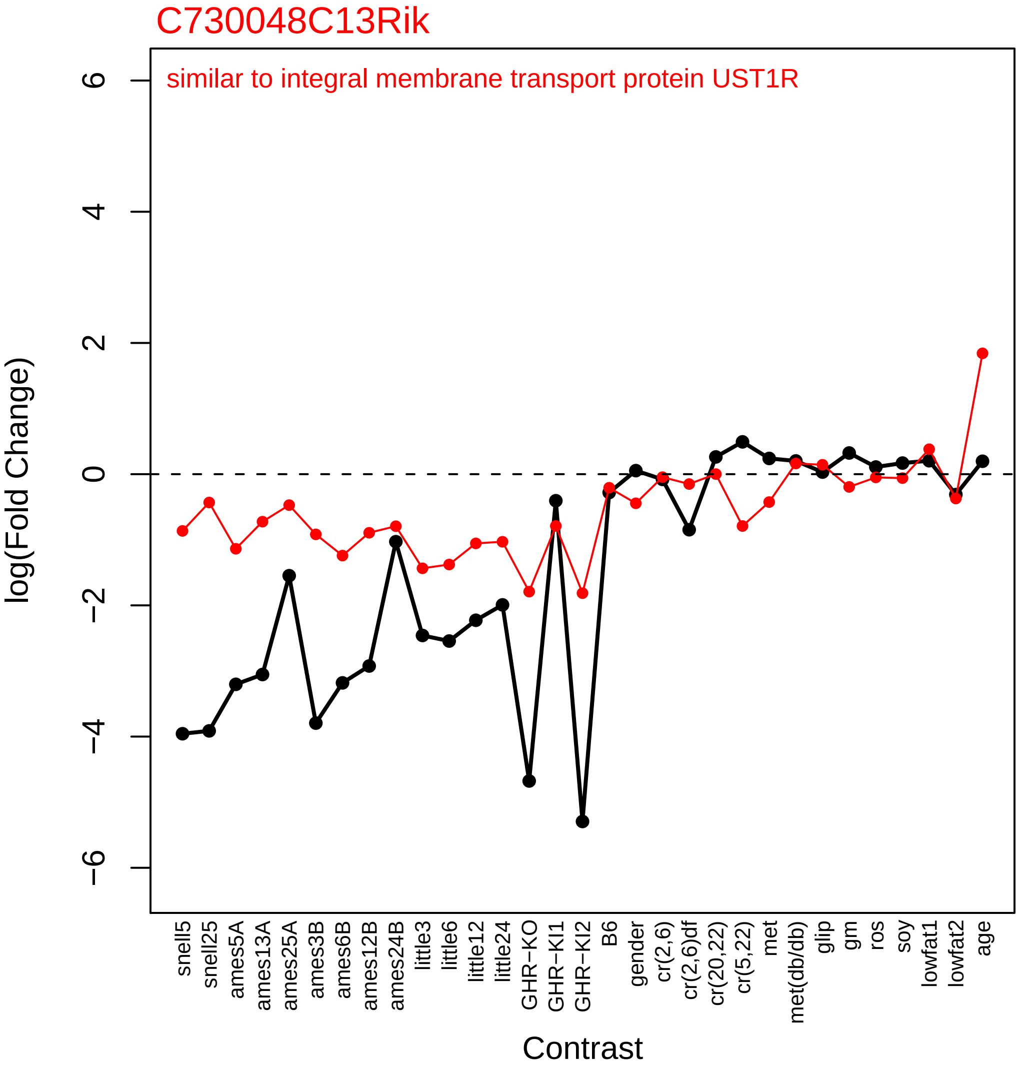

C730048C13Rik
similar to integral membrane transport protein UST1R
log(Fold Change)
Contrast
6
4
2
0
−2
−4
−6
snell5
snell25
ames5A
ames13A
ames25A
ames3B
ames6B
ames12B
ames24B
little3
little6
little12
little24
GHR−KO
GHR−KI1
GHR−KI2
B6
gender
cr(2,6)
cr(2,6)df
cr(20,22)
cr(5,22)
met
met(db/db)
glip
gm
ros
soy
lowfat1
lowfat2
age

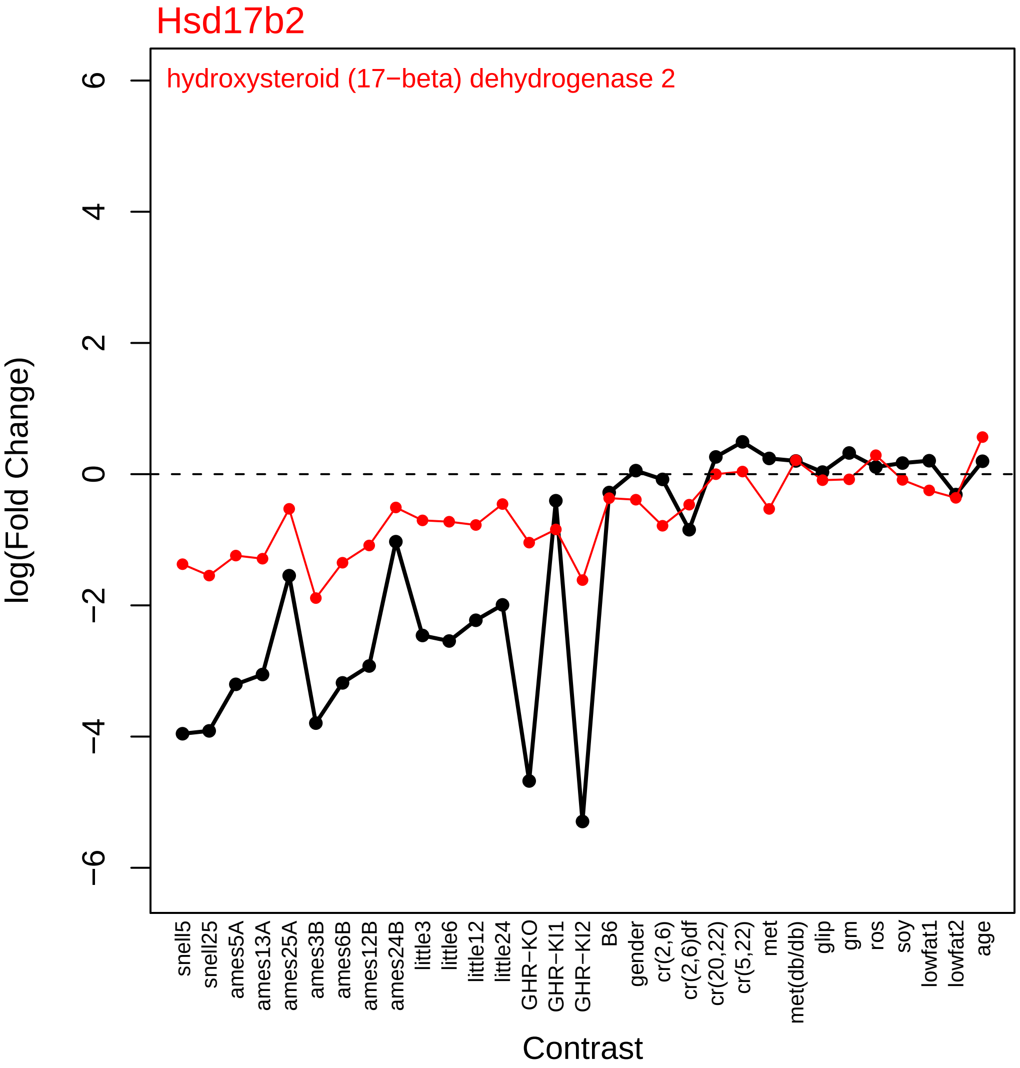
Hsd17b2
hydroxysteroid (17−beta) dehydrogenase 2
log(Fold Change)
6
4
2
0
−2
−4
−6
snell5
snell25
ames5A
ames13A
ames25A
ames3B
ames6B
ames12B
ames24B
little3
little6
little12
little24
GHR−KO
GHR−KI1
GHR−KI2
B6
gender
cr(2,6)
cr(2,6)df
cr(20,22)
cr(5,22)
met
met(db/db)
glip
gm
ros
soy
lowfat1
lowfat2
age
Contrast

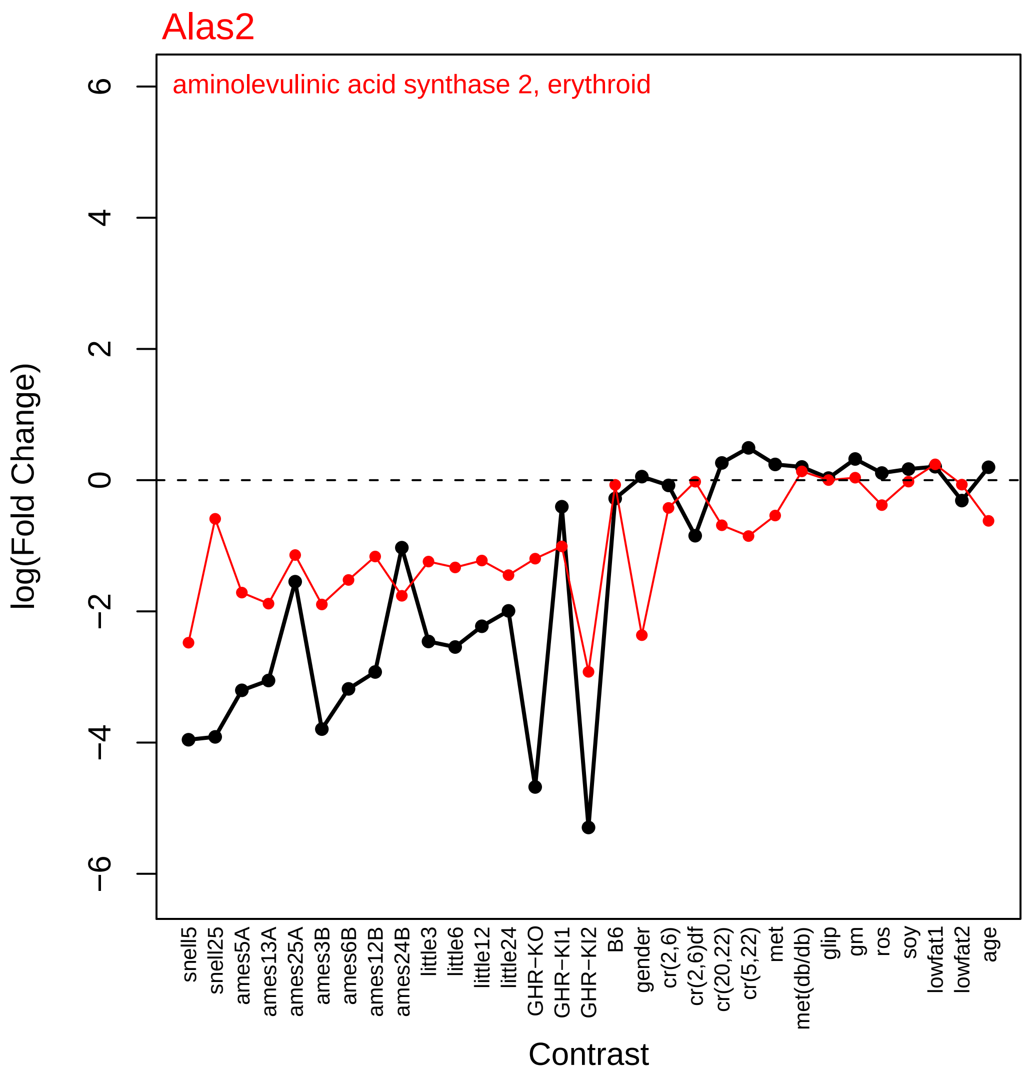
Alas2
aminolevulinic acid synthase 2, erythroid
log(Fold Change)
6
4
2
0
−2
−4
−6
Contrast
snell5
snell25
ames5A
ames13A
ames25A
ames3B
ames6B
ames12B
ames24B
little3
little6
little12
little24
GHR−KO
GHR−KI1
GHR−KI2
B6
gender
cr(2,6)
cr(2,6)df
cr(20,22)
cr(5,22)
met
met(db/db)
glip
gm
ros
soy
lowfat1
lowfat2
age

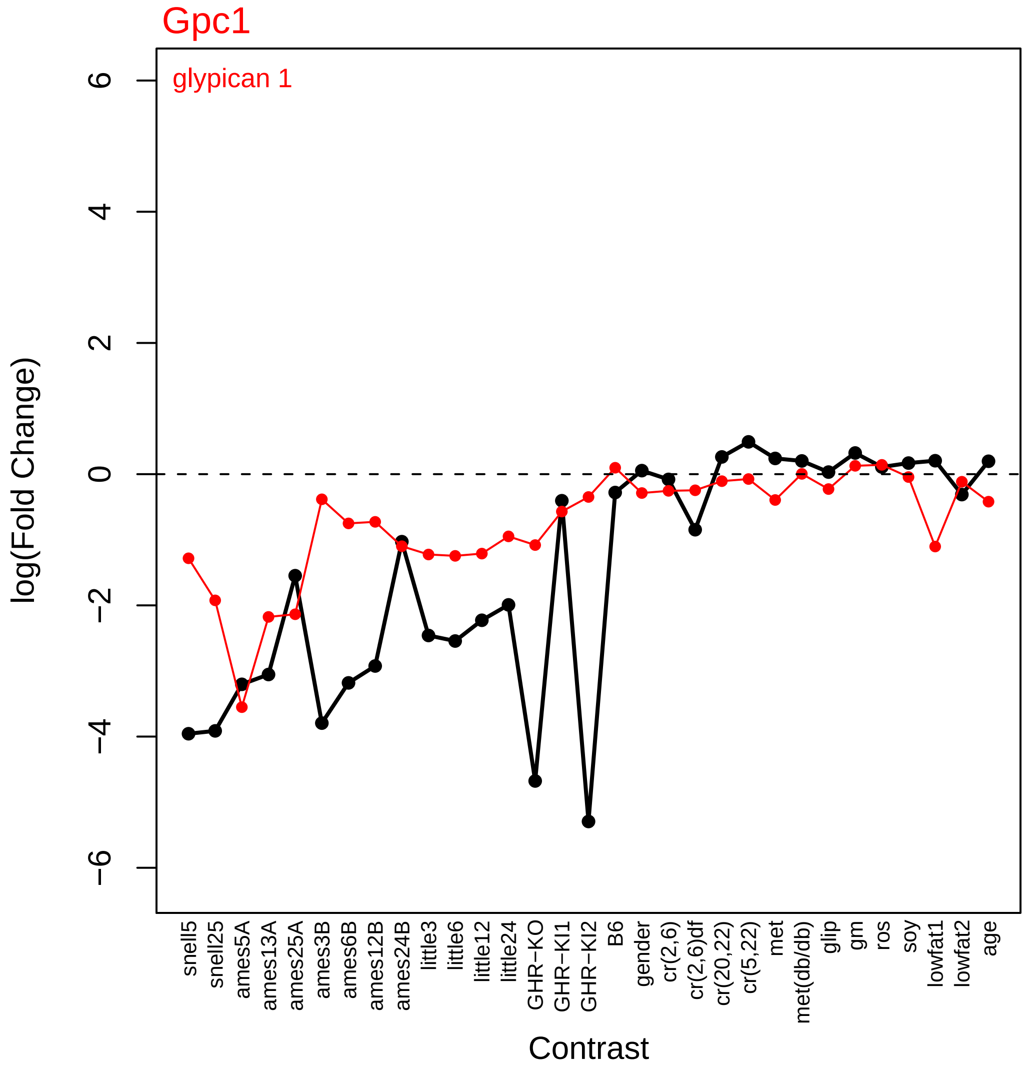

Gpc1
glypican 1
log(Fold Change)
6
4
2
0
−2
−4
−6
snell5
snell25
ames5A
ames13A
ames25A
ames3B
ames6B
ames12B
ames24B
little3
little6
little12
little24
GHR−KO
GHR−KI1
GHR−KI2
B6
gender
cr(2,6)
cr(2,6)df
cr(20,22)
cr(5,22)
met
met(db/db)
glip
gm
ros
soy
lowfat1
lowfat2
age
Contrast

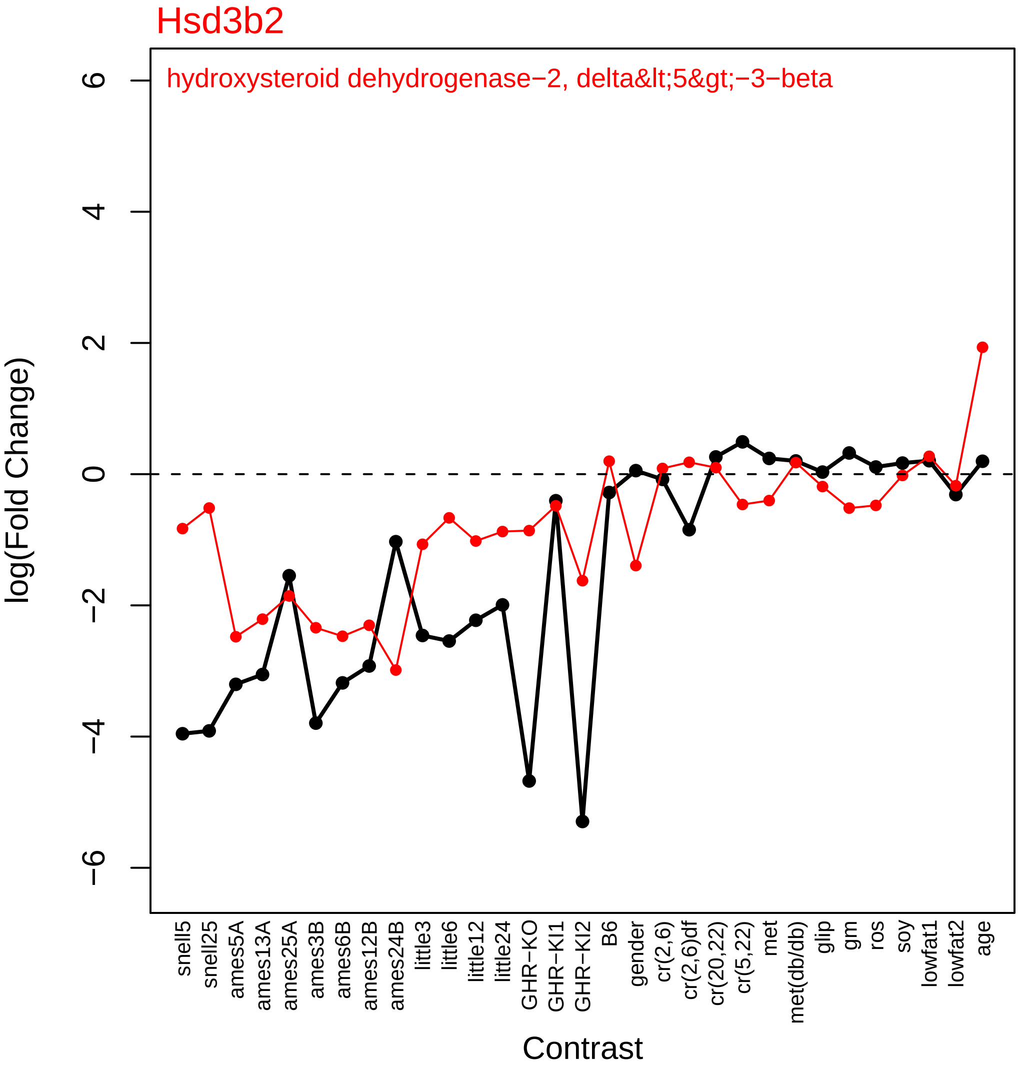
Hsd3b2
hydroxysteroid dehydrogenase−2, delta<5>−3−beta
log(Fold Change)
6
4
2
0
−2
−4
−6
Contrast
snell5
snell25
ames5A
ames13A
ames25A
ames3B
ames6B
ames12B
ames24B
little3
little6
little12
little24
GHR−KO
GHR−KI1
GHR−KI2
B6
gender
cr(2,6)
cr(2,6)df
cr(20,22)
cr(5,22)
met
met(db/db)
glip
gm
ros
soy
lowfat1
lowfat2
age

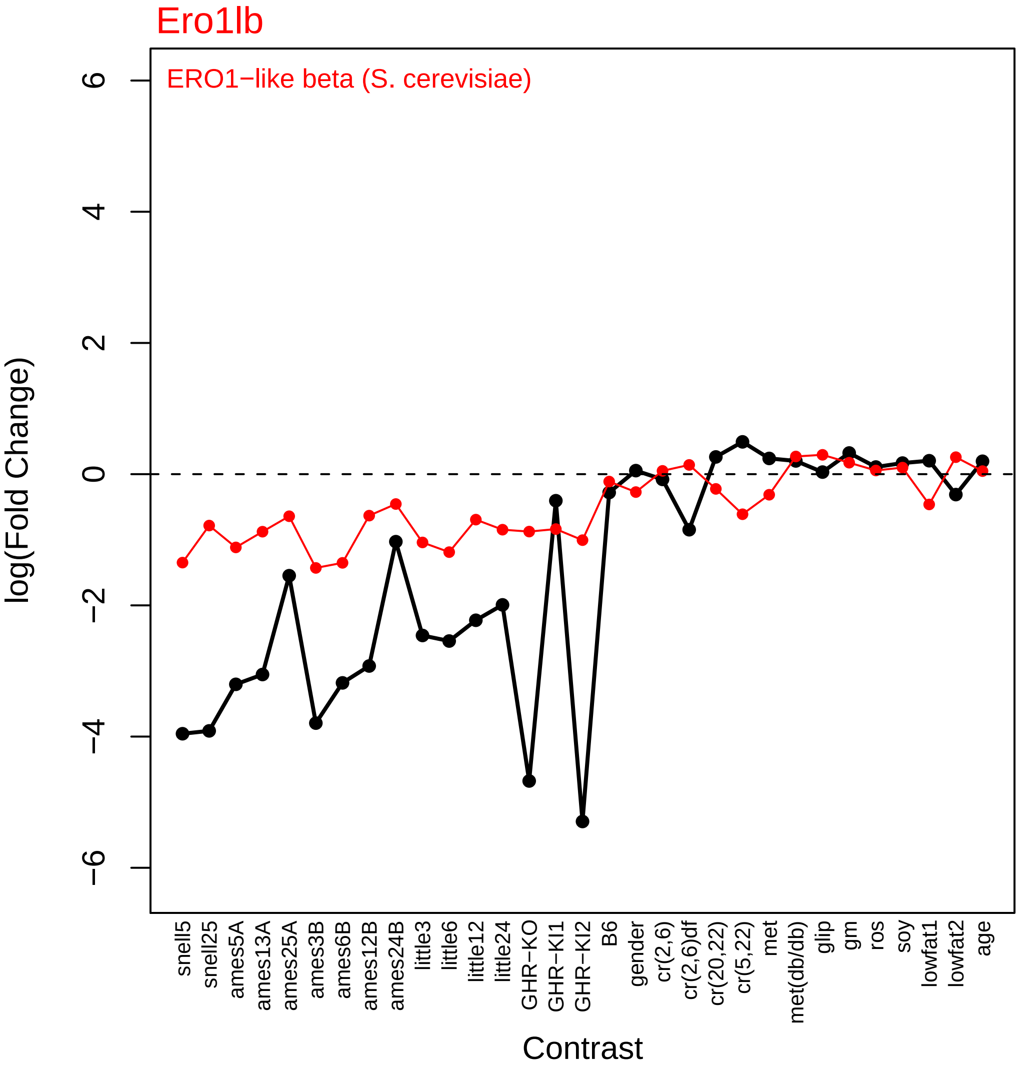
Ero1lb
ERO1−like beta (S. cerevisiae)
log(Fold Change)
6
4
2
0
−2
−4
−6
snell5
snell25
ames5A
ames13A
ames25A
ames3B
ames6B
ames12B
ames24B
little3
little6
little12
little24
GHR−KO
GHR−KI1
GHR−KI2
B6
gender
cr(2,6)
cr(2,6)df
cr(20,22)
cr(5,22)
met
met(db/db)
glip
gm
ros
soy
lowfat1
lowfat2
age
Contrast

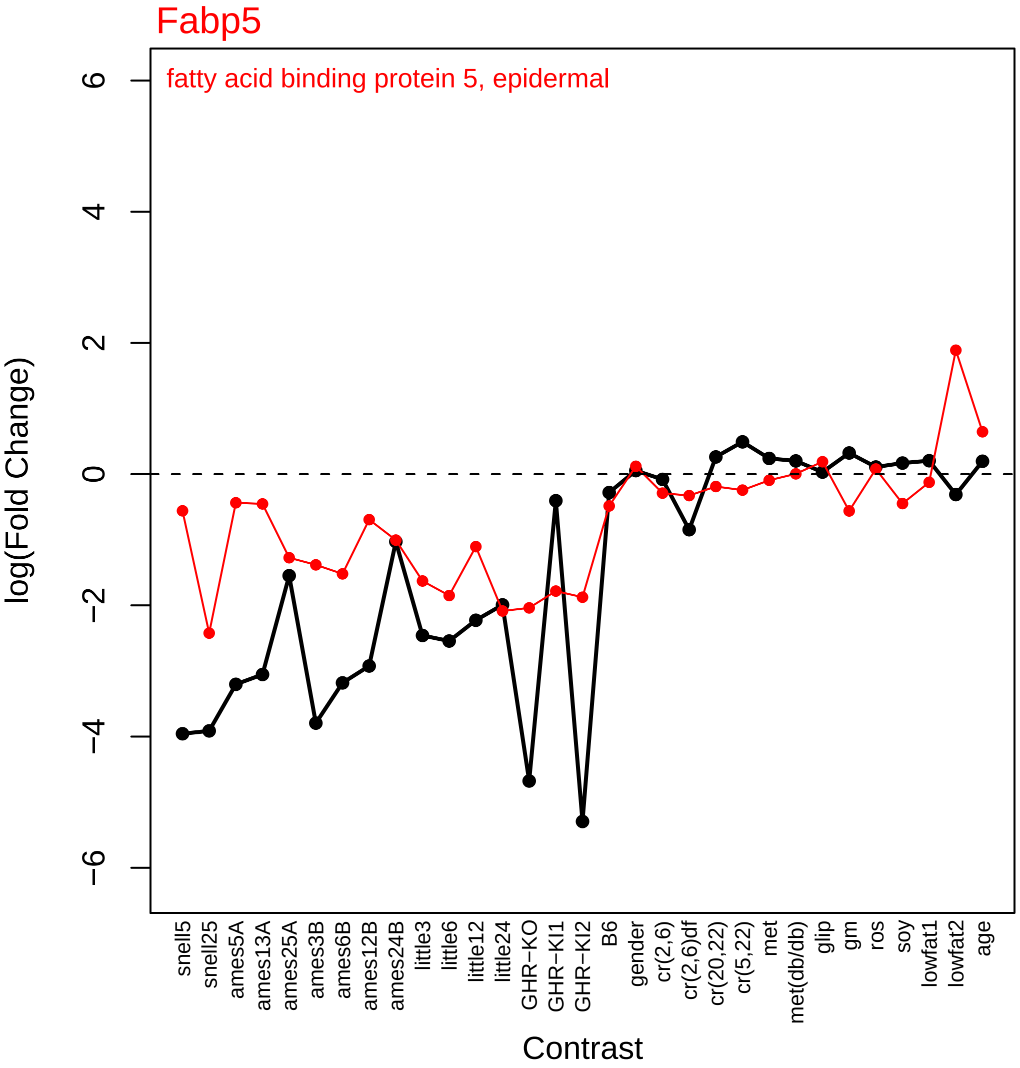

Fabp5
fatty acid binding protein 5, epidermal
log(Fold Change)
6
4
2
0
−2
−4
−6
snell5
snell25
ames5A
ames13A
ames25A
ames3B
ames6B
ames12B
ames24B
little3
little6
little12
little24
GHR−KO
GHR−KI1
GHR−KI2
B6
gender
cr(2,6)
cr(2,6)df
cr(20,22)
cr(5,22)
met
met(db/db)
glip
gm
ros
soy
lowfat1
lowfat2
age
Contrast

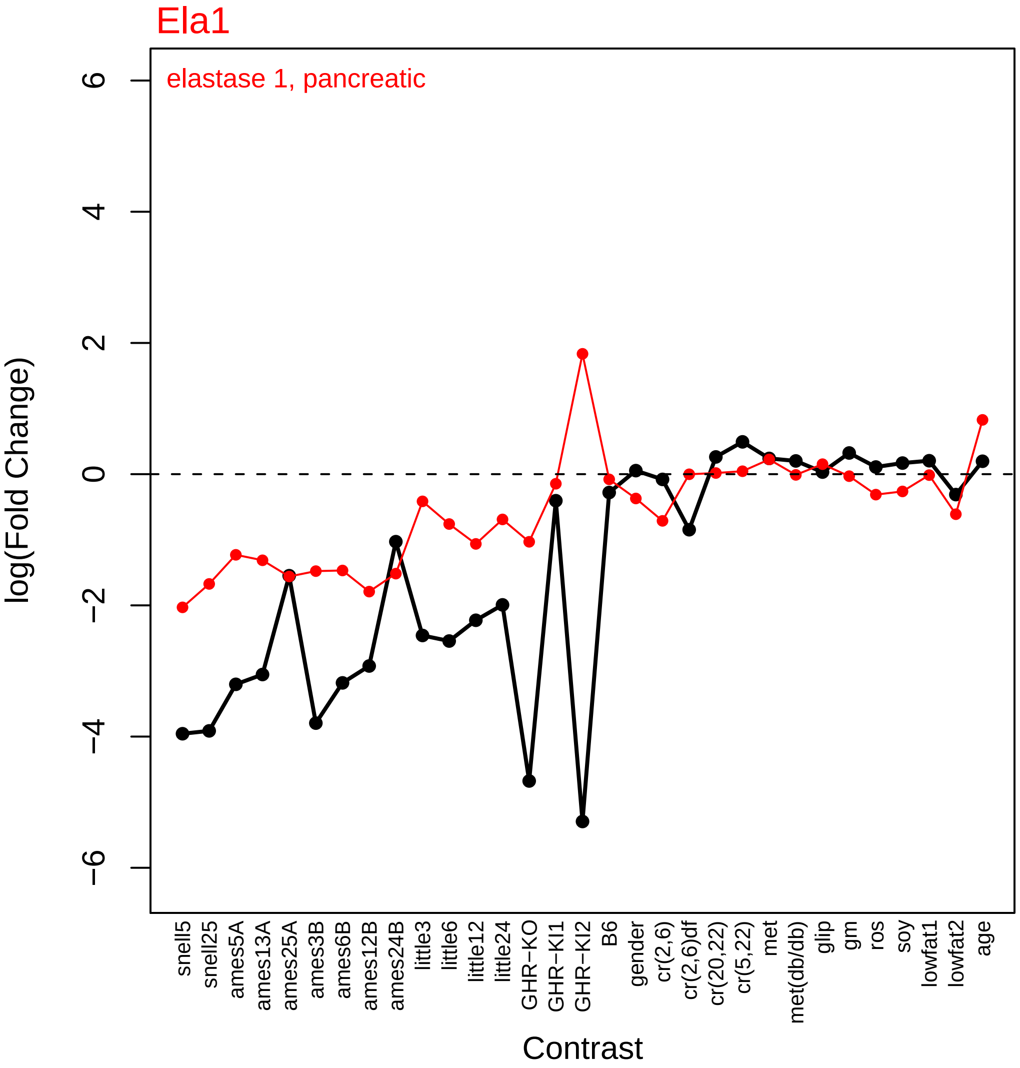
Ela1
elastase 1, pancreatic
log(Fold Change)
6
4
2
0
−2
−4
−6
snell5
snell25
ames5A
ames13A
ames25A
ames3B
ames6B
ames12B
ames24B
little3
little6
little12
little24
GHR−KO
GHR−KI1
GHR−KI2
B6
gender
cr(2,6)
cr(2,6)df
cr(20,22)
cr(5,22)
met
met(db/db)
glip
gm
ros
soy
lowfat1
lowfat2
age
Contrast

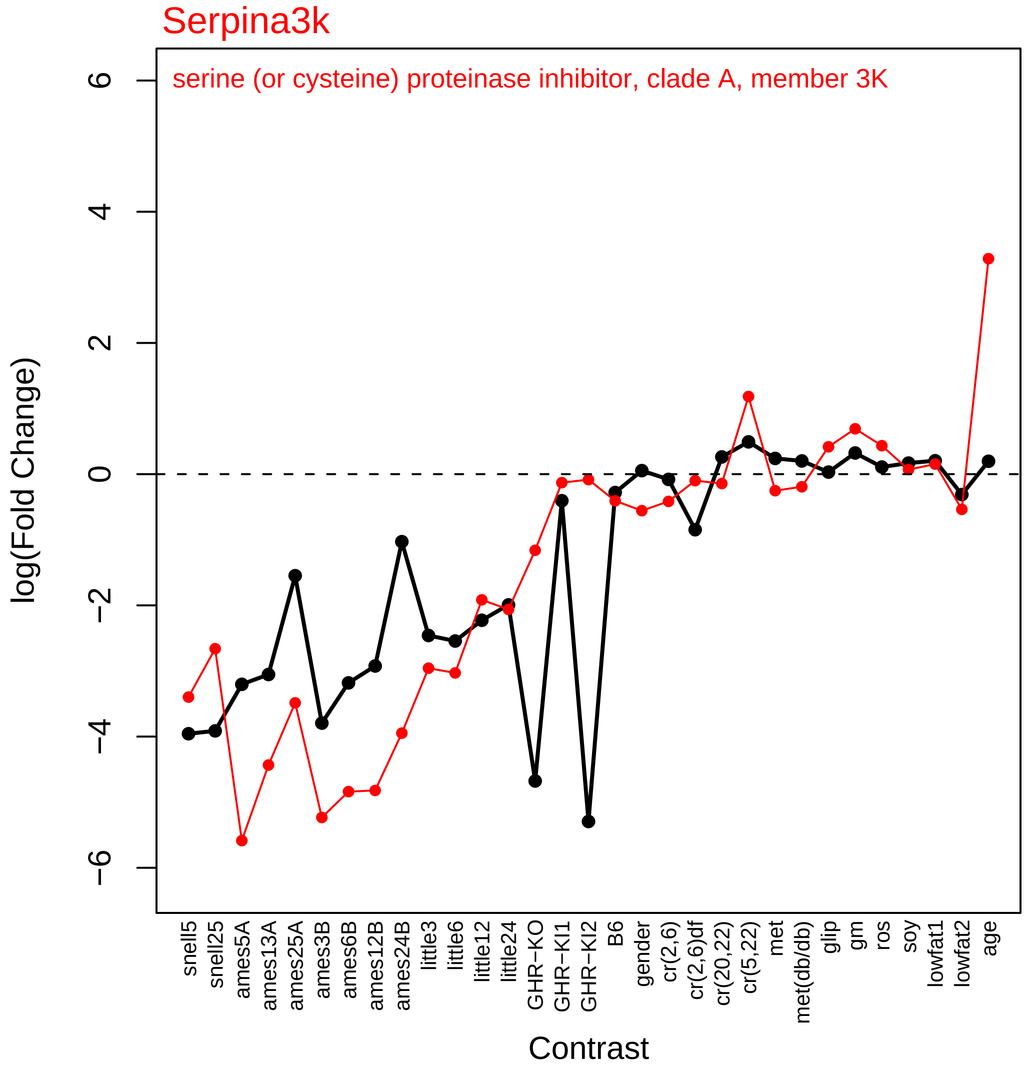
Serpina3k
serine (or cysteine) proteinase inhibitor, clade A, member 3K
log(Fold Change)
6
4
2
0
−2
−4
−6
snell5
snell25
ames5A
ames13A
ames25A
ames3B
ames6B
ames12B
ames24B
little3
little6
little12
little24
GHR−KO
GHR−KI1
GHR−KI2
B6
gender
cr(2,6)
cr(2,6)df
cr(20,22)
cr(5,22)
met
met(db/db)
glip
gm
ros
soy
lowfat1
lowfat2
age
Contrast

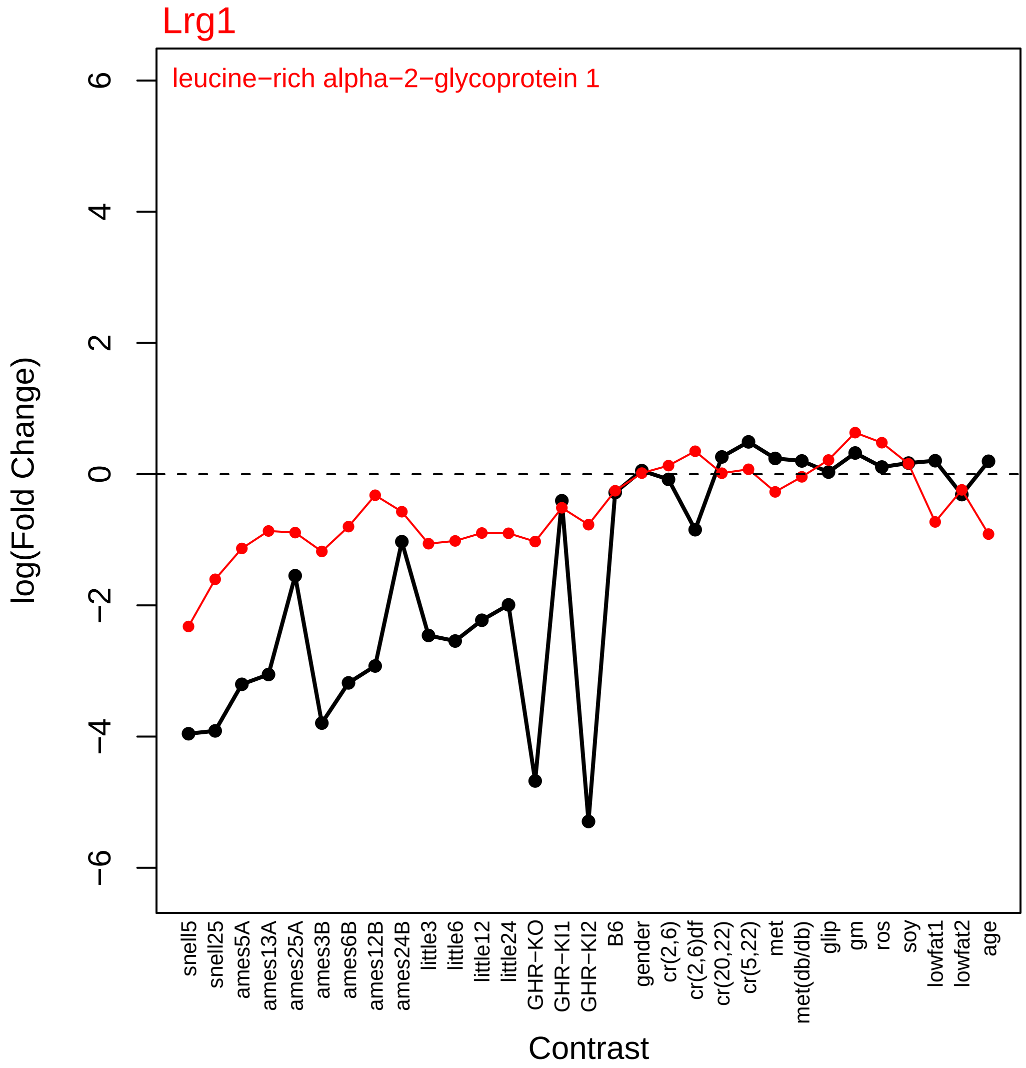

Lrg1
leucine−rich alpha−2−glycoprotein 1
log(Fold Change)
6
4
2
0
−2
−4
−6
snell5
snell25
ames5A
ames13A
ames25A
ames3B
ames6B
ames12B
ames24B
little3
little6
little12
little24
GHR−KO
GHR−KI1
GHR−KI2
B6
gender
cr(2,6)
cr(2,6)df
cr(20,22)
cr(5,22)
met
met(db/db)
glip
gm
ros
soy
lowfat1
lowfat2
age
Contrast

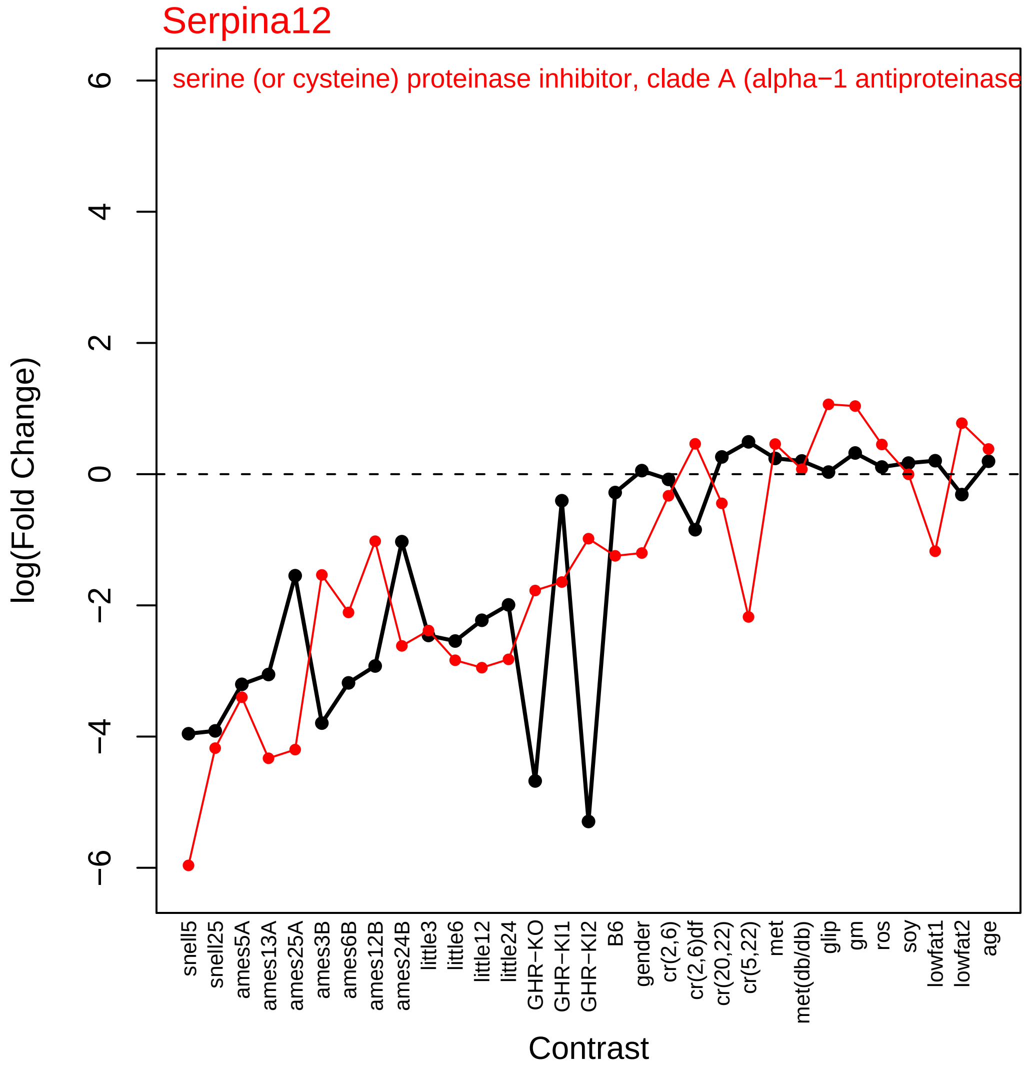

Serpina12
serine (or cysteine) proteinase inhibitor, clade A (alpha−1 antiproteinase
log(Fold Change)
6
4
2
0
−2
−4
−6
snell5
snell25
ames5A
ames13A
ames25A
ames3B
ames6B
ames12B
ames24B
little3
little6
little12
little24
GHR−KO
GHR−KI1
GHR−KI2
B6
gender
cr(2,6)
cr(2,6)df
cr(20,22)
cr(5,22)
met
met(db/db)
glip
gm
ros
soy
lowfat1
lowfat2
age
Contrast

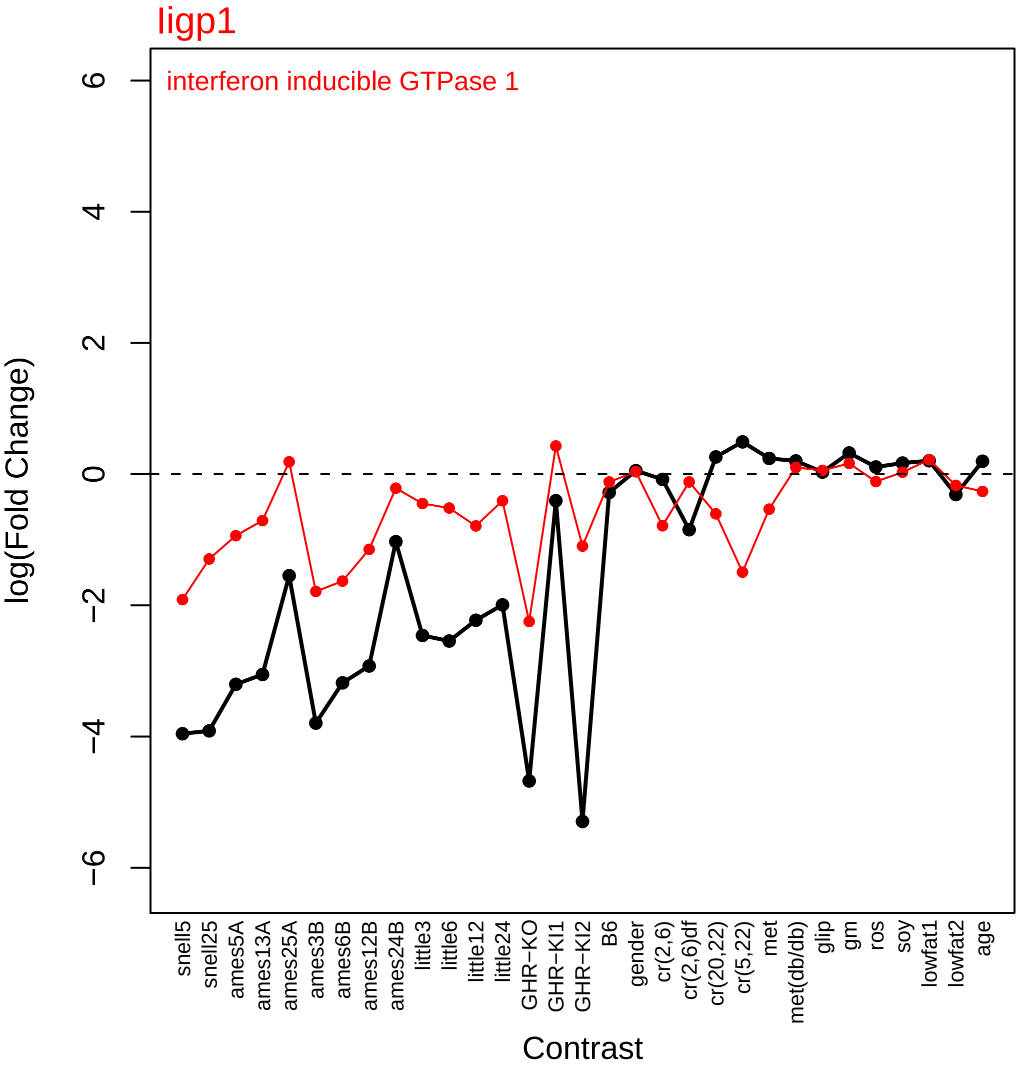

Iigp1
interferon inducible GTPase 1
log(Fold Change)
6
4
2
0
−2
−4
−6
snell5
snell25
ames5A
ames13A
ames25A
ames3B
ames6B
ames12B
ames24B
little3
little6
little12
little24
GHR−KO
GHR−KI1
GHR−KI2
B6
gender
cr(2,6)
cr(2,6)df
cr(20,22)
cr(5,22)
met
met(db/db)
glip
gm
ros
soy
lowfat1
lowfat2
age
Contrast

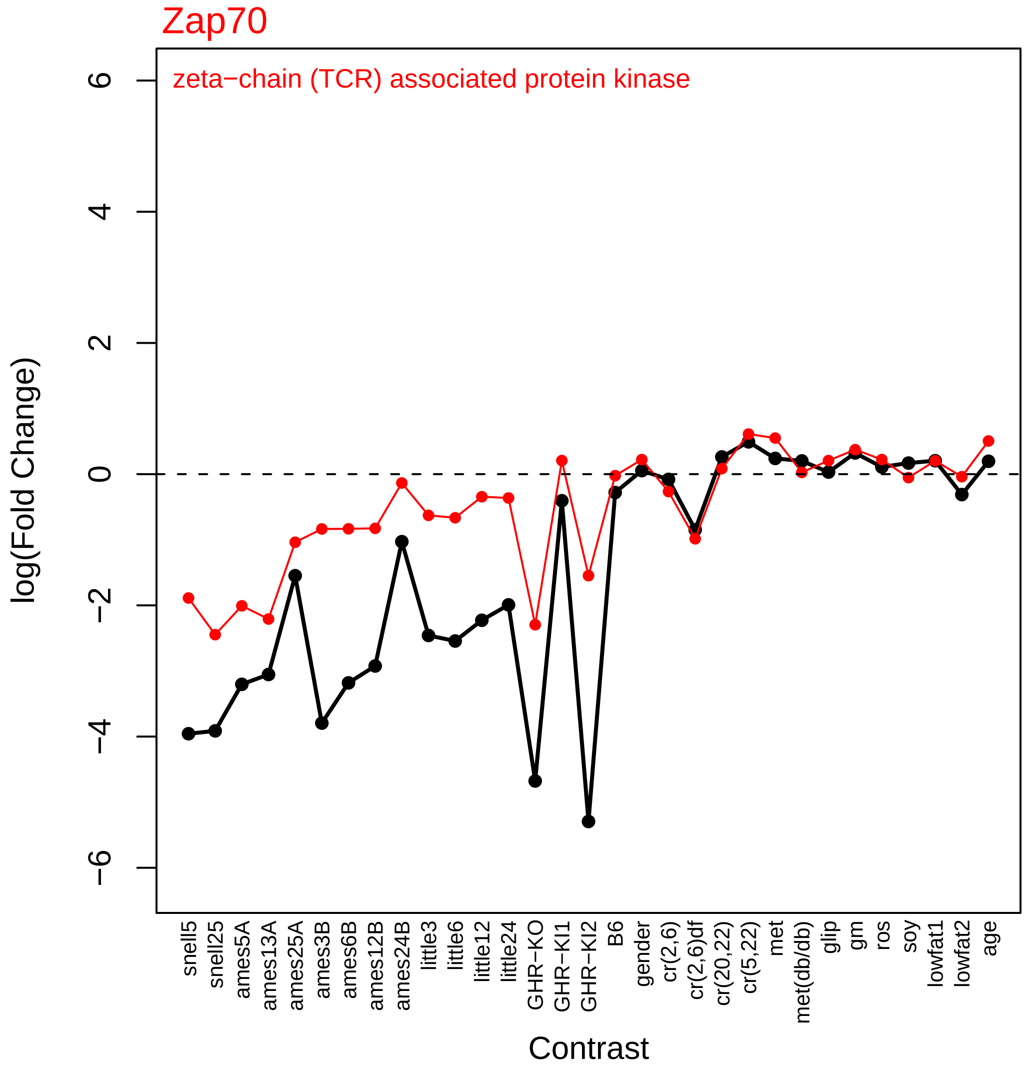

Zap70
zeta−chain (TCR) associated protein kinase
log(Fold Change)
6
4
2
0
−2
−4
−6
snell5
snell25
ames5A
ames13A
ames25A
ames3B
ames6B
ames12B
ames24B
little3
little6
little12
little24
GHR−KO
GHR−KI1
GHR−KI2
B6
gender
cr(2,6)
cr(2,6)df
cr(20,22)
cr(5,22)
met
met(db/db)
glip
gm
ros
soy
lowfat1
lowfat2
age
Contrast

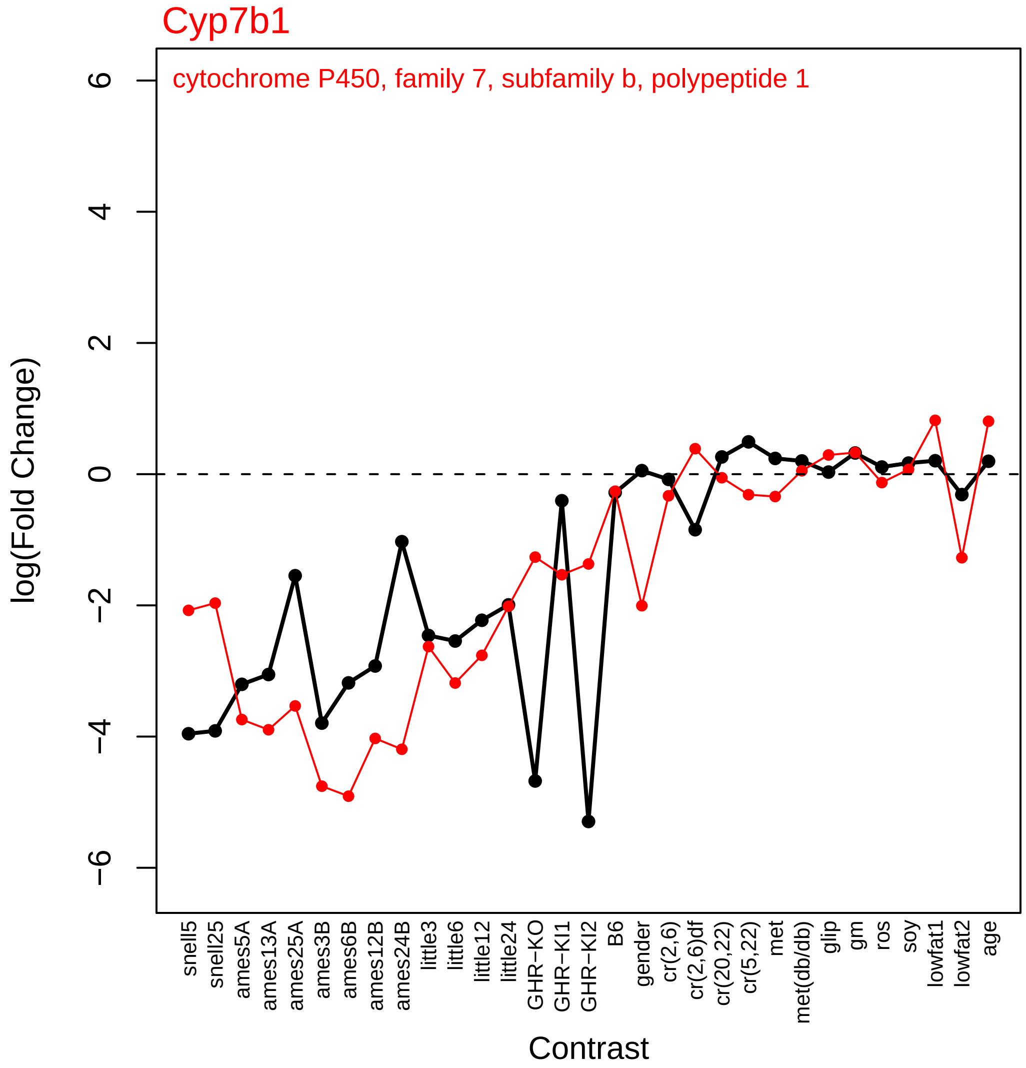

Cyp7b1
cytochrome P450, family 7, subfamily b, polypeptide 1
log(Fold Change)
6
4
2
0
−2
−4
−6
snell5
snell25
ames5A
ames13A
ames25A
ames3B
ames6B
ames12B
ames24B
little3
little6
little12
little24
GHR−KO
GHR−KI1
GHR−KI2
B6
gender
cr(2,6)
cr(2,6)df
cr(20,22)
cr(5,22)
met
met(db/db)
glip
gm
ros
soy
lowfat1
lowfat2
age
Contrast

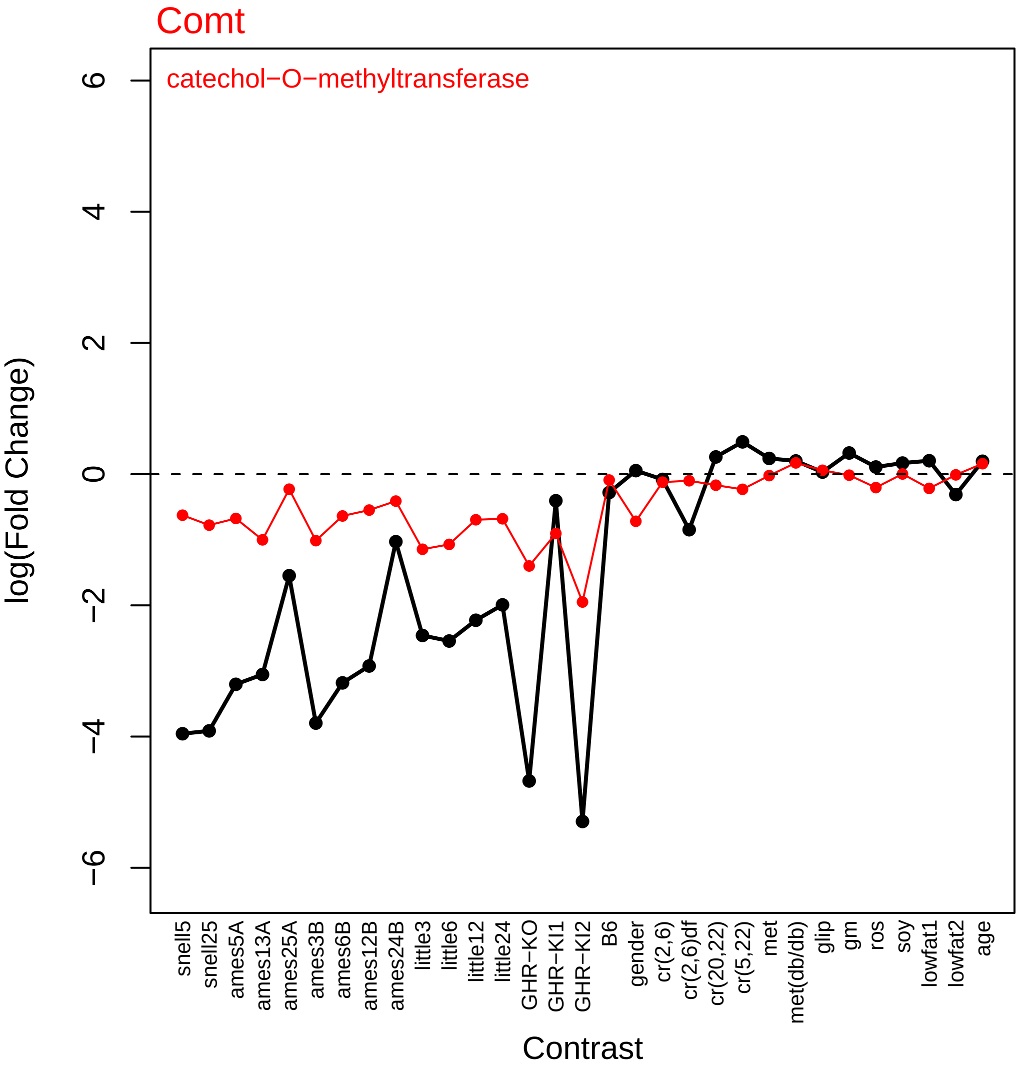
Comt
catechol−O−methyltransferase
log(Fold Change)
6
4
2
0
−2
−4
−6
Contrast
snell5
snell25
ames5A
ames13A
ames25A
ames3B
ames6B
ames12B
ames24B
little3
little6
little12
little24
GHR−KO
GHR−KI1
GHR−KI2
B6
gender
cr(2,6)
cr(2,6)df
cr(20,22)
cr(5,22)
met
met(db/db)
glip
gm
ros
soy
lowfat1
lowfat2
age

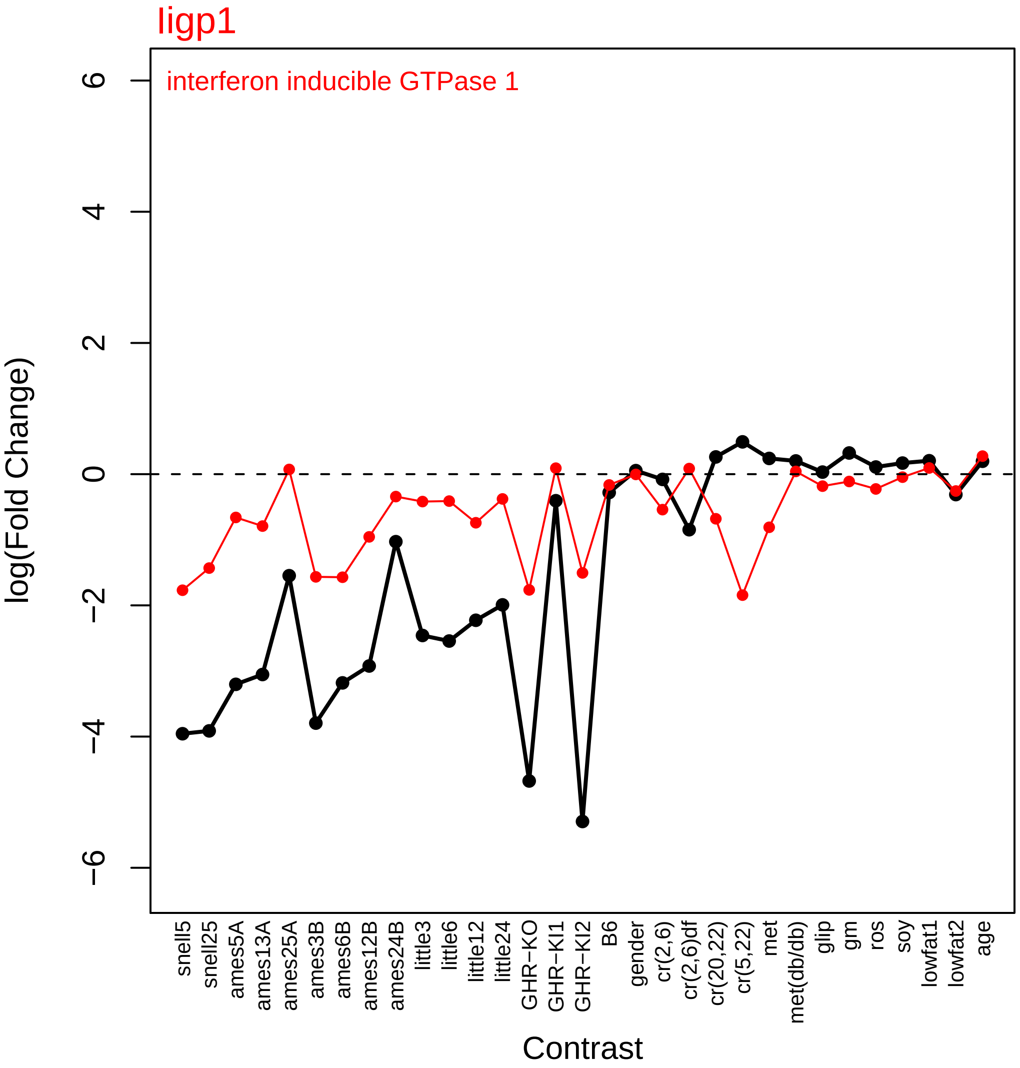

Iigp1
interferon inducible GTPase 1
log(Fold Change)
6
4
2
0
−2
−4
−6
snell5
snell25
ames5A
ames13A
ames25A
ames3B
ames6B
ames12B
ames24B
little3
little6
little12
little24
GHR−KO
GHR−KI1
GHR−KI2
B6
gender
cr(2,6)
cr(2,6)df
cr(20,22)
cr(5,22)
met
met(db/db)
glip
gm
ros
soy
lowfat1
lowfat2
age
Contrast

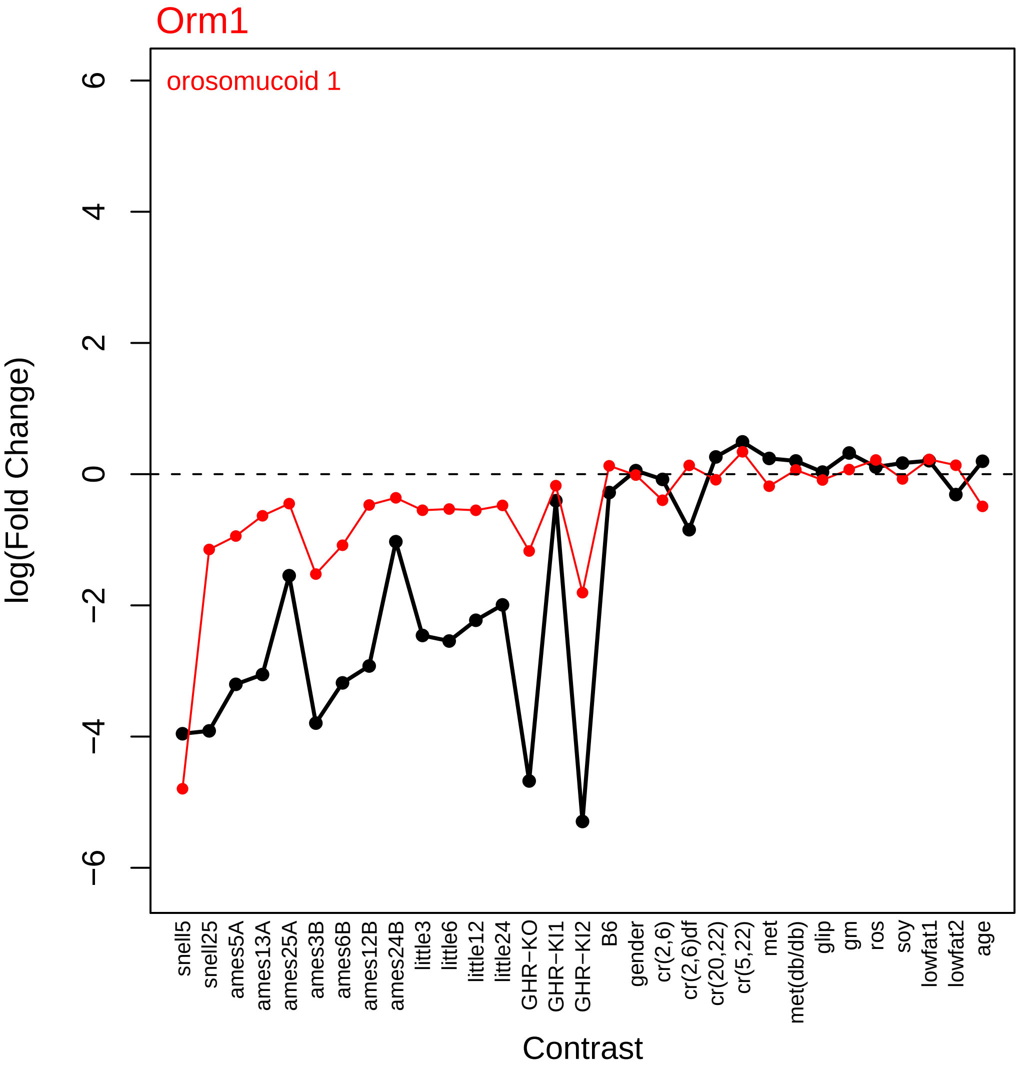

Orm1
orosomucoid 1
log(Fold Change)
6
4
2
0
−2
−4
−6
snell5
snell25
ames5A
ames13A
ames25A
ames3B
ames6B
ames12B
ames24B
little3
little6
little12
little24
GHR−KO
GHR−KI1
GHR−KI2
B6
gender
cr(2,6)
cr(2,6)df
cr(20,22)
cr(5,22)
met
met(db/db)
glip
gm
ros
soy
lowfat1
lowfat2
age
Contrast

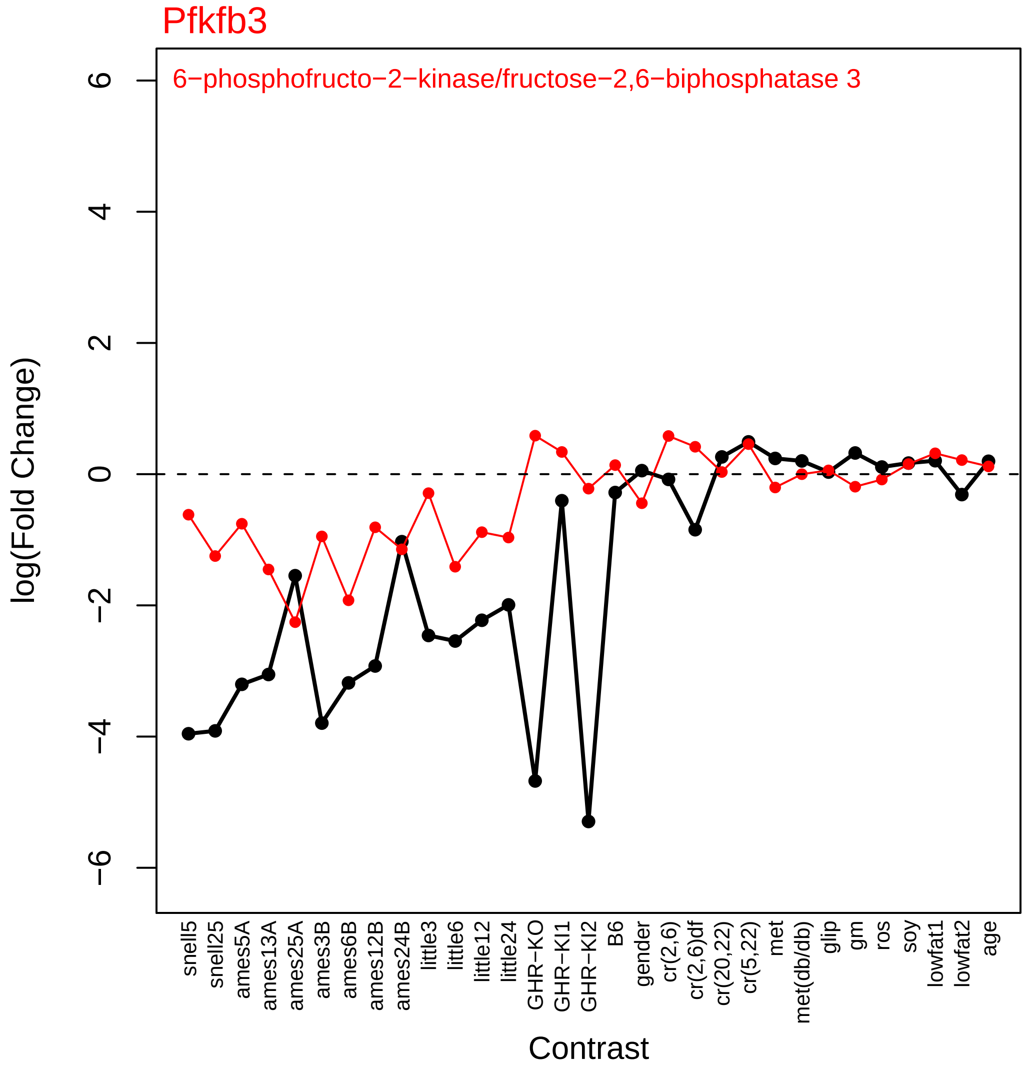
Pfkfb3
6−phosphofructo−2−kinase/fructose−2,6−biphosphatase 3
log(Fold Change)
6
4
2
0
−2
−4
−6
Contrast
snell5
snell25
ames5A
ames13A
ames25A
ames3B
ames6B
ames12B
ames24B
little3
little6
little12
little24
GHR−KO
GHR−KI1
GHR−KI2
B6
gender
cr(2,6)
cr(2,6)df
cr(20,22)
cr(5,22)
met
met(db/db)
glip
gm
ros
soy
lowfat1
lowfat2
age

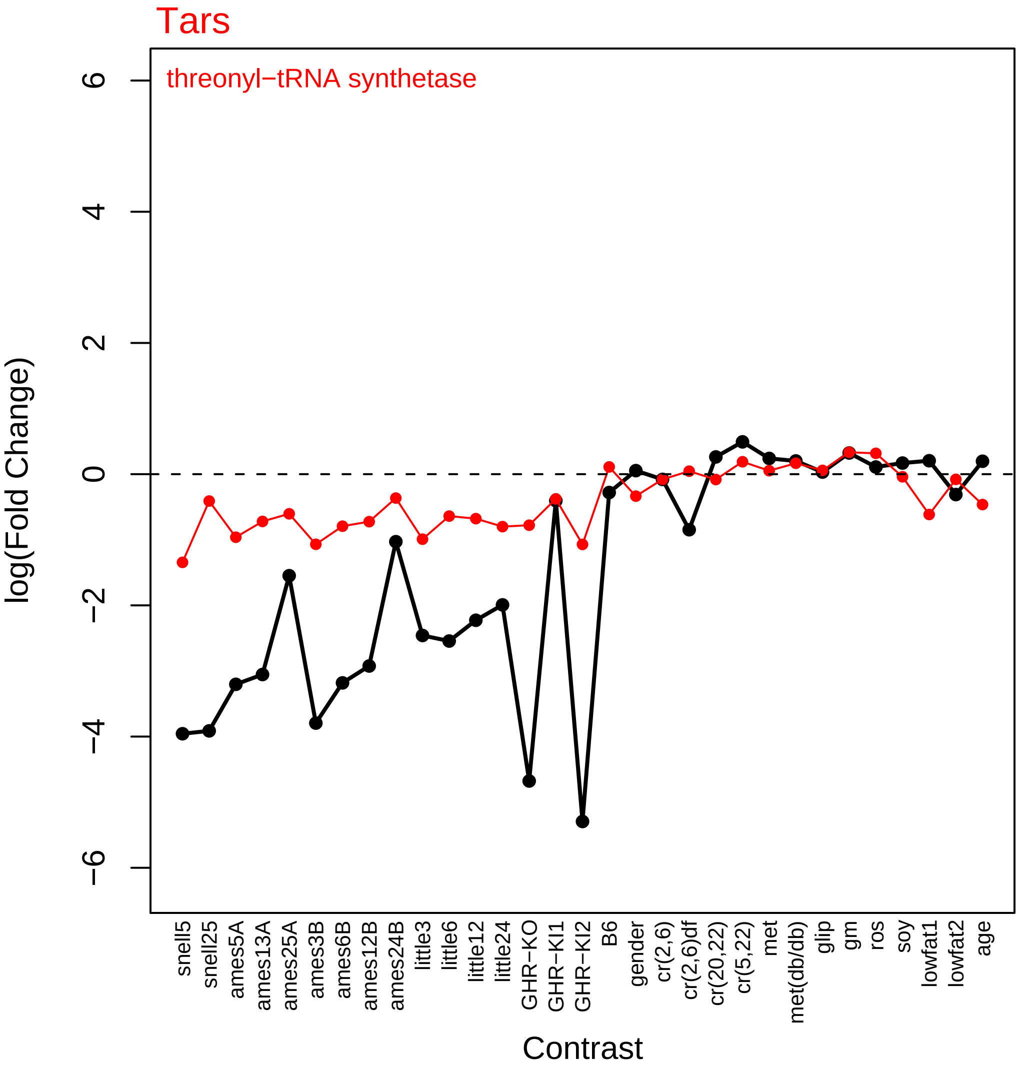

Tars
threonyl−tRNA synthetase
log(Fold Change)
6
4
2
0
−2
−4
−6
snell5
snell25
ames5A
ames13A
ames25A
ames3B
ames6B
ames12B
ames24B
little3
little6
little12
little24
GHR−KO
GHR−KI1
GHR−KI2
B6
gender
cr(2,6)
cr(2,6)df
cr(20,22)
cr(5,22)
met
met(db/db)
glip
gm
ros
soy
lowfat1
lowfat2
age
Contrast

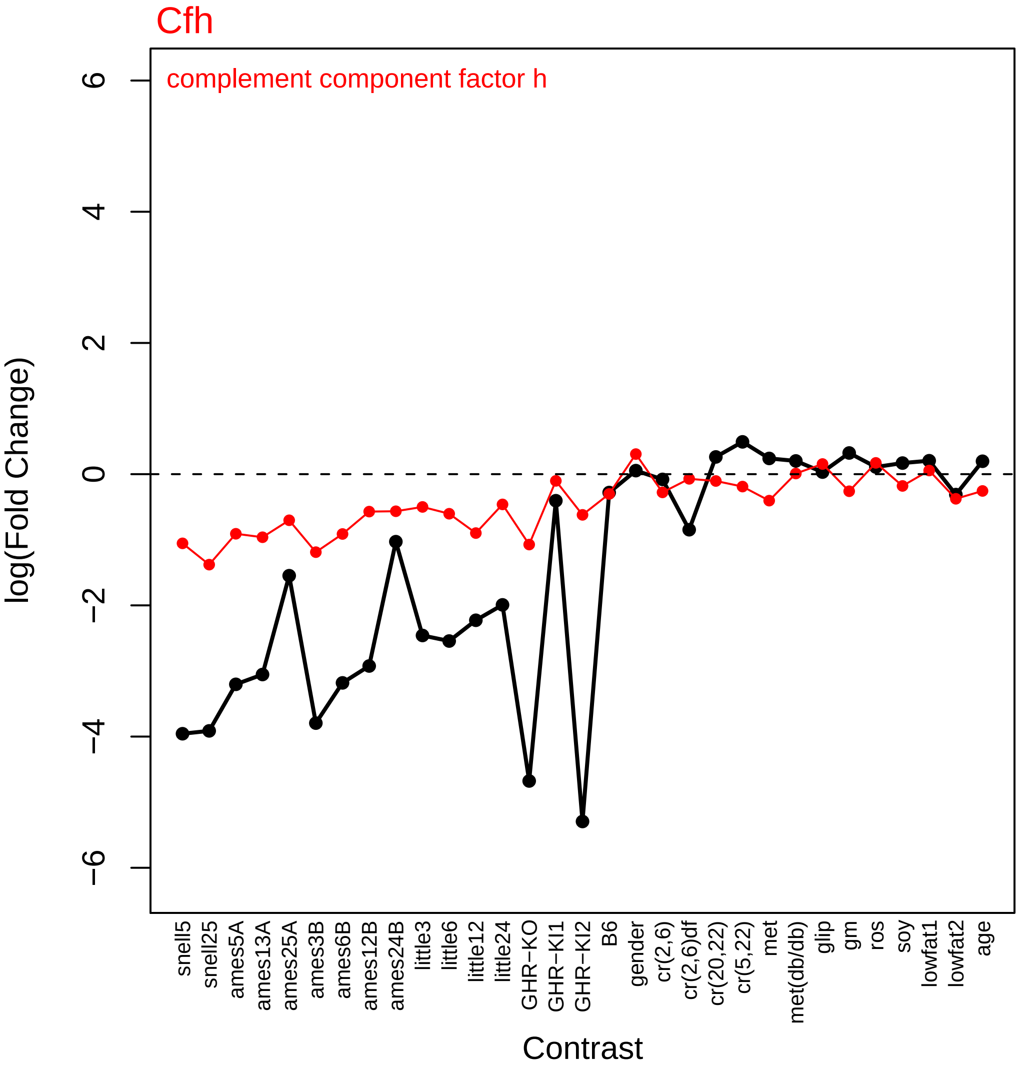
Cfh
complement component factor h
log(Fold Change)
6
4
2
0
−2
−4
−6
snell5
snell25
ames5A
ames13A
ames25A
ames3B
ames6B
ames12B
ames24B
little3
little6
little12
little24
GHR−KO
GHR−KI1
GHR−KI2
B6
gender
cr(2,6)
cr(2,6)df
cr(20,22)
cr(5,22)
met
met(db/db)
glip
gm
ros
soy
lowfat1
lowfat2
age
Contrast

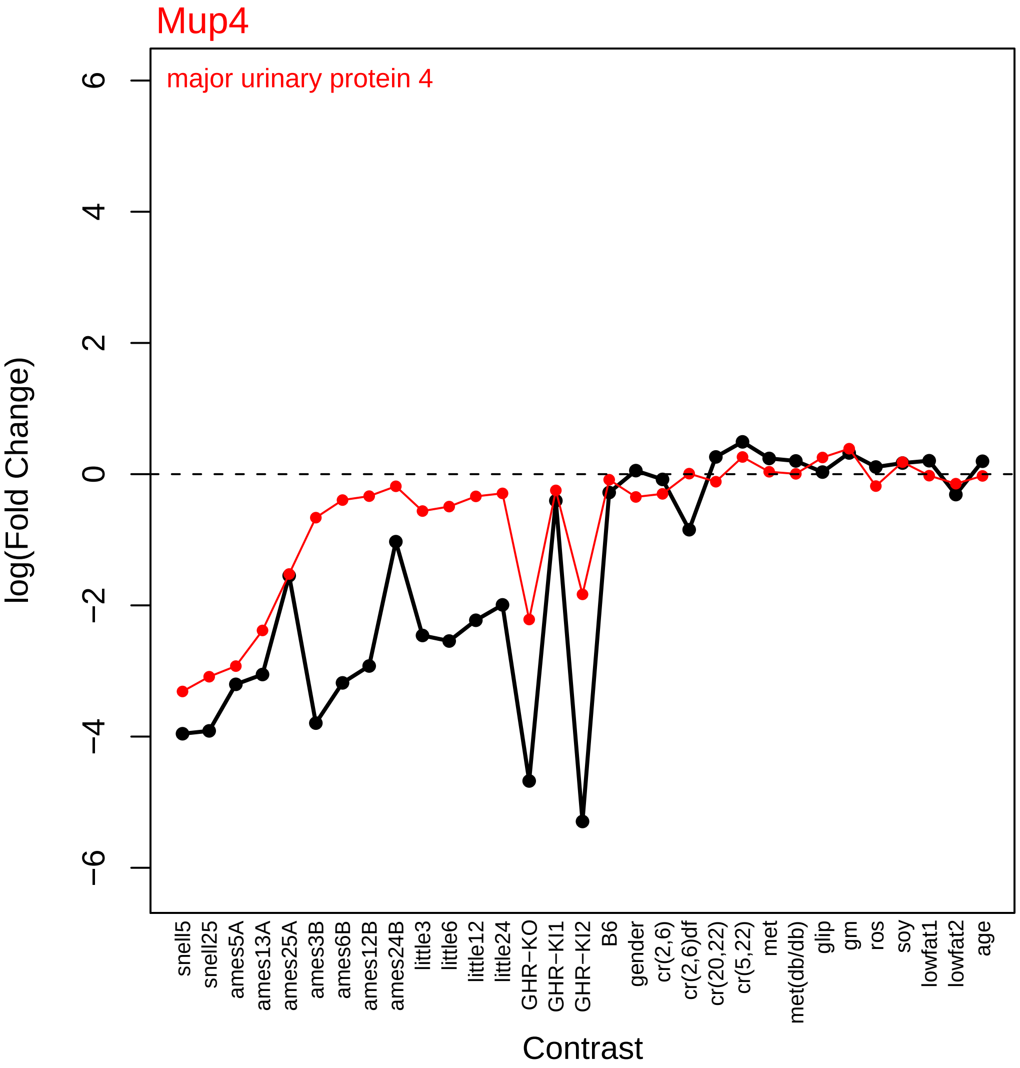
Mup4
major urinary protein 4
log(Fold Change)
6
4
2
0
−2
−4
−6
Contrast
snell5
snell25
ames5A
ames13A
ames25A
ames3B
ames6B
ames12B
ames24B
little3
little6
little12
little24
GHR−KO
GHR−KI1
GHR−KI2
B6
gender
cr(2,6)
cr(2,6)df
cr(20,22)
cr(5,22)
met
met(db/db)
glip
gm
ros
soy
lowfat1
lowfat2
age

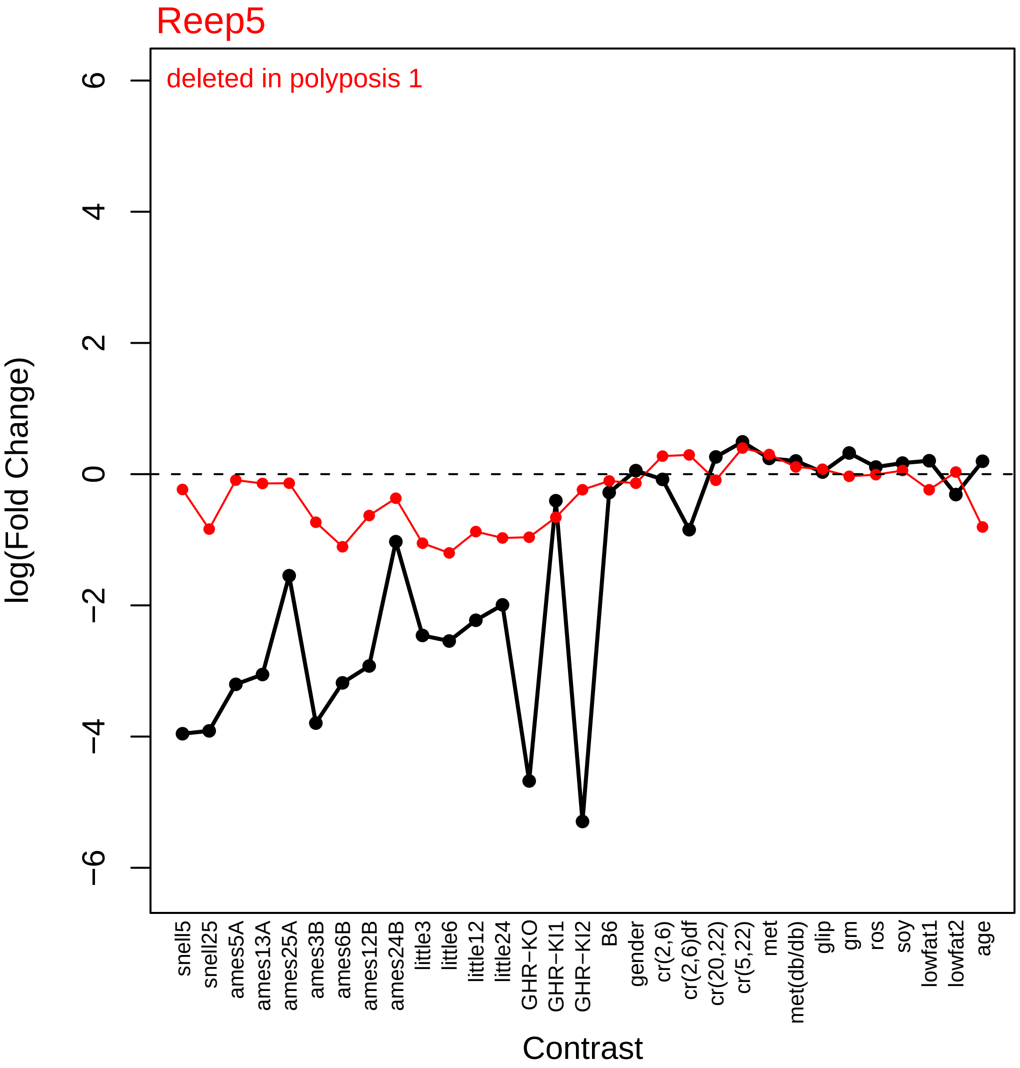

Reep5
deleted in polyposis 1
log(Fold Change)
6
4
2
0
−2
−4
−6
snell5
snell25
ames5A
ames13A
ames25A
ames3B
ames6B
ames12B
ames24B
little3
little6
little12
little24
GHR−KO
GHR−KI1
GHR−KI2
B6
gender
cr(2,6)
cr(2,6)df
cr(20,22)
cr(5,22)
met
met(db/db)
glip
gm
ros
soy
lowfat1
lowfat2
age
Contrast
