# Supplementary material for: Gene expression profiling of long-lived dwarf mice: longevity-associated genes and relationships with diet, gender and aging
Source: BMC Genomics. 2007 Oct 3;8:353. doi: 10.1186/1471-2164-8-353 (PMC2094713; doi:10.1186/1471-2164-8-353)
Supplement: Additional file 2 — Genes negatively associated with IGF-I expression. This file displays expression response profiles for the top 40 genes most negatively associated with IGF-I induction patterns among all contrasts examined in this study (see Fig. 2). In each plot, the black line represents the IGF-I induction pattern among contrasts, and the green line represents the pattern associated with a gene that exhibits an opposite induction pattern. Genes were selected by reflecting the induction pattern of IGF-I about the zero horizontal, and finding genes with an induction pattern most similar to this IGF-I reflection. Genes are presented in order of decreasing similarity to the IGF-I reflection. Following appropriate normalization to weight all contrasts equally, similarity was determined based on Euclidean distance. [file 1471-2164-8-353-S2.pdf]

Additional File 2:

Gene expression profiling of long−lived dwarf mice: longevity−associated genes and relationships with diet, gender and aging

William R. Swindell

This file displays expression response profiles for the top 40 genes most negatively associated with IGF−1 induction patterns among all contrasts examined in this study (see Fig. 2 of paper). These genes are listed on pages 2−3 and plotted individually on pages 4−43 of this file. In each plot, the black line represents the IGF−1 induction pattern among contrasts, and the green line represents the pattern associated with a gene that exhibits an opposite induction pattern. Genes were selected by reflecting the induction pattern of IGF−1 about the zero horizontal, and finding genes with an induction pattern most similar to the IGF−1 reflection. Genes are presented in order of decreasing similarity to the IGF−1 reflection. Following appropriate normalization to weight all contrasts equally, similarity was determined based on Euclidean distance.

Contact: William R. Swindell, wswindel@med.umich.edu

Symbol	Affymetrix ID	Distance
Scd2	95758_at	11.871
Slc16a7	95060_at	14.232
Pcp4l1	97297_at	14.391
Dclre1a	104230_at	14.772
Igfbp1	103896_f_at	15.477
Spink3	96630_at	16.421
Lpl	95611_at	16.526
Cd36	93332_at	16.714
Abcb1a	102910_at	16.773
Cyp2b10	102701_at	16.86
Serpina6	96227_at	17.151
Gstm3	97681_f_at	17.319
Abcd2	92913_at	17.697
Cyp2a4	102847_s_at	18.193
Idh3b	95693_at	18.232
Tcea3	102344_s_at	19.061
Adssl1	98435_at	19.249
Sult1a1	103087_at	19.267
Rtn4	160484_at	19.502
Gsta4	96085_at	19.634

Symbol	Affymetrix ID	Distance
Mt1	93573_at	19.77
1810015C04Rik	95518_at	19.785
Gsta2	101872_at	19.874
Robo1	103675_at	19.884
Btg1	93104_at	19.912
Rdh16	102013_at	19.991
Cyp2c38	102084_f_at	20.004
Papss2	96713_at	20.016
Defb1	100882_at	20.235
Igfbp2	98627_at	20.237
Il1rn	93871_at	20.421
Sult1d1	160537_at	20.521
Ank3	98476_at	20.632
Slc7a2	92736_at	20.742
Nqo1	94350_f_at	20.759
Tuba8	104682_at	20.768
Agt	101887_at	20.888
Ces1	103519_at	20.998
Vnn3	104181_at	21.019
Odz3	92500_at	21.041

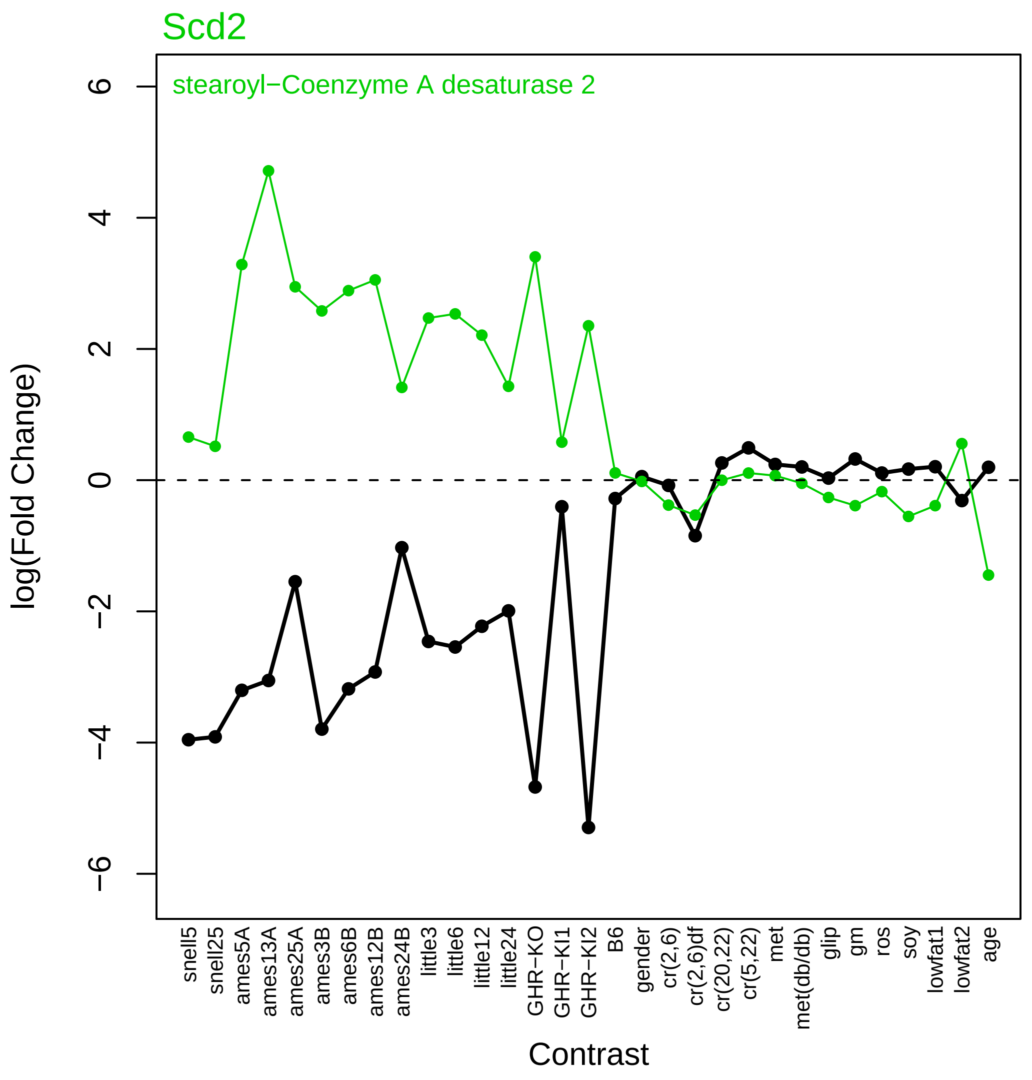

Scd2
stearoyl−Coenzyme A desaturase 2
log(Fold Change)
6
4
2
0
−2
−4
−6
snell5
snell25
ames5A
ames13A
ames25A
ames3B
ames6B
ames12B
ames24B
little3
little6
little12
little24
GHR−KO
GHR−KI1
GHR−KI2
B6
gender
cr(2,6)
cr(2,6)df
cr(20,22)
cr(5,22)
met
met(db/db)
glip
gm
ros
soy
lowfat1
lowfat2
age
Contrast

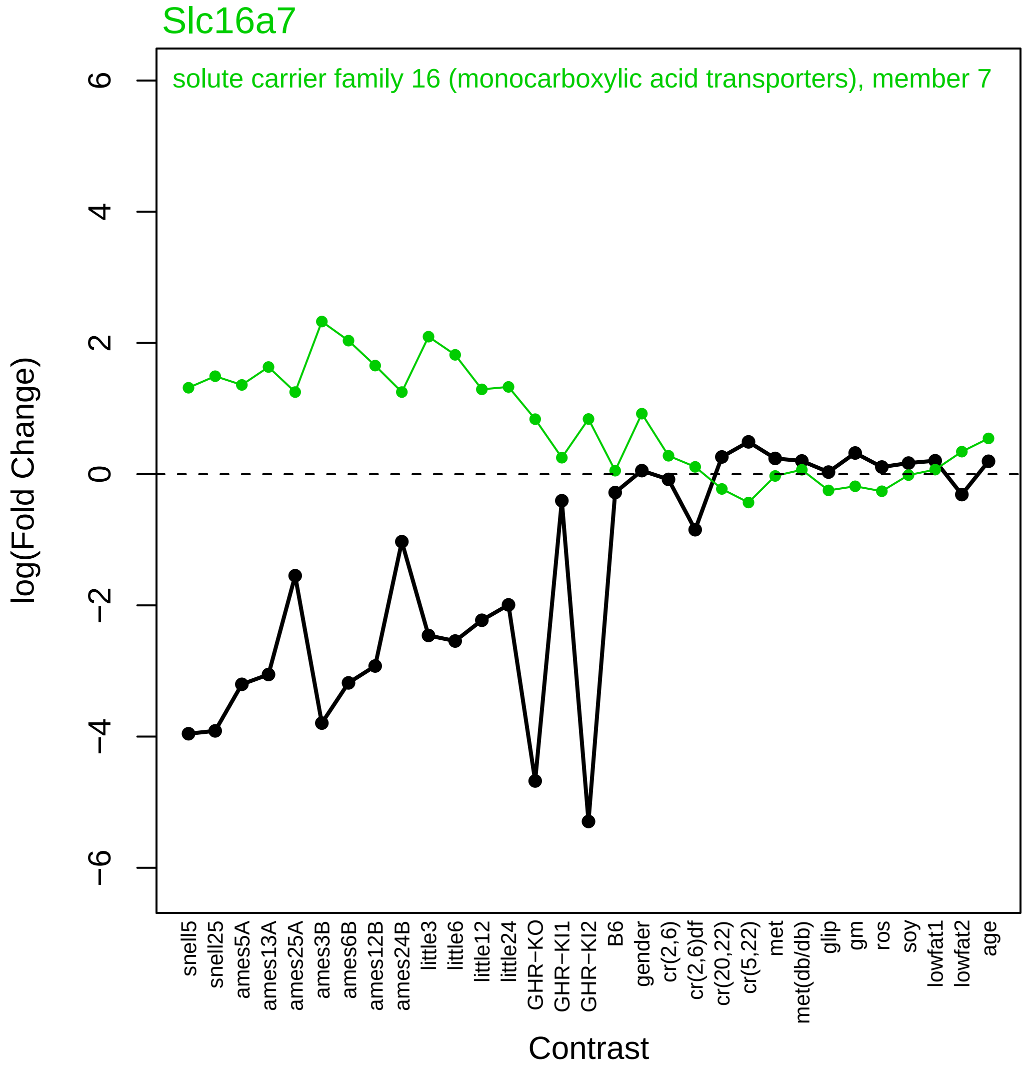

Slc16a7
solute carrier family 16 (monocarboxylic acid transporters), member 7
log(Fold Change)
6
4
2
0
−2
−4
−6
Contrast
snell5
snell25
ames5A
ames13A
ames25A
ames3B
ames6B
ames12B
ames24B
little3
little6
little12
little24
GHR−KO
GHR−KI1
GHR−KI2
B6
gender
cr(2,6)
cr(2,6)df
cr(20,22)
cr(5,22)
met
met(db/db)
glip
gm
ros
soy
lowfat1
lowfat2
age

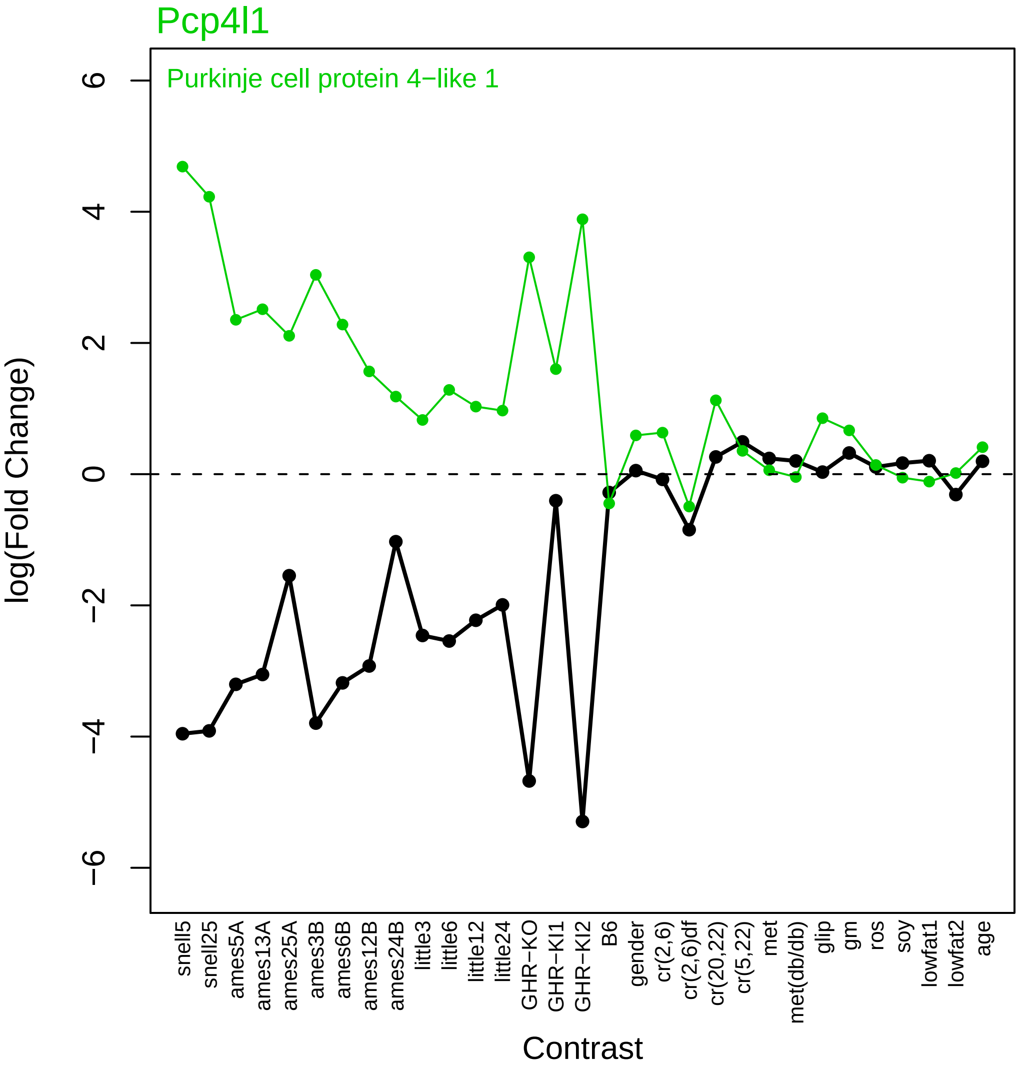

Pcp4l1
Purkinje cell protein 4−like 1
log(Fold Change)
6
4
2
0
−2
−4
−6
snell5
snell25
ames5A
ames13A
ames25A
ames3B
ames6B
ames12B
ames24B
little3
little6
little12
little24
GHR−KO
GHR−KI1
GHR−KI2
B6
gender
cr(2,6)
cr(2,6)df
cr(20,22)
cr(5,22)
met
met(db/db)
glip
gm
ros
soy
lowfat1
lowfat2
age
Contrast

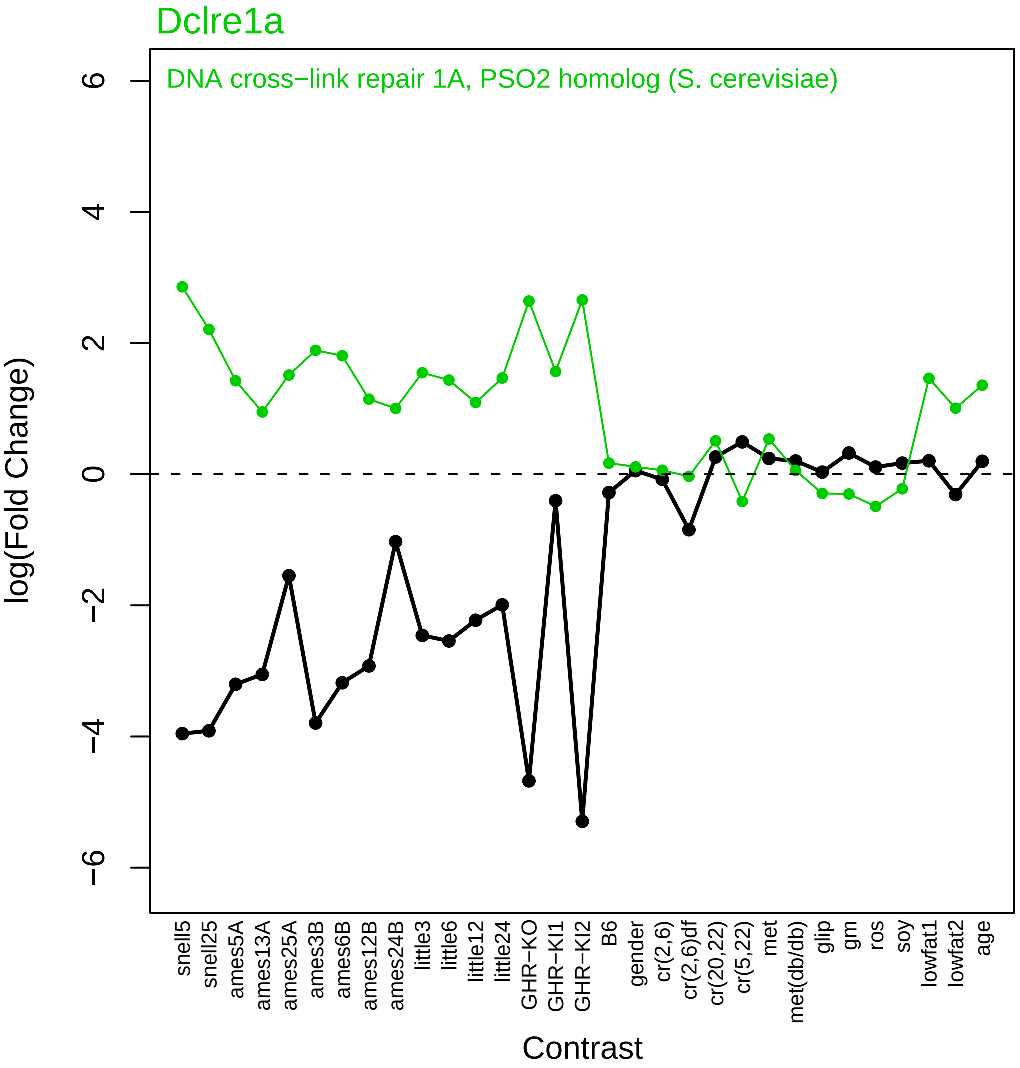
Dclre1a
DNA cross−link repair 1A, PSO2 homolog (S. cerevisiae)
log(Fold Change)
Contrast
6
4
2
0
−2
−4
−6
snell5
snell25
ames5A
ames13A
ames25A
ames3B
ames6B
ames12B
ames24B
little3
little6
little12
little24
GHR−KO
GHR−KI1
GHR−KI2
B6
gender
cr(2,6)
cr(2,6)df
cr(20,22)
cr(5,22)
met
met(db/db)
glip
gm
ros
soy
lowfat1
lowfat2
age

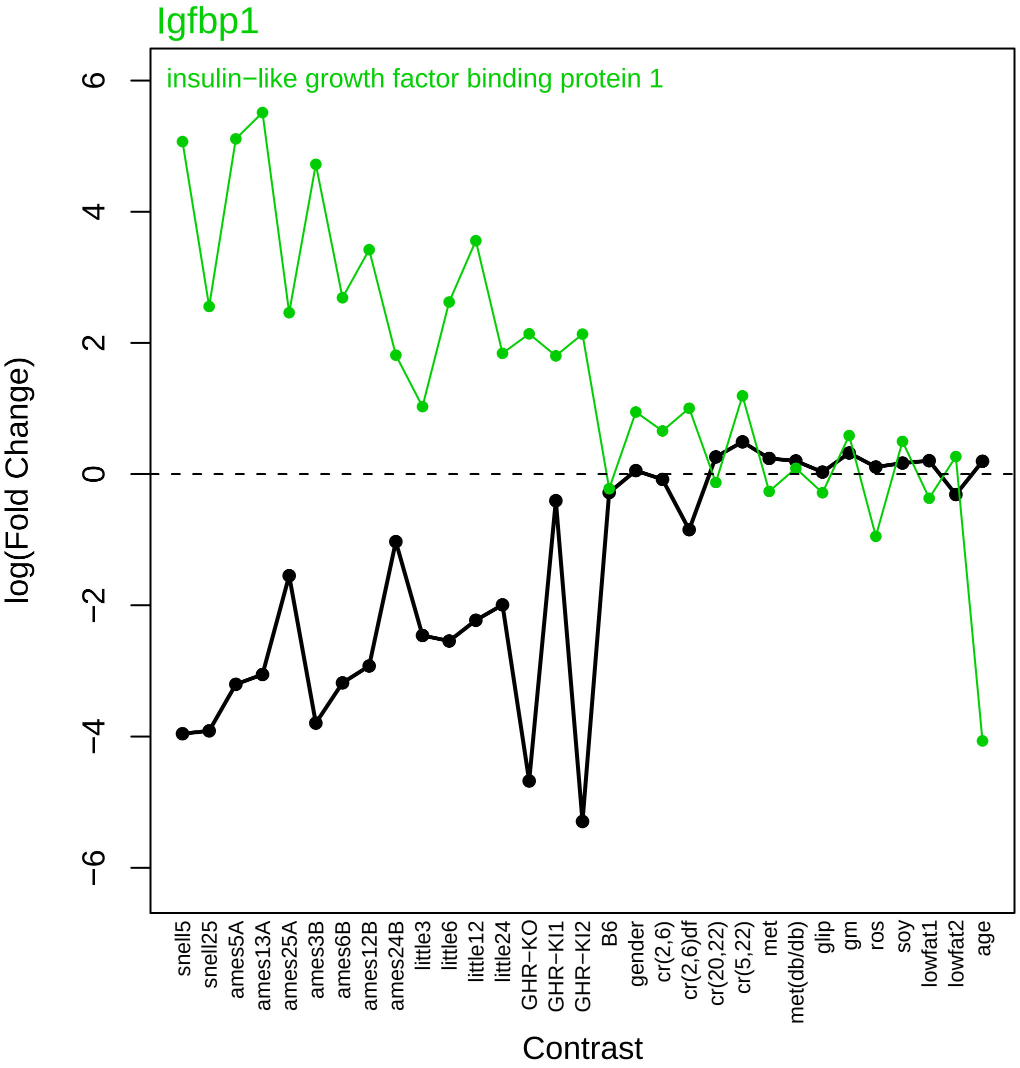

Igfbp1
insulin−like growth factor binding protein 1
log(Fold Change)
6
4
2
0
−2
−4
−6
snell5
snell25
ames5A
ames13A
ames25A
ames3B
ames6B
ames12B
ames24B
little3
little6
little12
little24
GHR−KO
GHR−KI1
GHR−KI2
B6
gender
cr(2,6)
cr(2,6)df
cr(20,22)
cr(5,22)
met
met(db/db)
glip
gm
ros
soy
lowfat1
lowfat2
age
Contrast

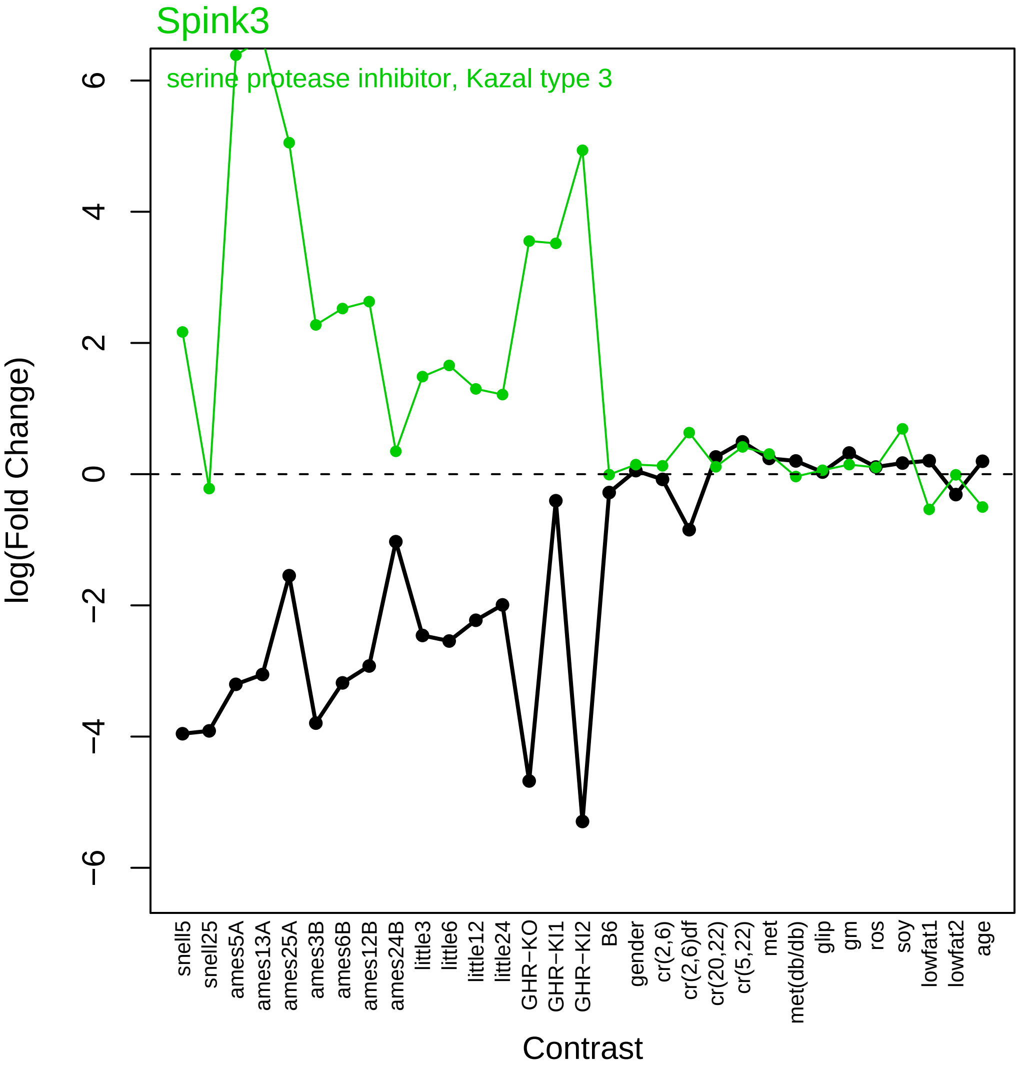

Spink3
serine protease inhibitor, Kazal type 3
log(Fold Change)
6
4
2
0
−2
−4
−6
snell5
snell25
ames5A
ames13A
ames25A
ames3B
ames6B
ames12B
ames24B
little3
little6
little12
little24
GHR−KO
GHR−KI1
GHR−KI2
B6
gender
cr(2,6)
cr(2,6)df
cr(20,22)
cr(5,22)
met
met(db/db)
glip
gm
ros
soy
lowfat1
lowfat2
age
Contrast

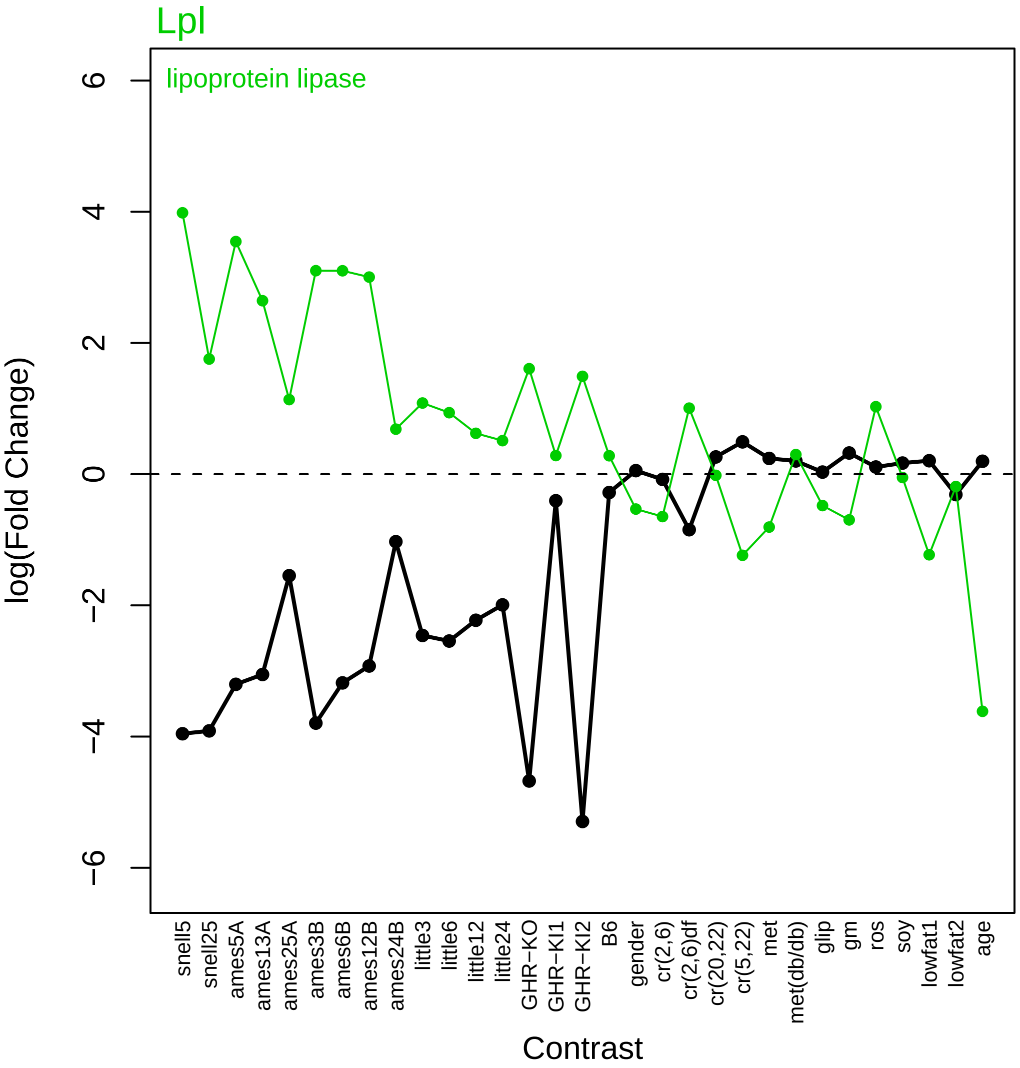
Lpl
lipoprotein lipase
log(Fold Change)
6
4
2
0
−2
−4
−6
snell5
snell25
ames5A
ames13A
ames25A
ames3B
ames6B
ames12B
ames24B
little3
little6
little12
little24
GHR−KO
GHR−KI1
GHR−KI2
B6
gender
cr(2,6)
cr(2,6)df
cr(20,22)
cr(5,22)
met
met(db/db)
glip
gm
ros
soy
lowfat1
lowfat2
age
Contrast

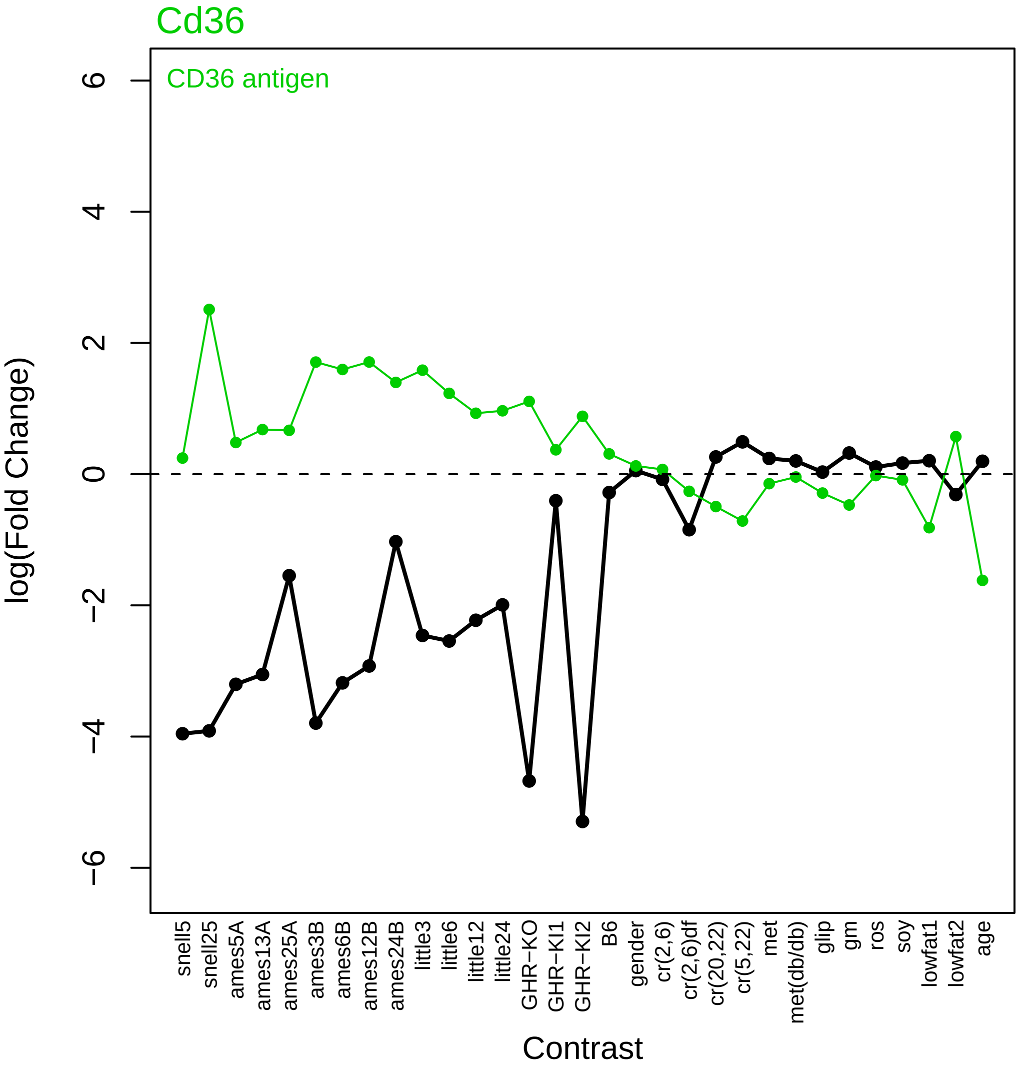
Cd36
CD36 antigen
log(Fold Change)
6
4
2
0
−2
−4
−6
snell5
snell25
ames5A
ames13A
ames25A
ames3B
ames6B
ames12B
ames24B
little3
little6
little12
little24
GHR−KO
GHR−KI1
GHR−KI2
B6
gender
cr(2,6)
cr(2,6)df
cr(20,22)
cr(5,22)
met
met(db/db)
glip
gm
ros
soy
lowfat1
lowfat2
age
Contrast

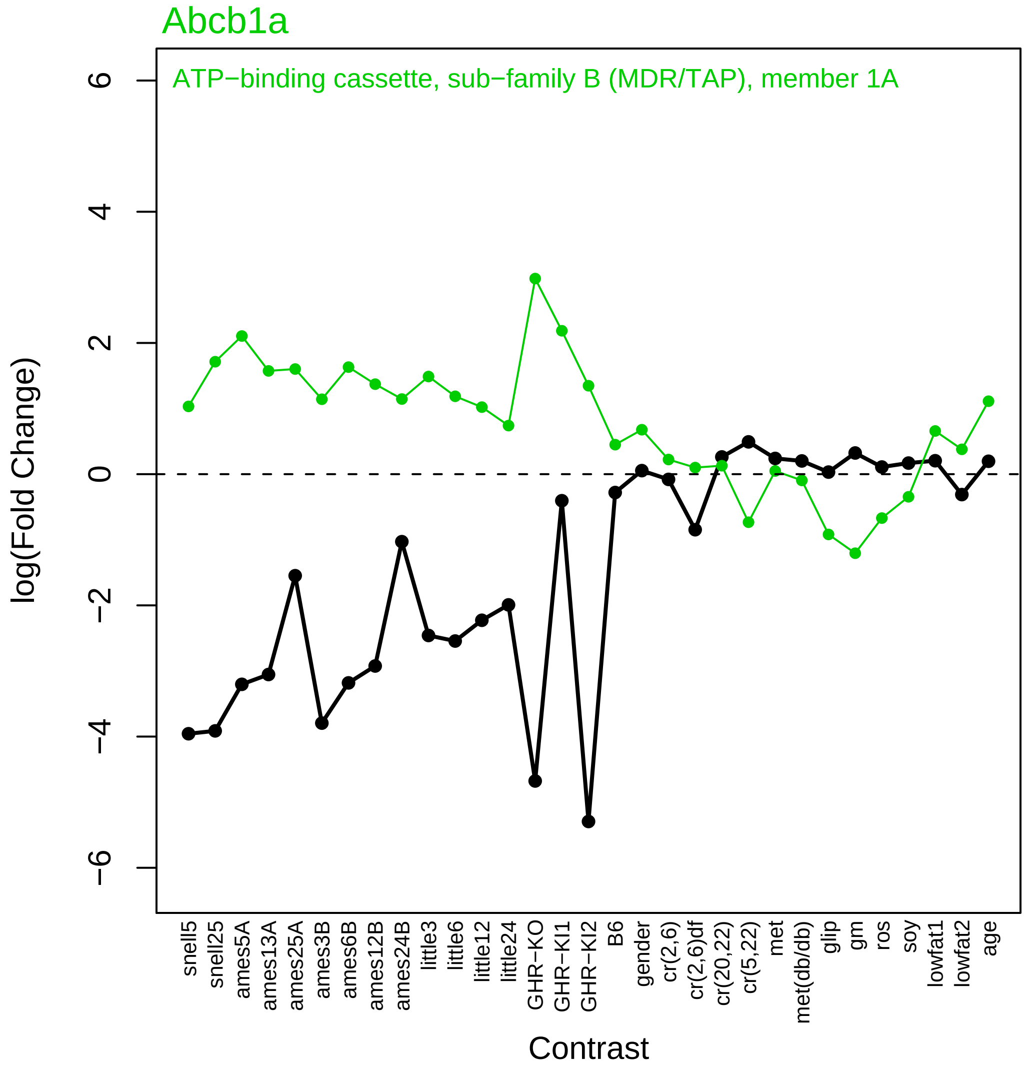
Abcb1a
ATP−binding cassette, sub−family B (MDR/TAP), member 1A
log(Fold Change)
6
4
2
0
−2
−4
−6
snell5
snell25
ames5A
ames13A
ames25A
ames3B
ames6B
ames12B
ames24B
little3
little6
little12
little24
GHR−KO
GHR−KI1
GHR−KI2
B6
gender
cr(2,6)
cr(2,6)df
cr(20,22)
cr(5,22)
met
met(db/db)
glip
gm
ros
soy
lowfat1
lowfat2
age
Contrast

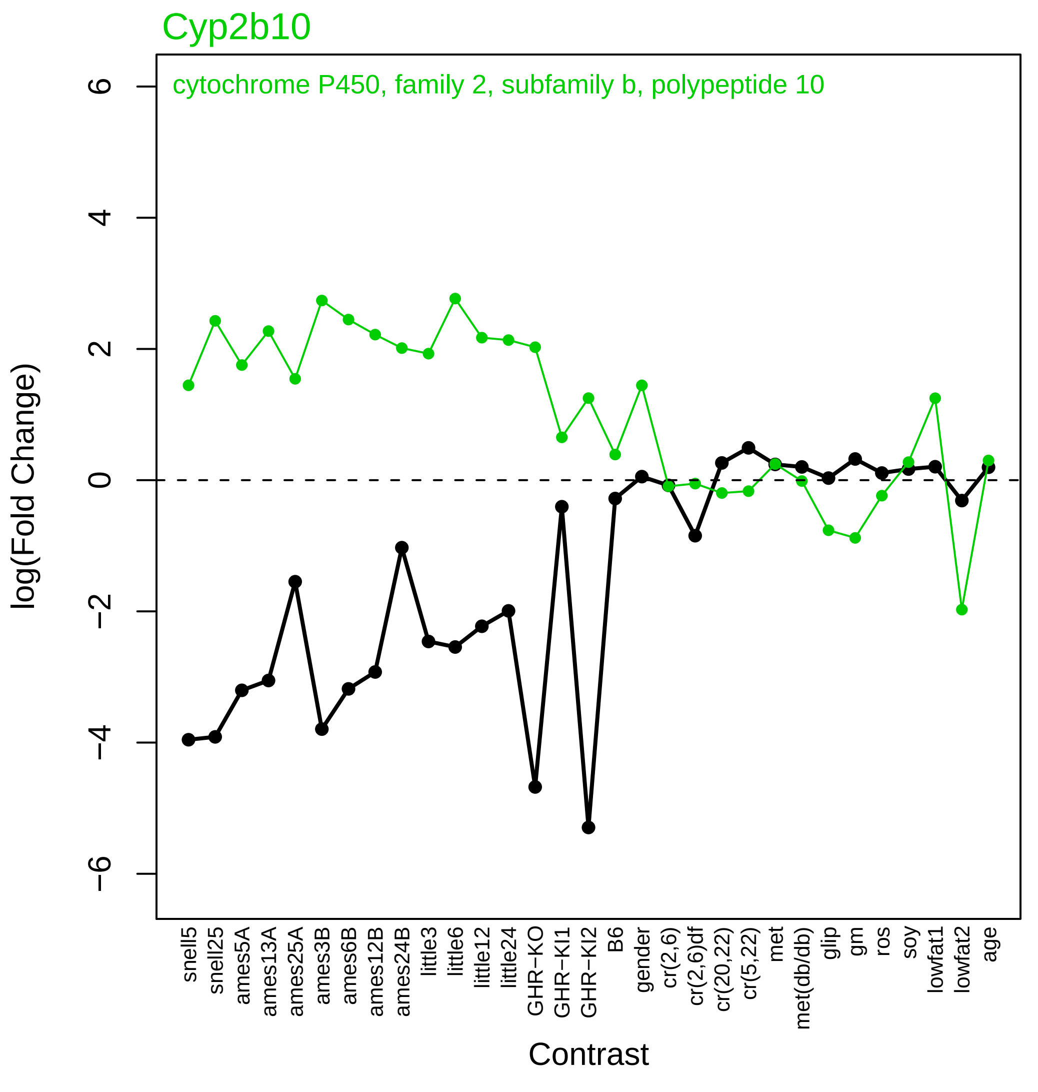

Cyp2b10
cytochrome P450, family 2, subfamily b, polypeptide 10
log(Fold Change)
6
4
2
0
−2
−4
−6
snell5
snell25
ames5A
ames13A
ames25A
ames3B
ames6B
ames12B
ames24B
little3
little6
little12
little24
GHR−KO
GHR−KI1
GHR−KI2
B6
gender
cr(2,6)
cr(2,6)df
cr(20,22)
cr(5,22)
met
met(db/db)
glip
gm
ros
soy
lowfat1
lowfat2
age
Contrast

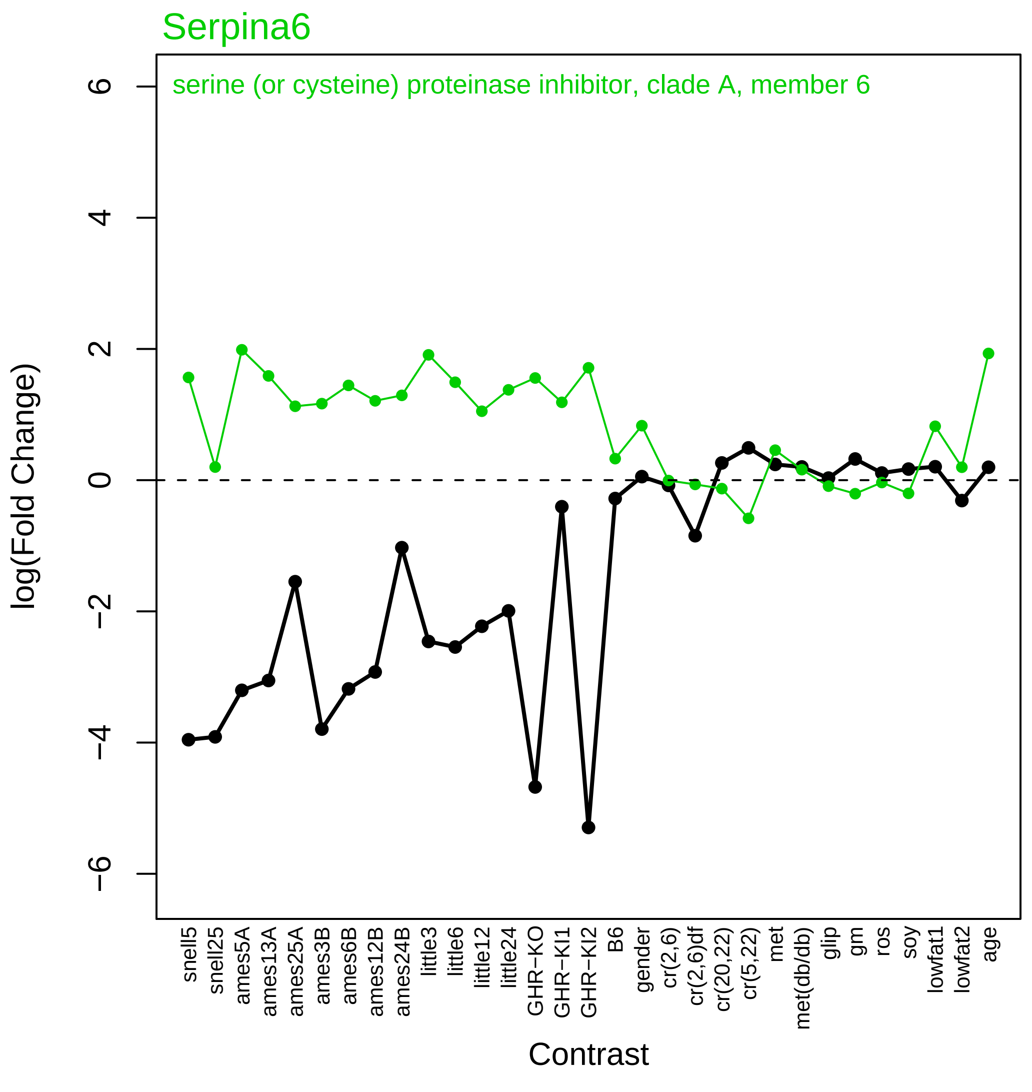

Serpina6
serine (or cysteine) proteinase inhibitor, clade A, member 6
log(Fold Change)
6
4
2
0
−2
−4
−6
snell5
snell25
ames5A
ames13A
ames25A
ames3B
ames6B
ames12B
ames24B
little3
little6
little12
little24
GHR−KO
GHR−KI1
GHR−KI2
B6
gender
cr(2,6)
cr(2,6)df
cr(20,22)
cr(5,22)
met
met(db/db)
glip
gm
ros
soy
lowfat1
lowfat2
age
Contrast

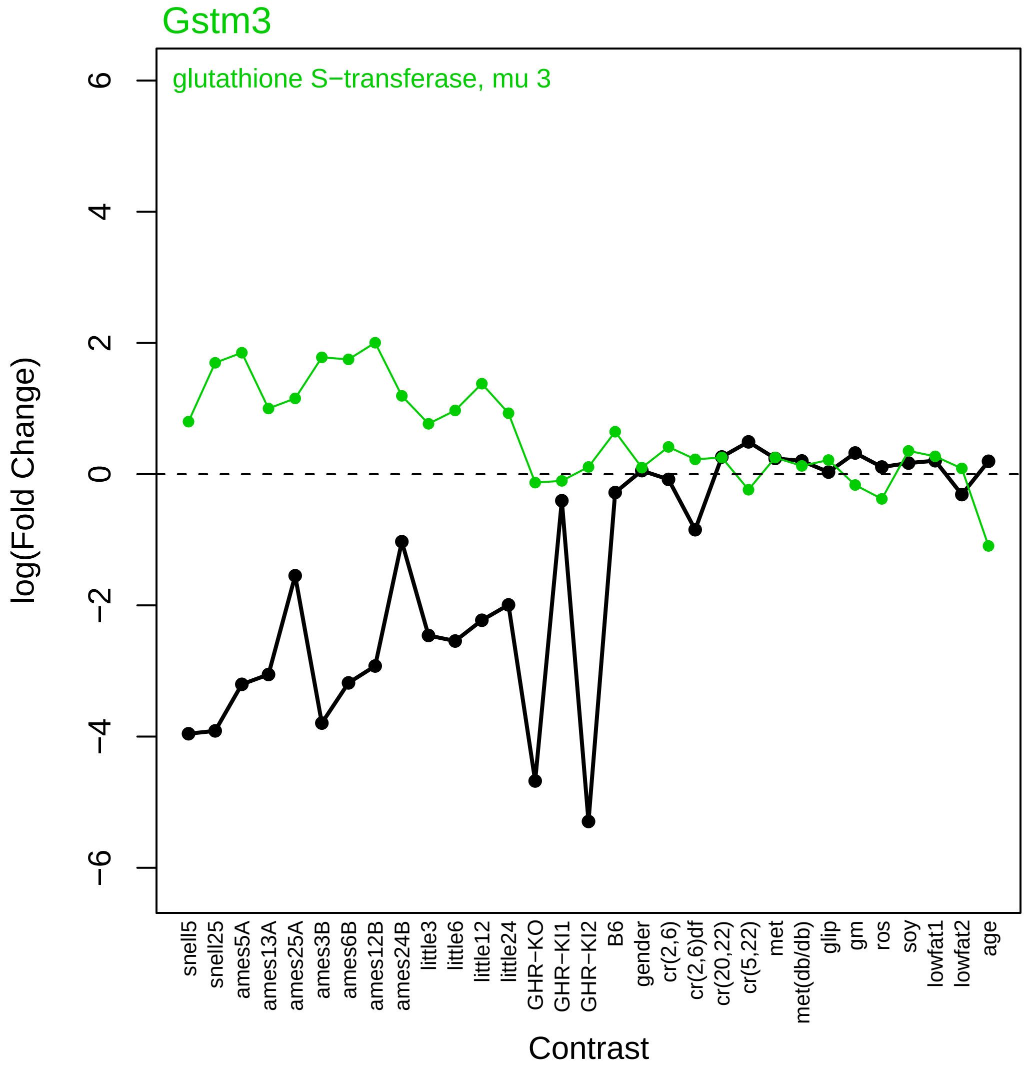
Gstm3
glutathione S−transferase, mu 3
log(Fold Change)
6
4
2
0
−2
−4
−6
snell5
snell25
ames5A
ames13A
ames25A
ames3B
ames6B
ames12B
ames24B
little3
little6
little12
little24
GHR−KO
GHR−KI1
GHR−KI2
B6
gender
cr(2,6)
cr(2,6)df
cr(20,22)
cr(5,22)
met
met(db/db)
glip
gm
ros
soy
lowfat1
lowfat2
age
Contrast

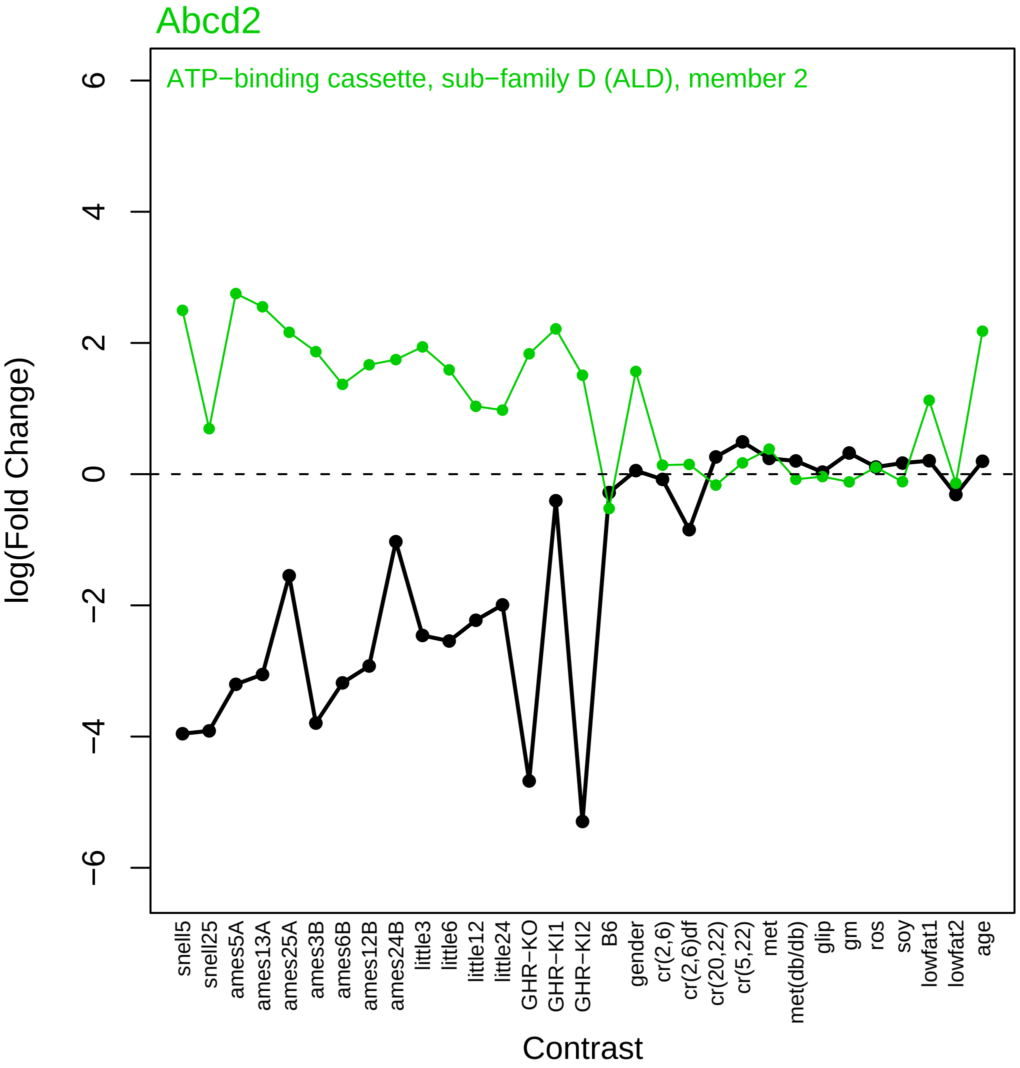

Abcd2
ATP−binding cassette, sub−family D (ALD), member 2
log(Fold Change)
6
4
2
0
−2
−4
−6
snell5
snell25
ames5A
ames13A
ames25A
ames3B
ames6B
ames12B
ames24B
little3
little6
little12
little24
GHR−KO
GHR−KI1
GHR−KI2
B6
gender
cr(2,6)
cr(2,6)df
cr(20,22)
cr(5,22)
met
met(db/db)
glip
gm
ros
soy
lowfat1
lowfat2
age
Contrast

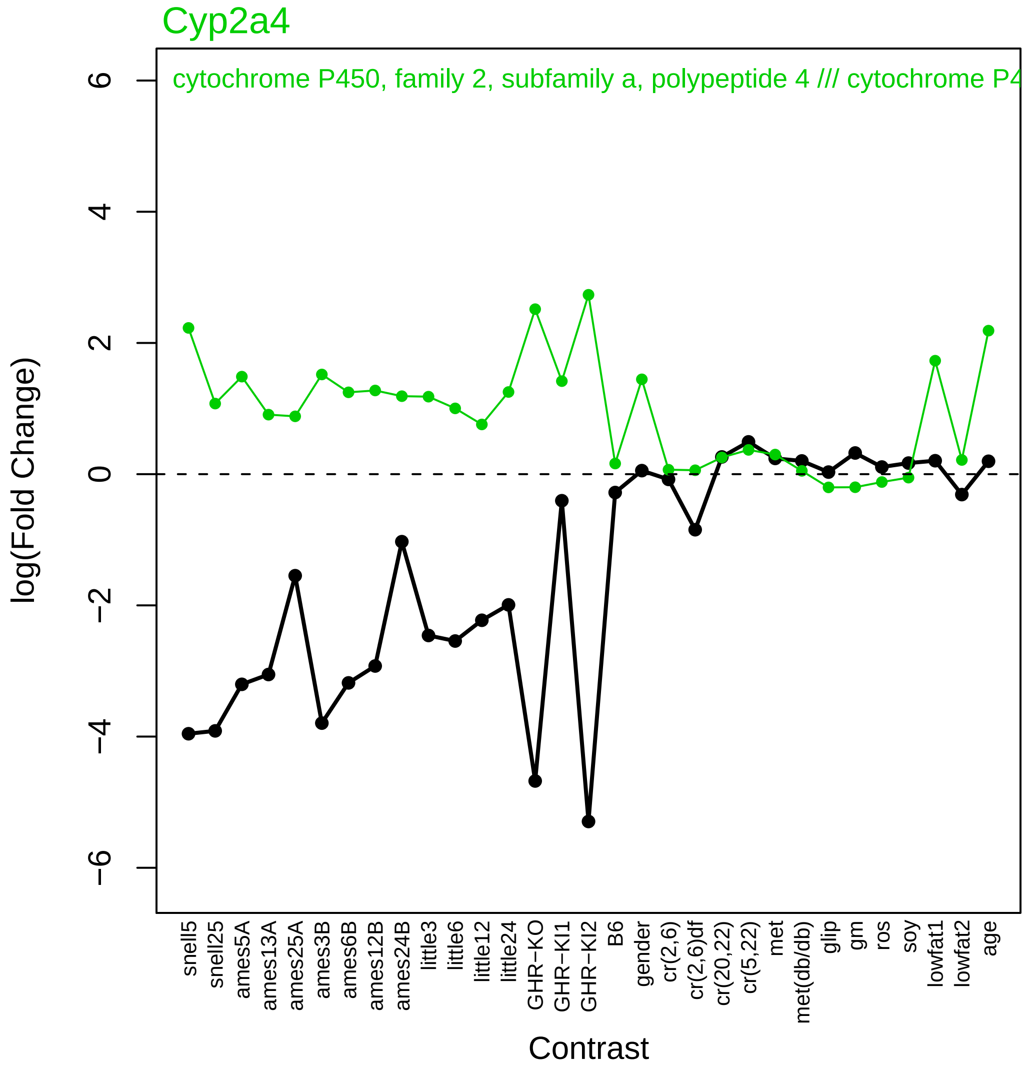
Cyp2a4
cytochrome P450, family 2, subfamily a, polypeptide 4 /// cytochrome P4
log(Fold Change)
6
4
2
0
−2
−4
−6
snell5
snell25
ames5A
ames13A
ames25A
ames3B
ames6B
ames12B
ames24B
little3
little6
little12
little24
GHR−KO
GHR−KI1
GHR−KI2
B6
gender
cr(2,6)
cr(2,6)df
cr(20,22)
cr(5,22)
met
met(db/db)
glip
gm
ros
soy
lowfat1
lowfat2
age
Contrast

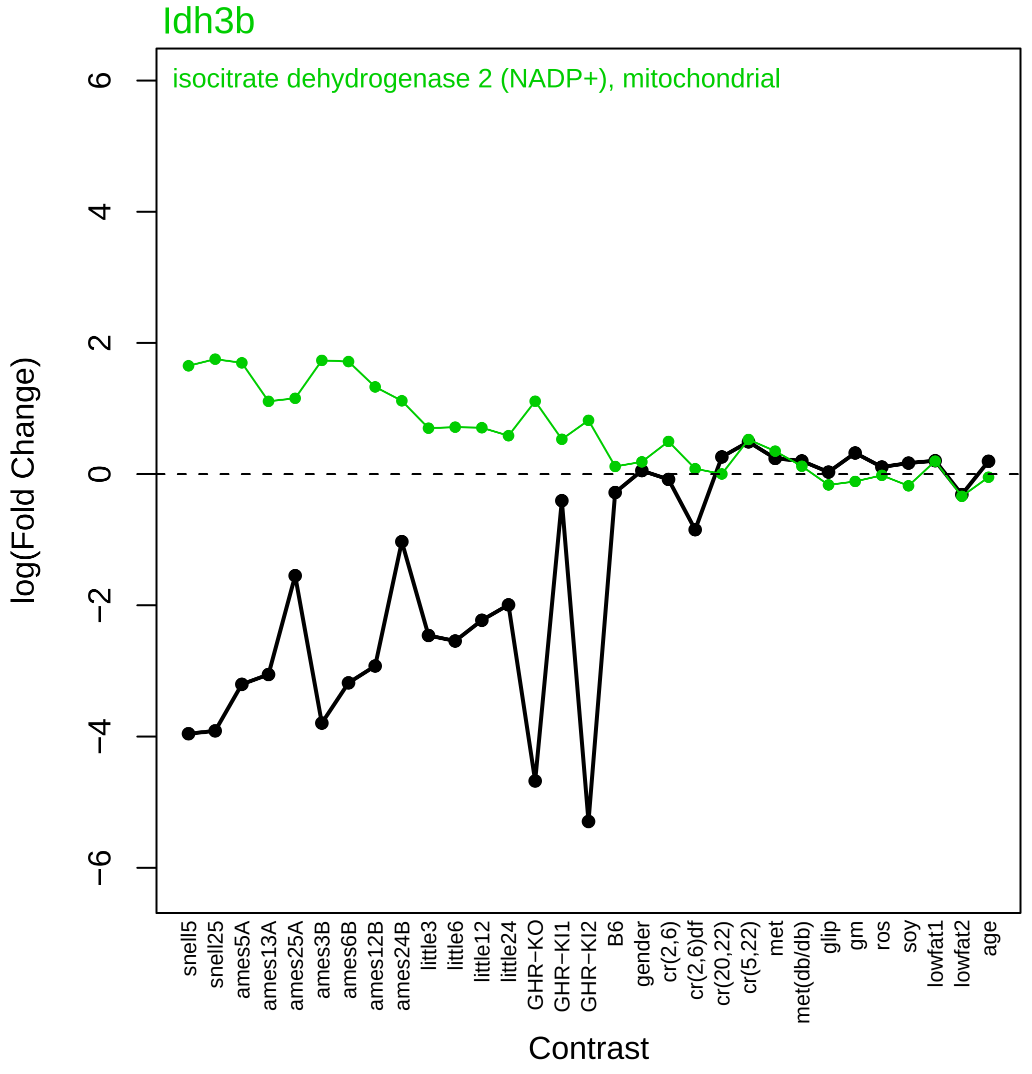

Idh3b
isocitrate dehydrogenase 2 (NADP+), mitochondrial
log(Fold Change)
6
4
2
0
−2
−4
−6
snell5
snell25
ames5A
ames13A
ames25A
ames3B
ames6B
ames12B
ames24B
little3
little6
little12
little24
GHR−KO
GHR−KI1
GHR−KI2
B6
gender
cr(2,6)
cr(2,6)df
cr(20,22)
cr(5,22)
met
met(db/db)
glip
gm
ros
soy
lowfat1
lowfat2
age
Contrast

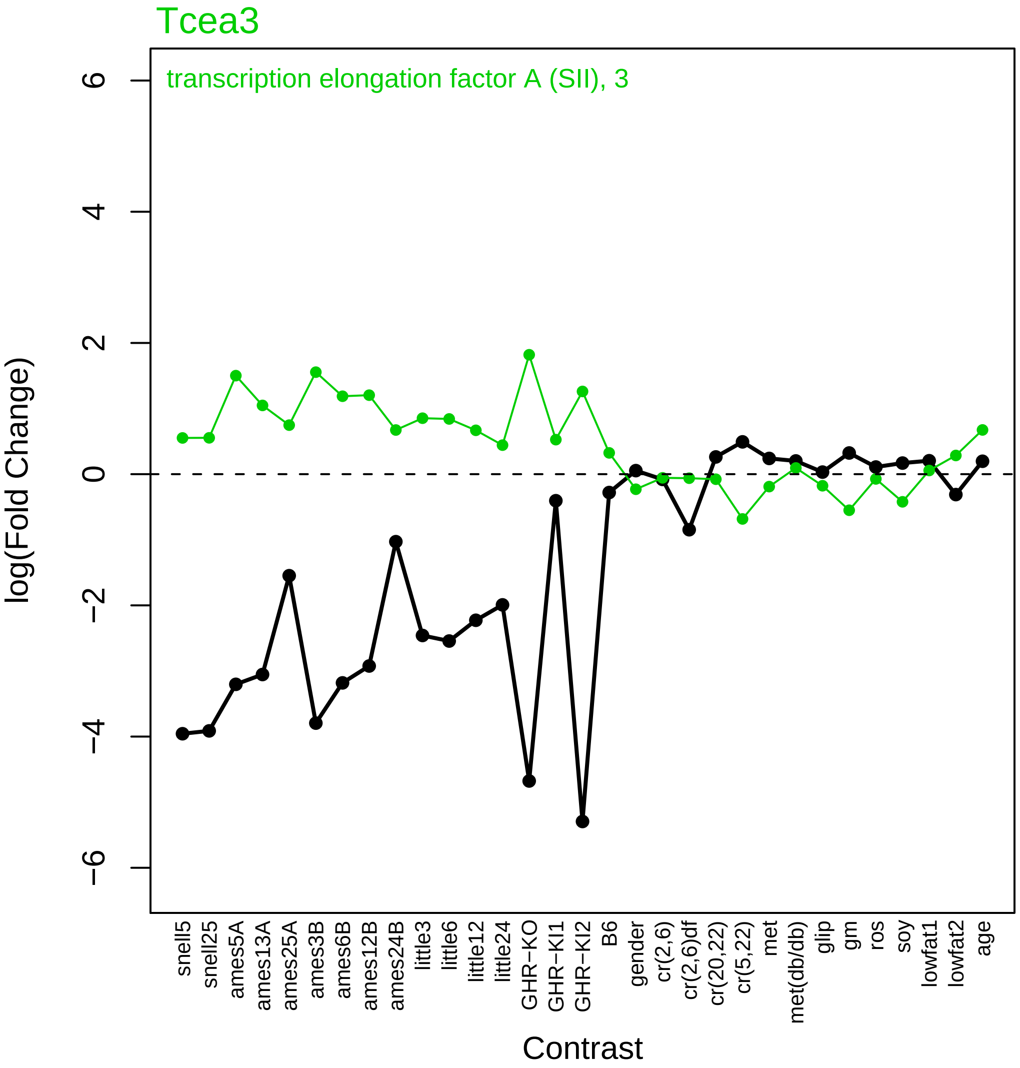

Tcea3
transcription elongation factor A (SII), 3
log(Fold Change)
6
4
2
0
−2
−4
−6
Contrast
snell5
snell25
ames5A
ames13A
ames25A
ames3B
ames6B
ames12B
ames24B
little3
little6
little12
little24
GHR−KO
GHR−KI1
GHR−KI2
B6
gender
cr(2,6)
cr(2,6)df
cr(20,22)
cr(5,22)
met
met(db/db)
glip
gm
ros
soy
lowfat1
lowfat2
age

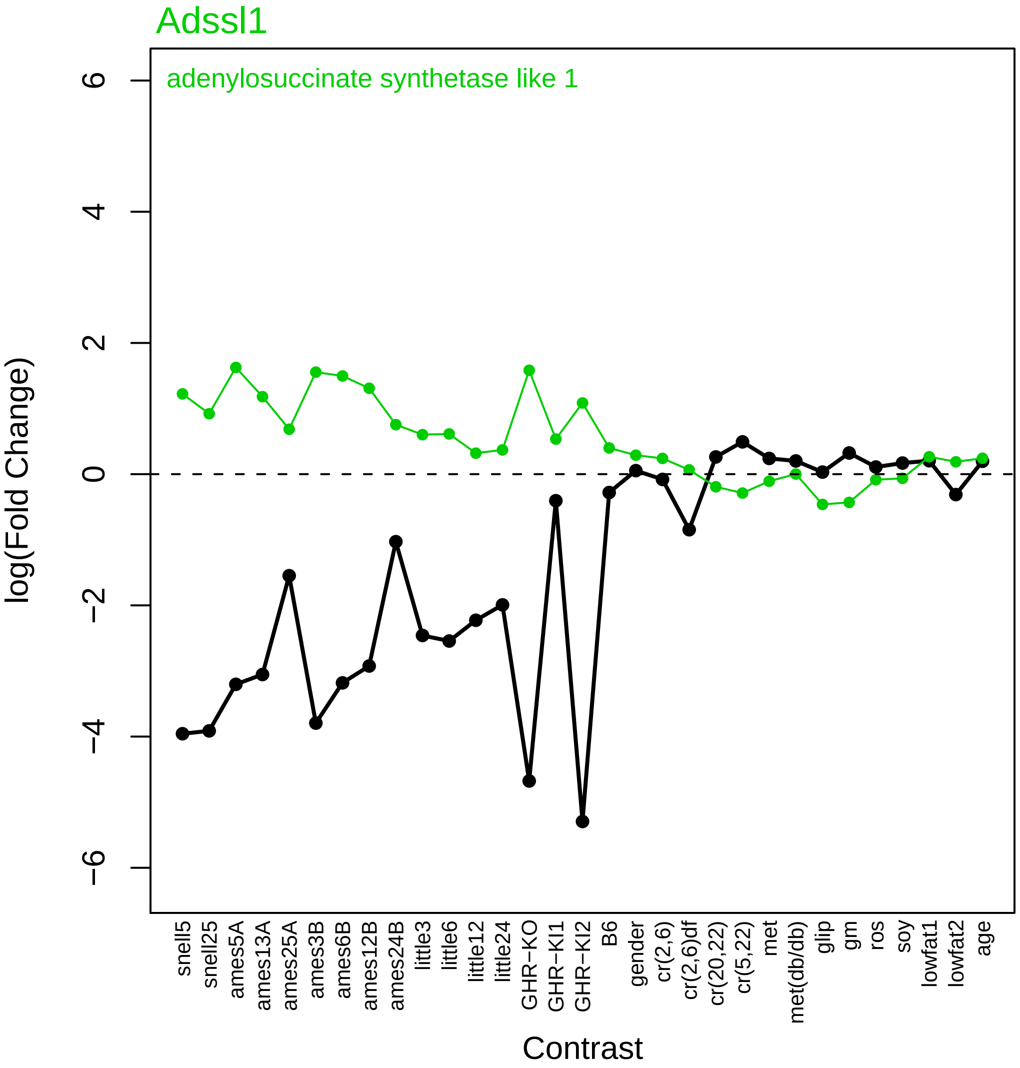

Adssl1
adenylosuccinate synthetase like 1
log(Fold Change)
6
4
2
0
−2
−4
−6
snell5
snell25
ames5A
ames13A
ames25A
ames3B
ames6B
ames12B
ames24B
little3
little6
little12
little24
GHR−KO
GHR−KI1
GHR−KI2
B6
gender
cr(2,6)
cr(2,6)df
cr(20,22)
cr(5,22)
met
met(db/db)
glip
gm
ros
soy
lowfat1
lowfat2
age
Contrast

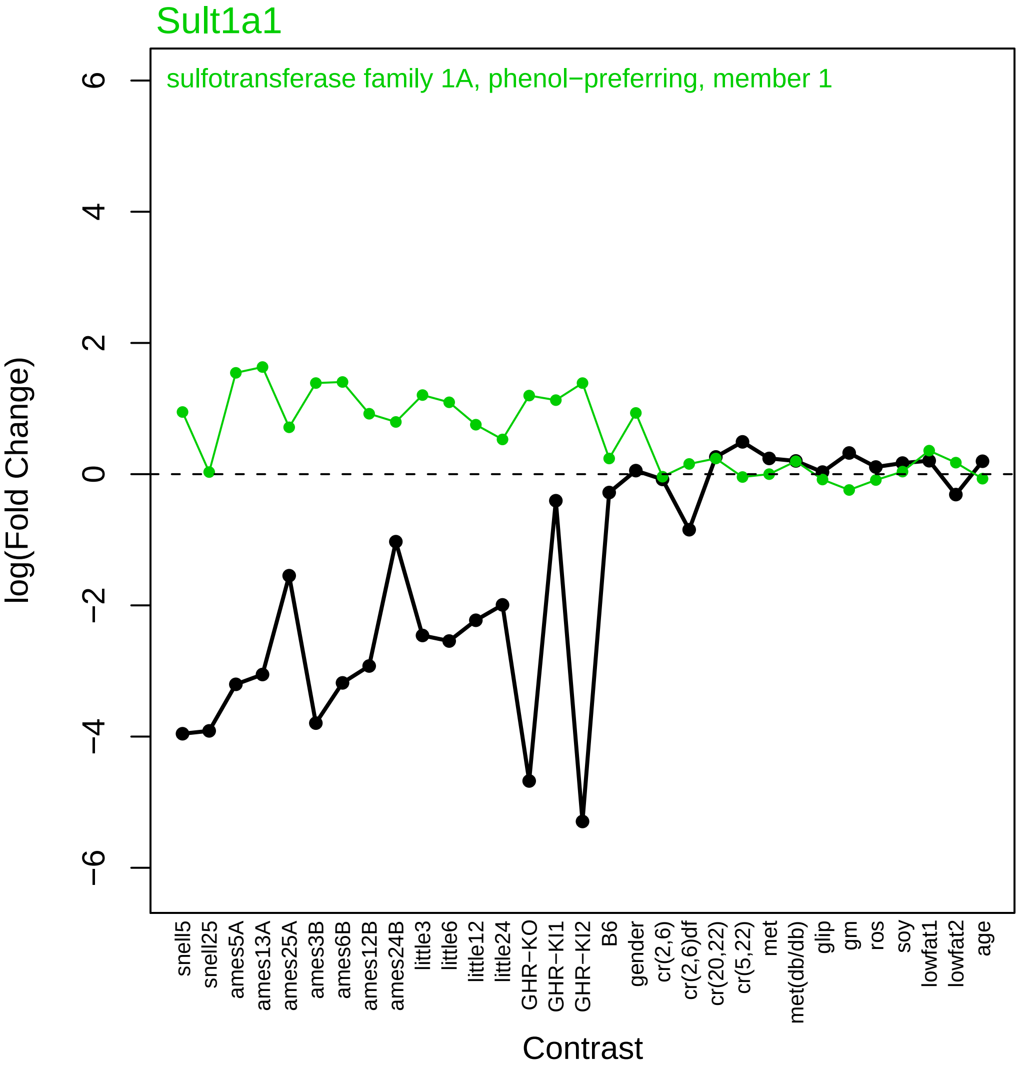

Sult1a1
sulfotransferase family 1A, phenol−preferring, member 1
log(Fold Change)
6
4
2
0
−2
−4
−6
snell5
snell25
ames5A
ames13A
ames25A
ames3B
ames6B
ames12B
ames24B
little3
little6
little12
little24
GHR−KO
GHR−KI1
GHR−KI2
B6
gender
cr(2,6)
cr(2,6)df
cr(20,22)
cr(5,22)
met
met(db/db)
glip
gm
ros
soy
lowfat1
lowfat2
age
Contrast

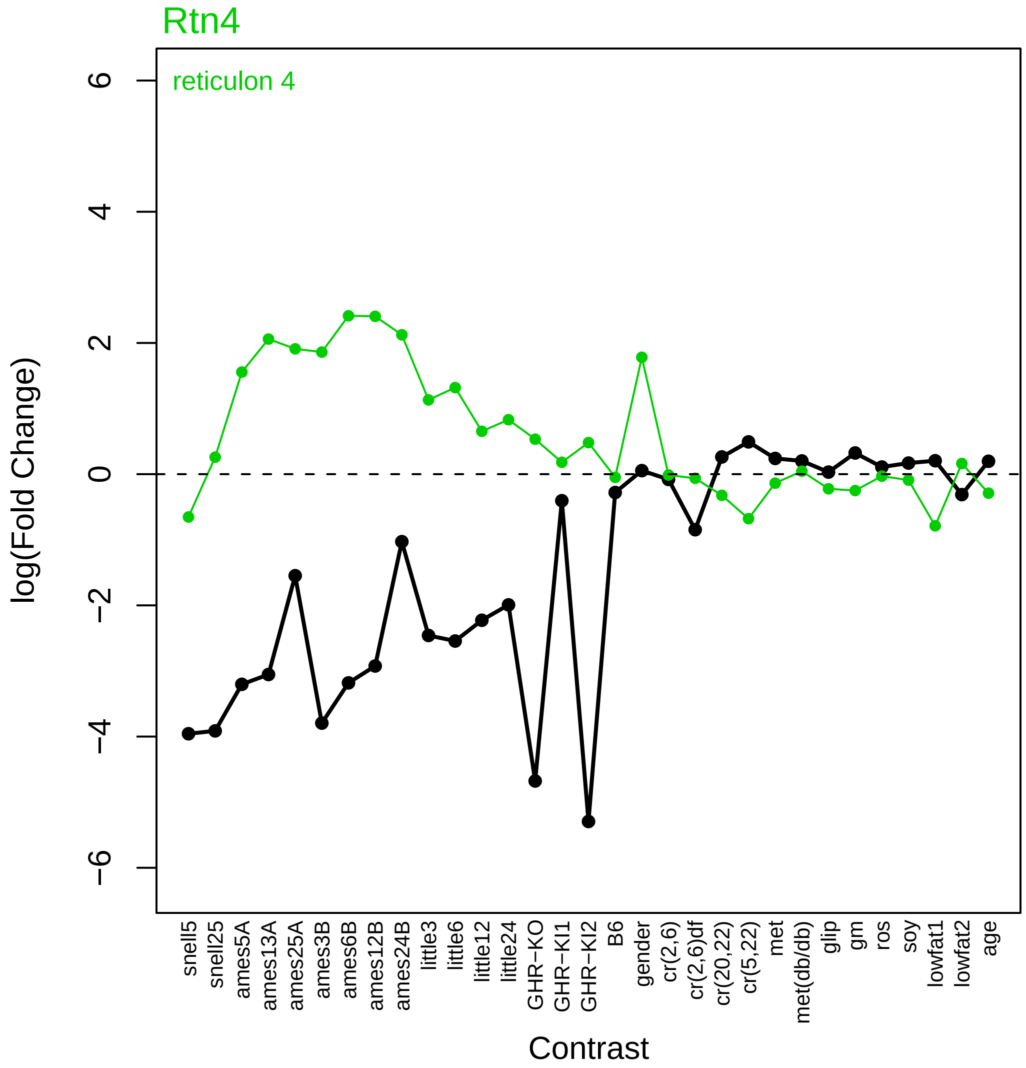

Rtn4
reticulon 4
log(Fold Change)
Contrast
6
4
2
0
−2
−4
−6
snell5
snell25
ames5A
ames13A
ames25A
ames3B
ames6B
ames12B
ames24B
little3
little6
little12
little24
GHR−KO
GHR−KI1
GHR−KI2
B6
gender
cr(2,6)
cr(2,6)df
cr(20,22)
cr(5,22)
met
met(db/db)
glip
gm
ros
soy
lowfat1
lowfat2
age

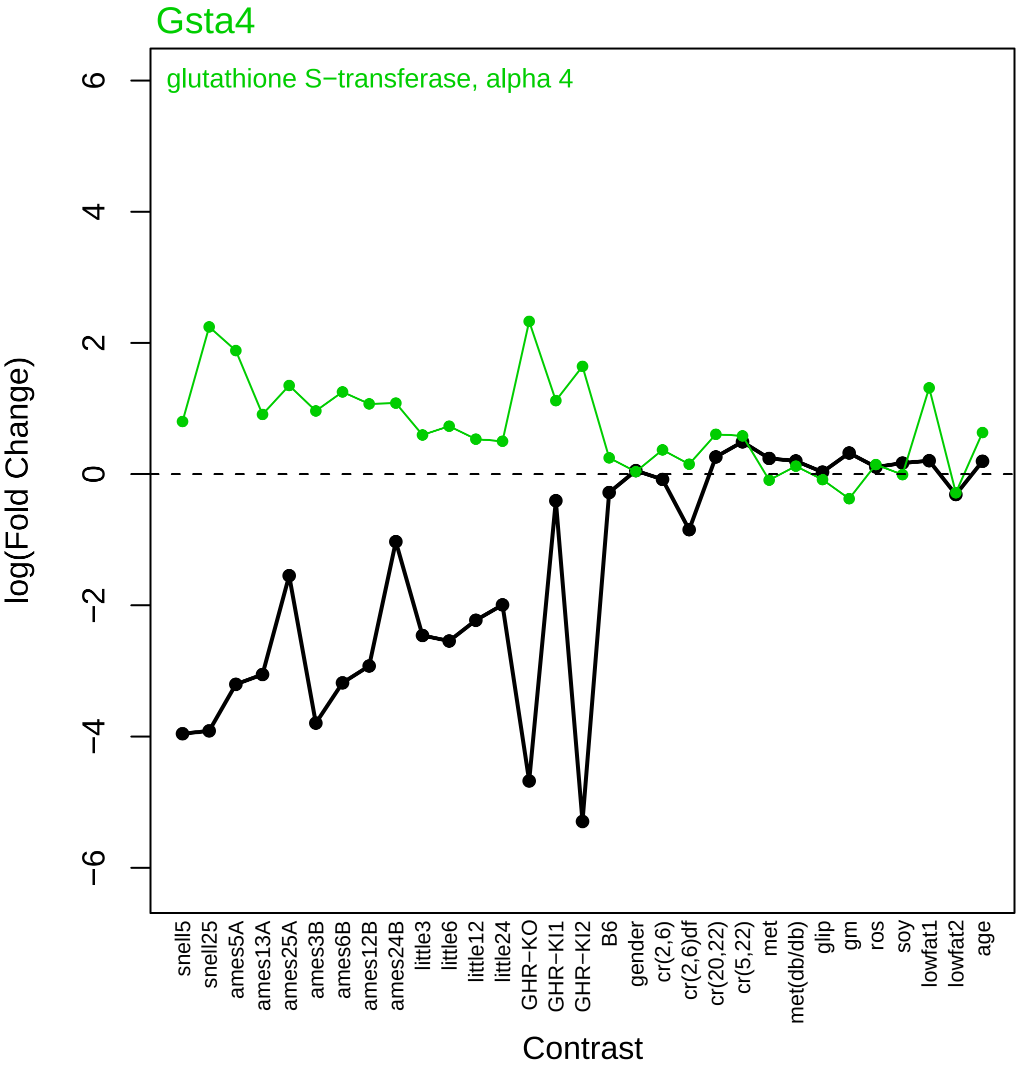

Gsta4
glutathione S−transferase, alpha 4
log(Fold Change)
Contrast
6
4
2
0
−2
−4
−6
snell5
snell25
ames5A
ames13A
ames25A
ames3B
ames6B
ames12B
ames24B
little3
little6
little12
little24
GHR−KO
GHR−KI1
GHR−KI2
B6
gender
cr(2,6)
cr(2,6)df
cr(20,22)
cr(5,22)
met
met(db/db)
glip
gm
ros
soy
lowfat1
lowfat2
age

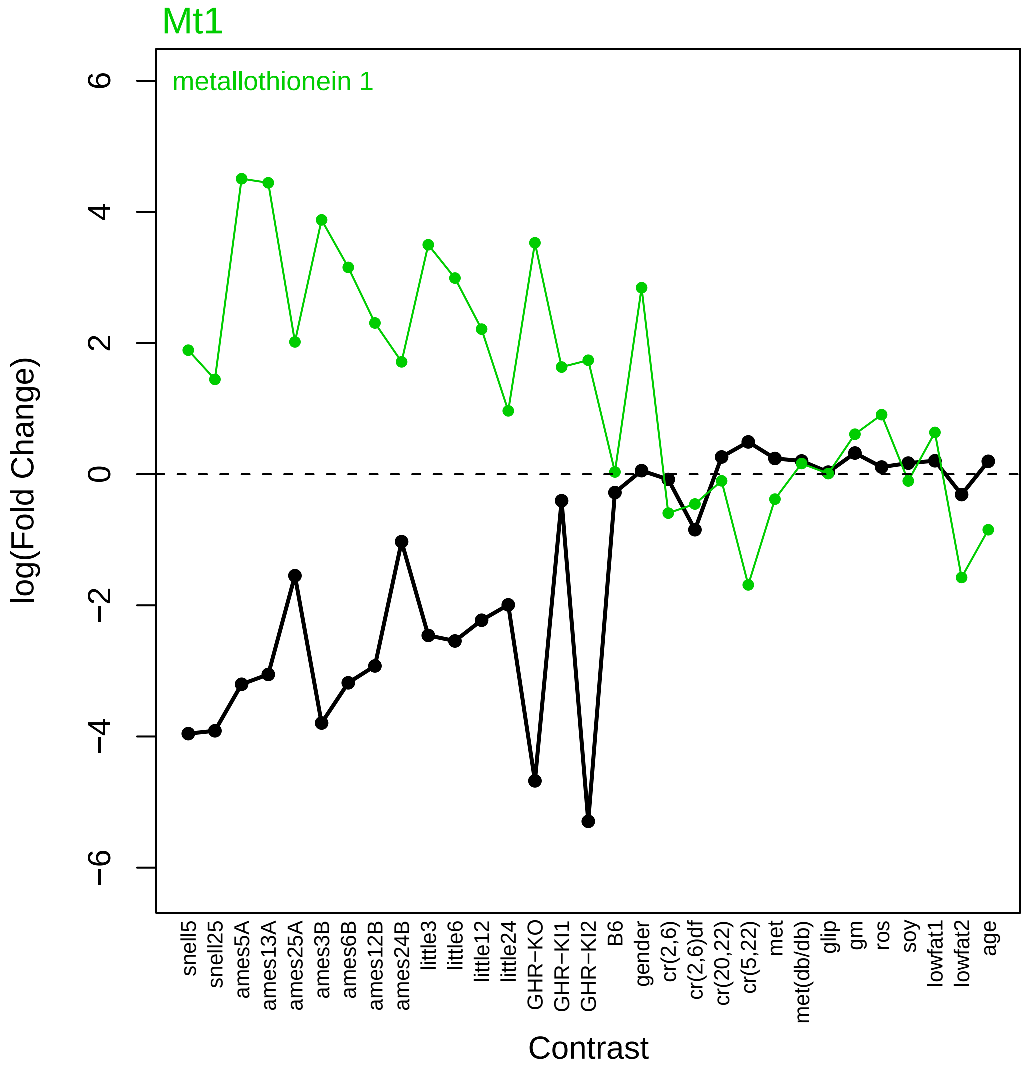
Mt1
metallothionein 1
log(Fold Change)
6
4
2
0
−2
−4
−6
snell5
snell25
ames5A
ames13A
ames25A
ames3B
ames6B
ames12B
ames24B
little3
little6
little12
little24
GHR−KO
GHR−KI1
GHR−KI2
B6
gender
cr(2,6)
cr(2,6)df
cr(20,22)
cr(5,22)
met
met(db/db)
glip
gm
ros
soy
lowfat1
lowfat2
age
Contrast

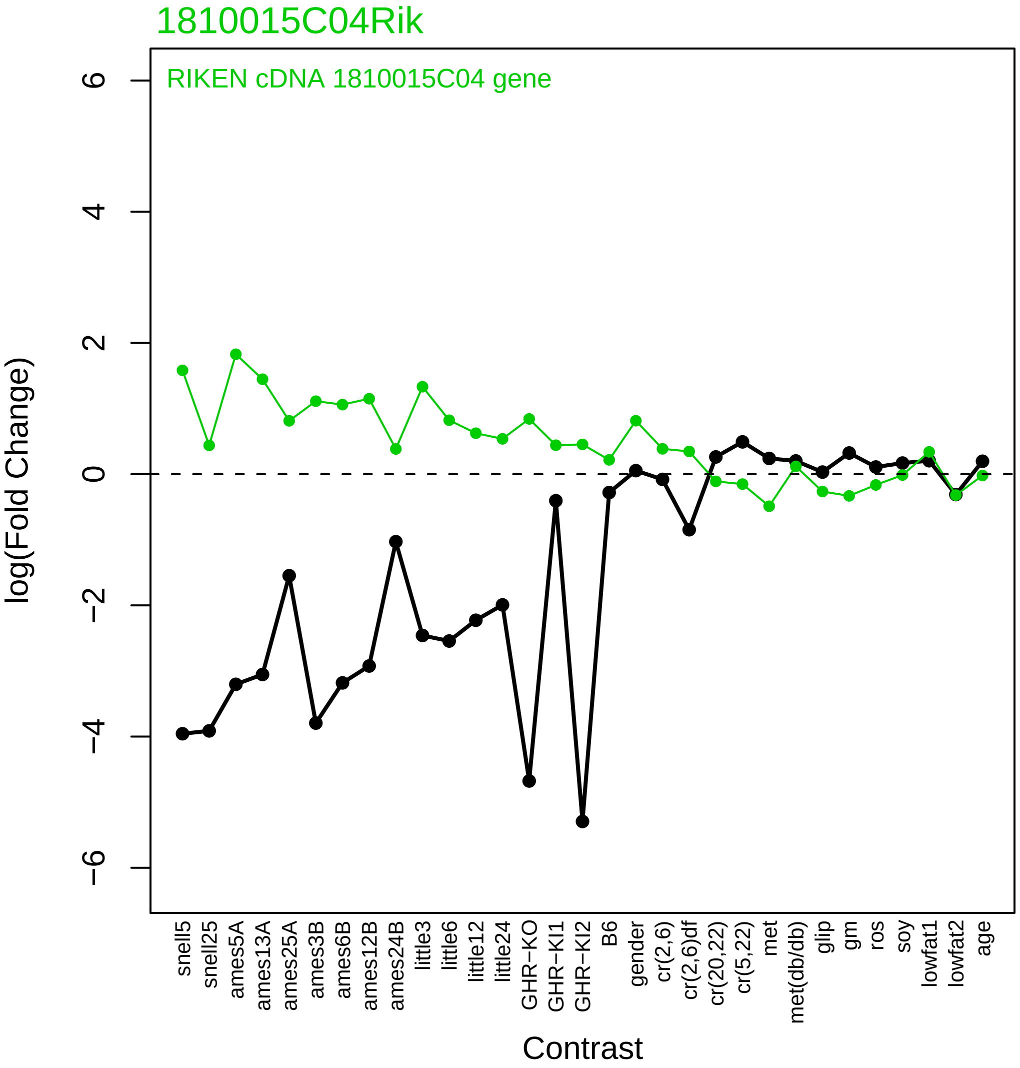
1810015C04Rik
RIKEN cDNA 1810015C04 gene
log(Fold Change)
6
4
2
0
−2
−4
−6
snell5
snell25
ames5A
ames13A
ames25A
ames3B
ames6B
ames12B
ames24B
little3
little6
little12
little24
GHR−KO
GHR−KI1
GHR−KI2
B6
gender
cr(2,6)
cr(2,6)df
cr(20,22)
cr(5,22)
met
met(db/db)
glip
gm
ros
soy
lowfat1
lowfat2
age
Contrast

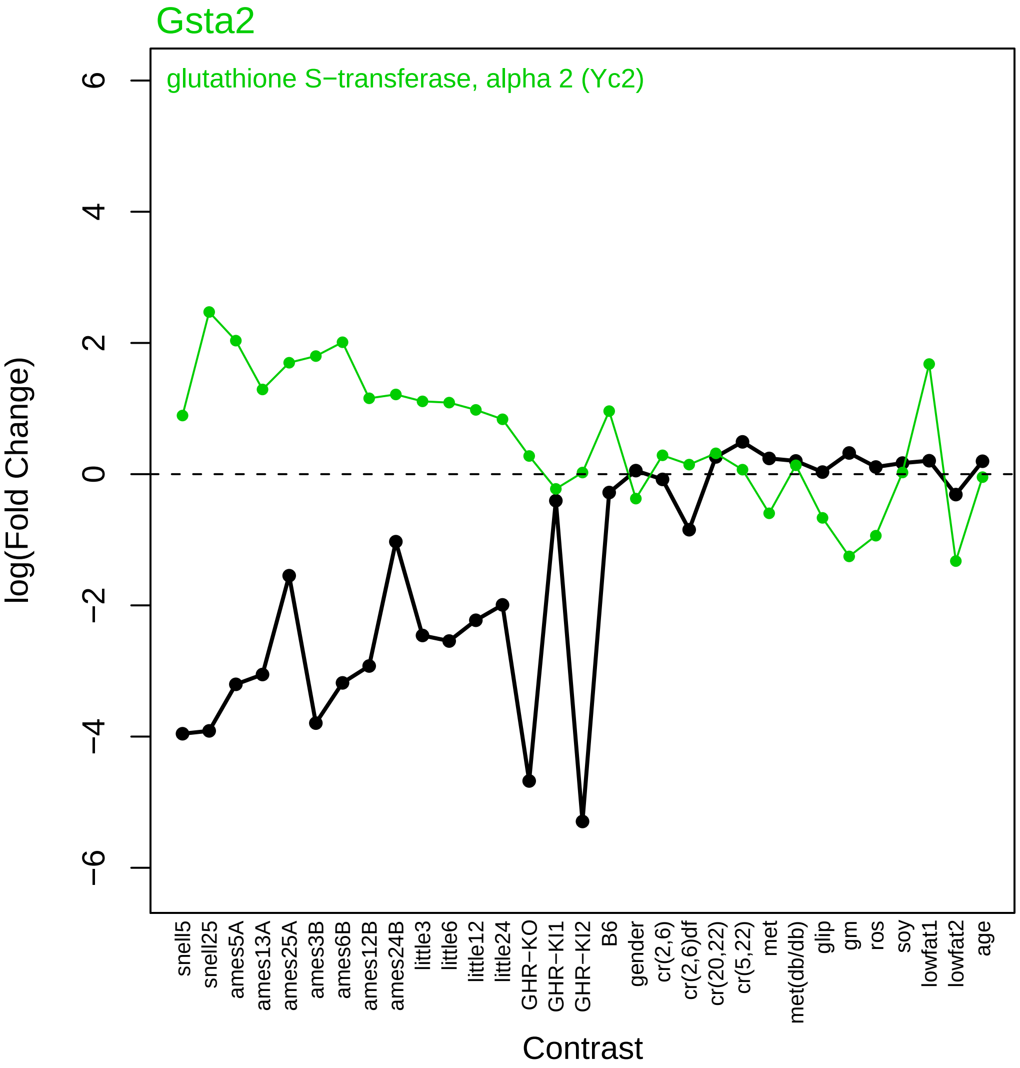

Gsta2
glutathione S−transferase, alpha 2 (Yc2)
log(Fold Change)
6
4
2
0
−2
−4
−6
Contrast
snell5
snell25
ames5A
ames13A
ames25A
ames3B
ames6B
ames12B
ames24B
little3
little6
little12
little24
GHR−KO
GHR−KI1
GHR−KI2
B6
gender
cr(2,6)
cr(2,6)df
cr(20,22)
cr(5,22)
met
met(db/db)
glip
gm
ros
soy
lowfat1
lowfat2
age

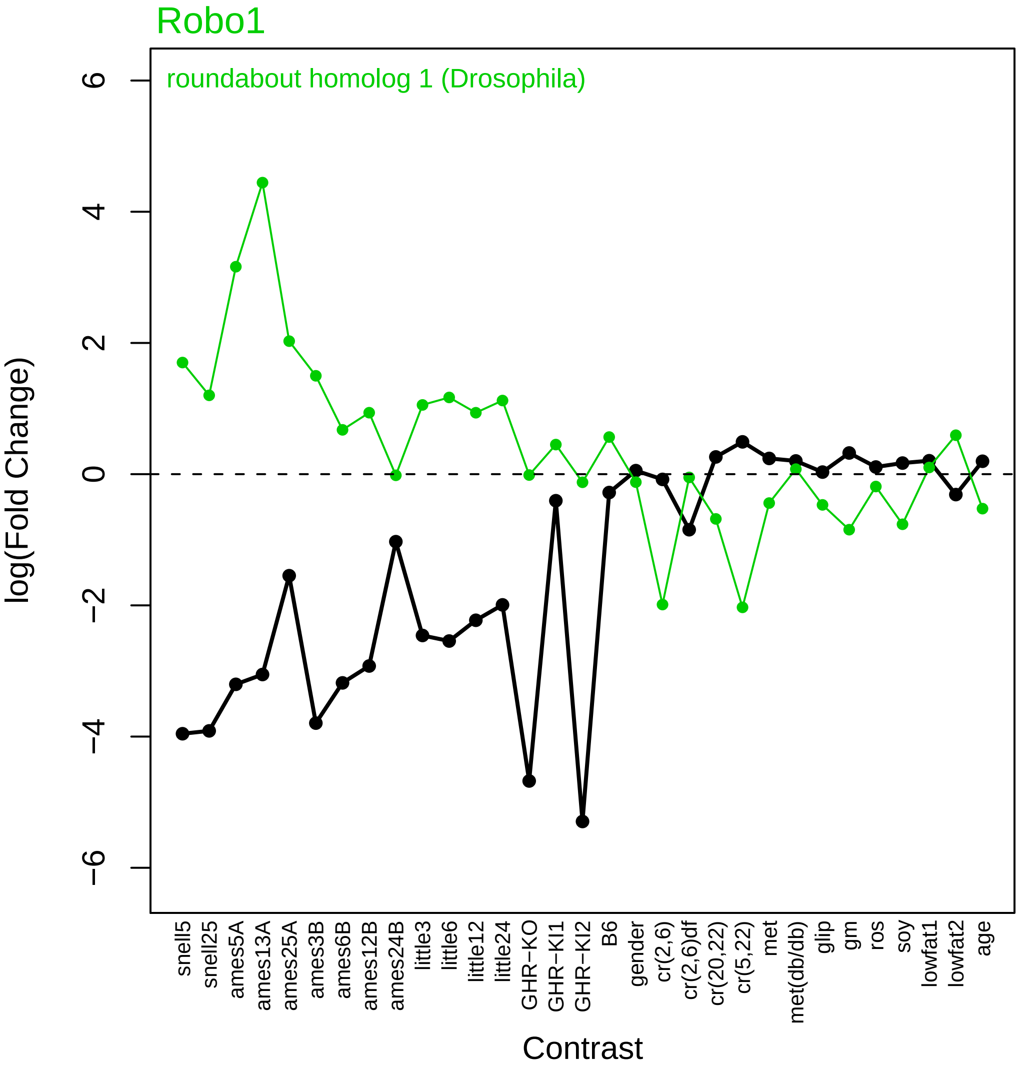

Robo1
roundabout homolog 1 (Drosophila)
log(Fold Change)
6
4
2
0
−2
−4
−6
snell5
snell25
ames5A
ames13A
ames25A
ames3B
ames6B
ames12B
ames24B
little3
little6
little12
little24
GHR−KO
GHR−KI1
GHR−KI2
B6
gender
cr(2,6)
cr(2,6)df
cr(20,22)
cr(5,22)
met
met(db/db)
glip
gm
ros
soy
lowfat1
lowfat2
age
Contrast

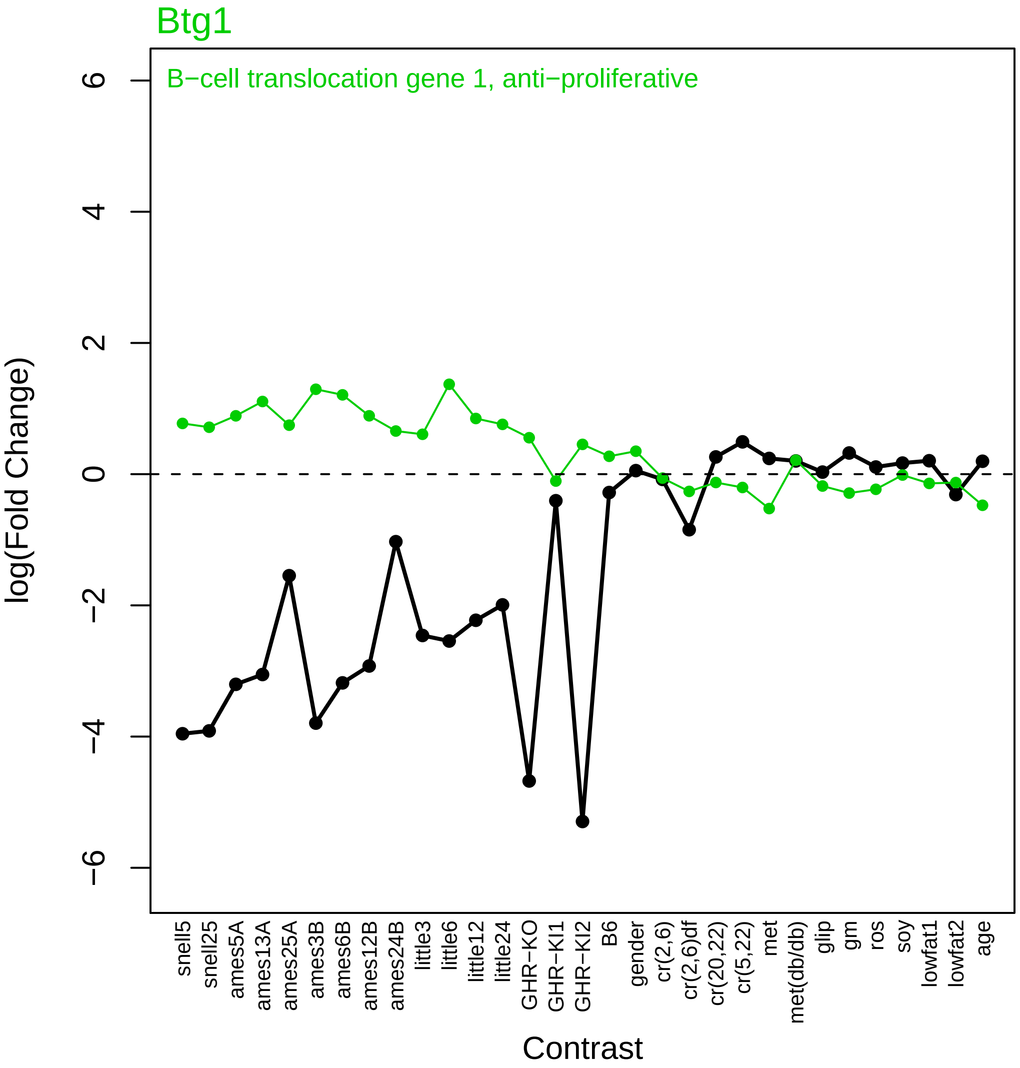
Btg1
B−cell translocation gene 1, anti−proliferative
log(Fold Change)
6
4
2
0
−2
−4
−6
snell5
snell25
ames5A
ames13A
ames25A
ames3B
ames6B
ames12B
ames24B
little3
little6
little12
little24
GHR−KO
GHR−KI1
GHR−KI2
B6
gender
cr(2,6)
cr(2,6)df
cr(20,22)
cr(5,22)
met
met(db/db)
glip
gm
ros
soy
lowfat1
lowfat2
age
Contrast

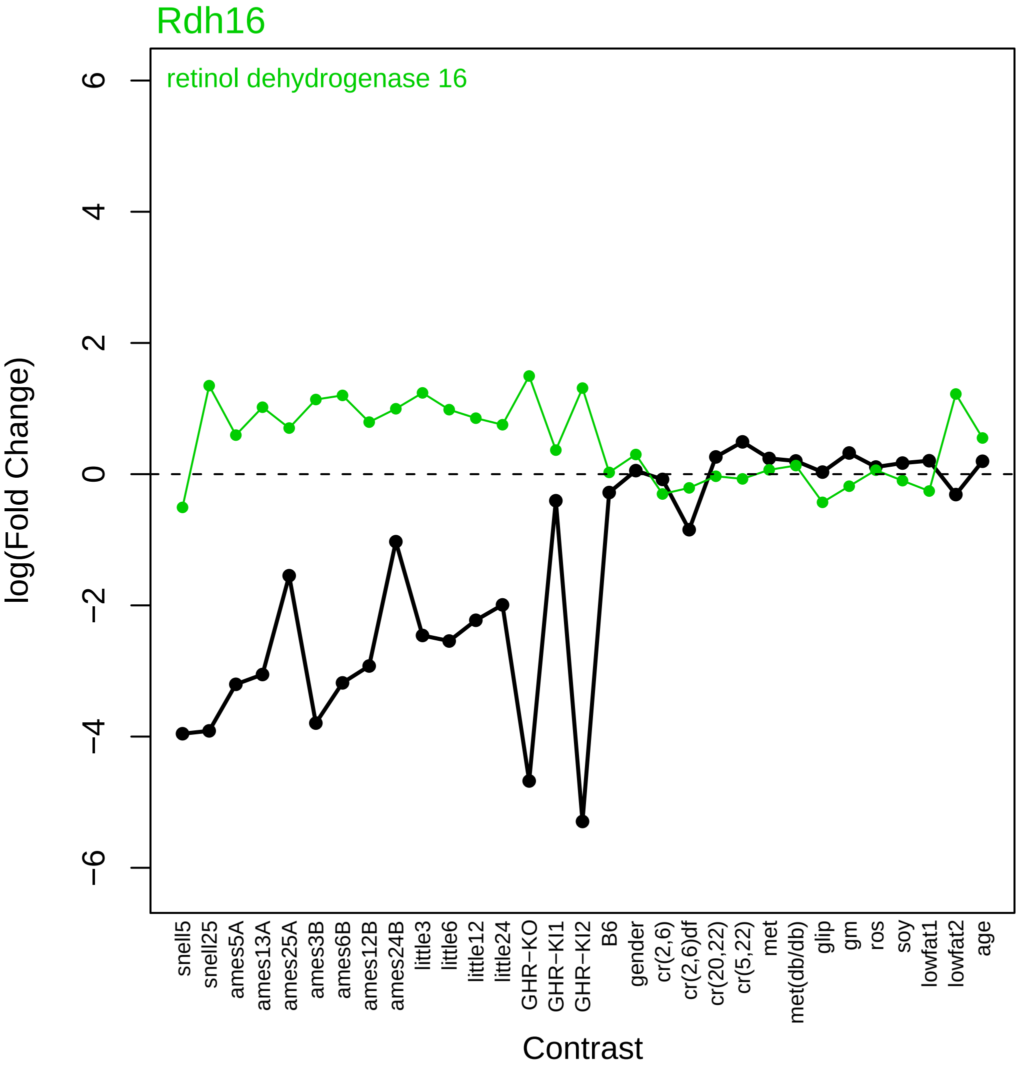
Rdh16
retinol dehydrogenase 16
log(Fold Change)
Contrast
6
4
2
0
−2
−4
−6
snell5
snell25
ames5A
ames13A
ames25A
ames3B
ames6B
ames12B
ames24B
little3
little6
little12
little24
GHR−KO
GHR−KI1
GHR−KI2
B6
gender
cr(2,6)
cr(2,6)df
cr(20,22)
cr(5,22)
met
met(db/db)
glip
gm
ros
soy
lowfat1
lowfat2
age

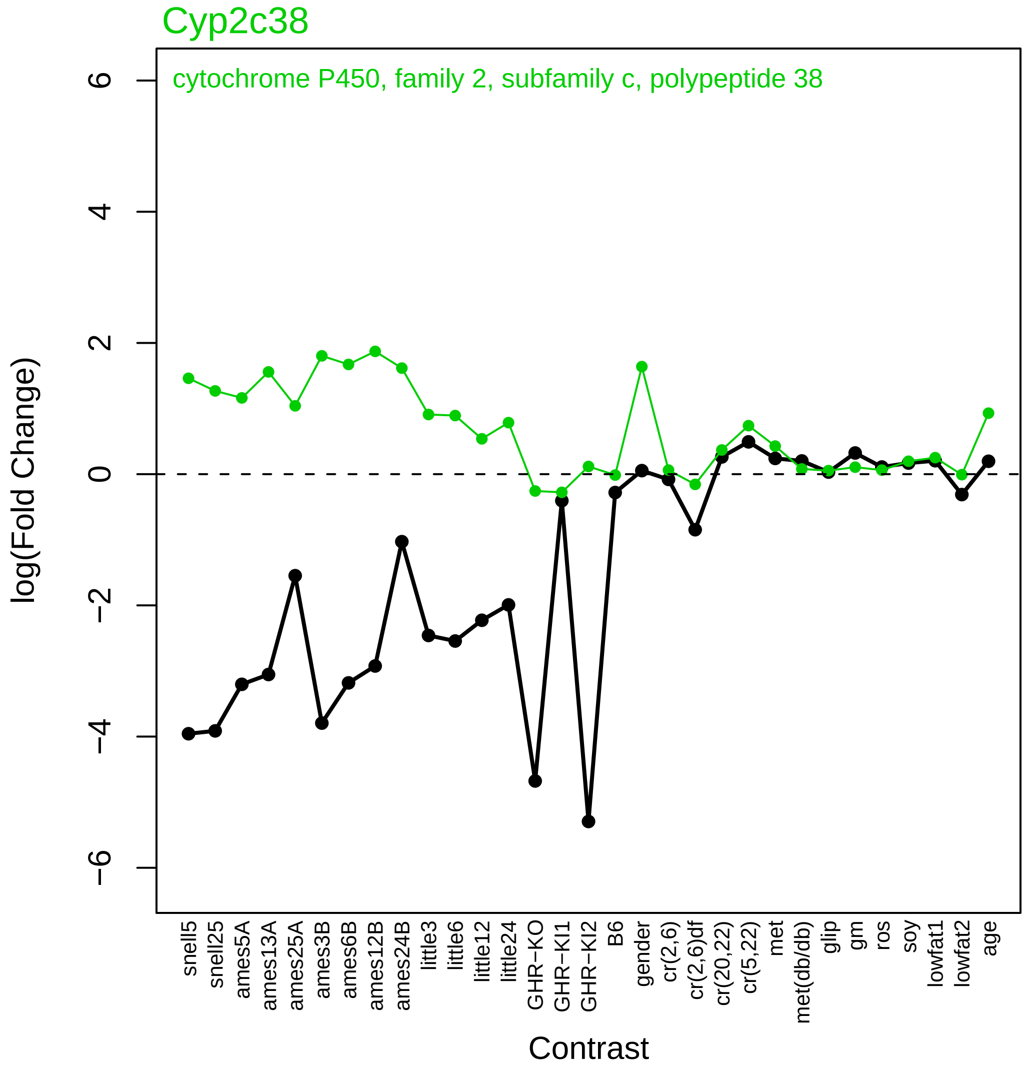
Cyp2c38
cytochrome P450, family 2, subfamily c, polypeptide 38
log(Fold Change)
6
4
2
0
−2
−4
−6
Contrast
snell5
snell25
ames5A
ames13A
ames25A
ames3B
ames6B
ames12B
ames24B
little3
little6
little12
little24
GHR−KO
GHR−KI1
GHR−KI2
B6
gender
cr(2,6)
cr(2,6)df
cr(20,22)
cr(5,22)
met
met(db/db)
glip
gm
ros
soy
lowfat1
lowfat2
age

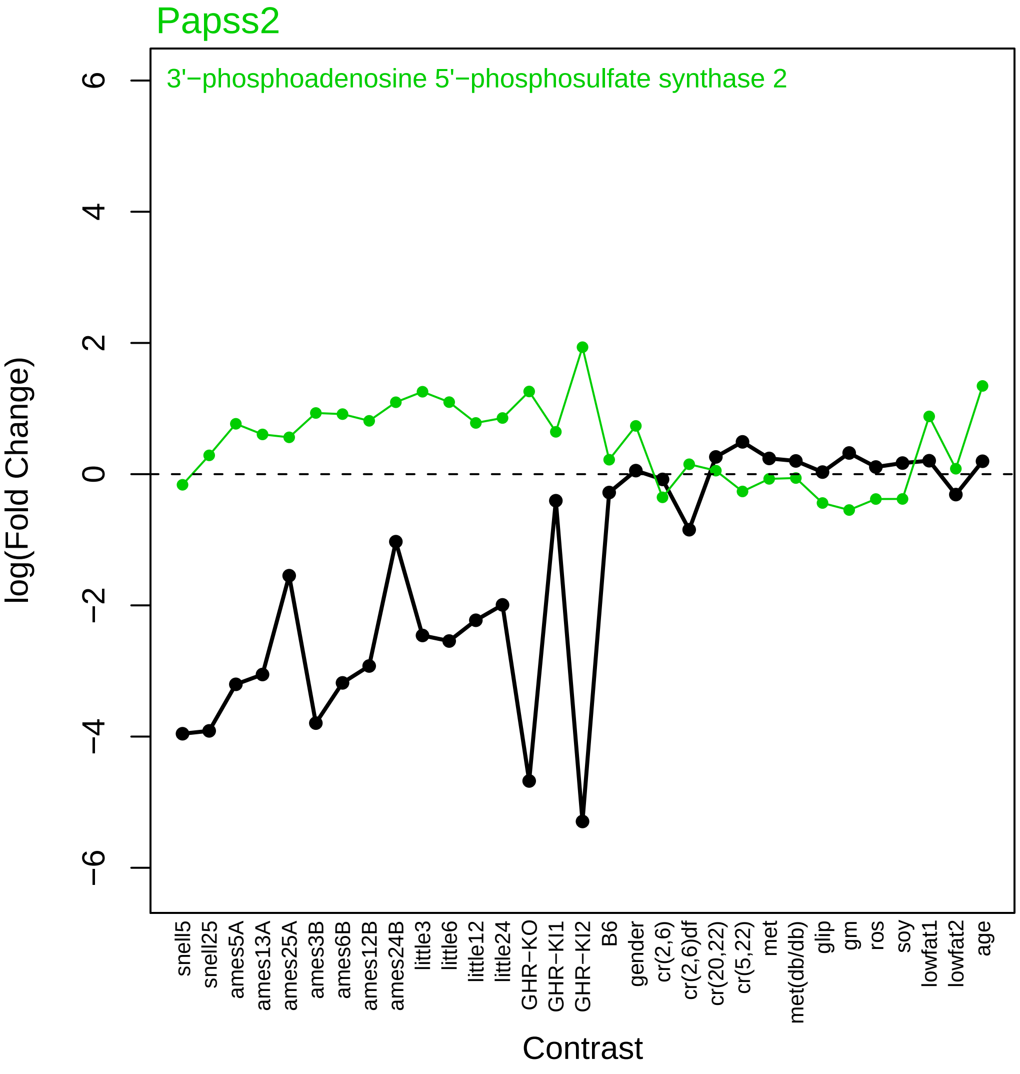

Papss2
3'−phosphoadenosine 5'−phosphosulfate synthase 2
log(Fold Change)
6
4
2
0
−2
−4
−6
snell5
snell25
ames5A
ames13A
ames25A
ames3B
ames6B
ames12B
ames24B
little3
little6
little12
little24
GHR−KO
GHR−KI1
GHR−KI2
B6
gender
cr(2,6)
cr(2,6)df
cr(20,22)
cr(5,22)
met
met(db/db)
glip
gm
ros
soy
lowfat1
lowfat2
age
Contrast

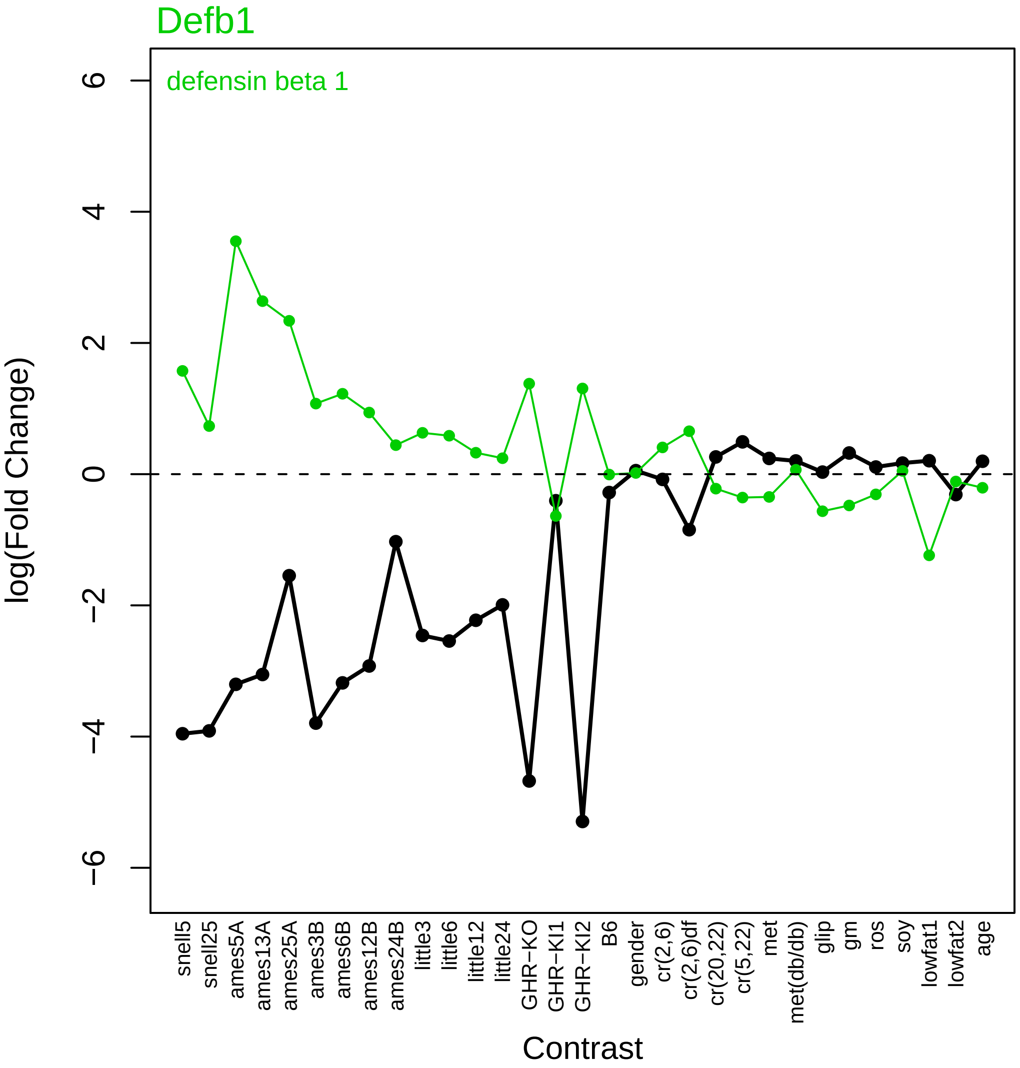
Defb1
defensin beta 1
log(Fold Change)
6
4
2
0
−2
−4
−6
Contrast
snell5
snell25
ames5A
ames13A
ames25A
ames3B
ames6B
ames12B
ames24B
little3
little6
little12
little24
GHR−KO
GHR−KI1
GHR−KI2
B6
gender
cr(2,6)
cr(2,6)df
cr(20,22)
cr(5,22)
met
met(db/db)
glip
gm
ros
soy
lowfat1
lowfat2
age

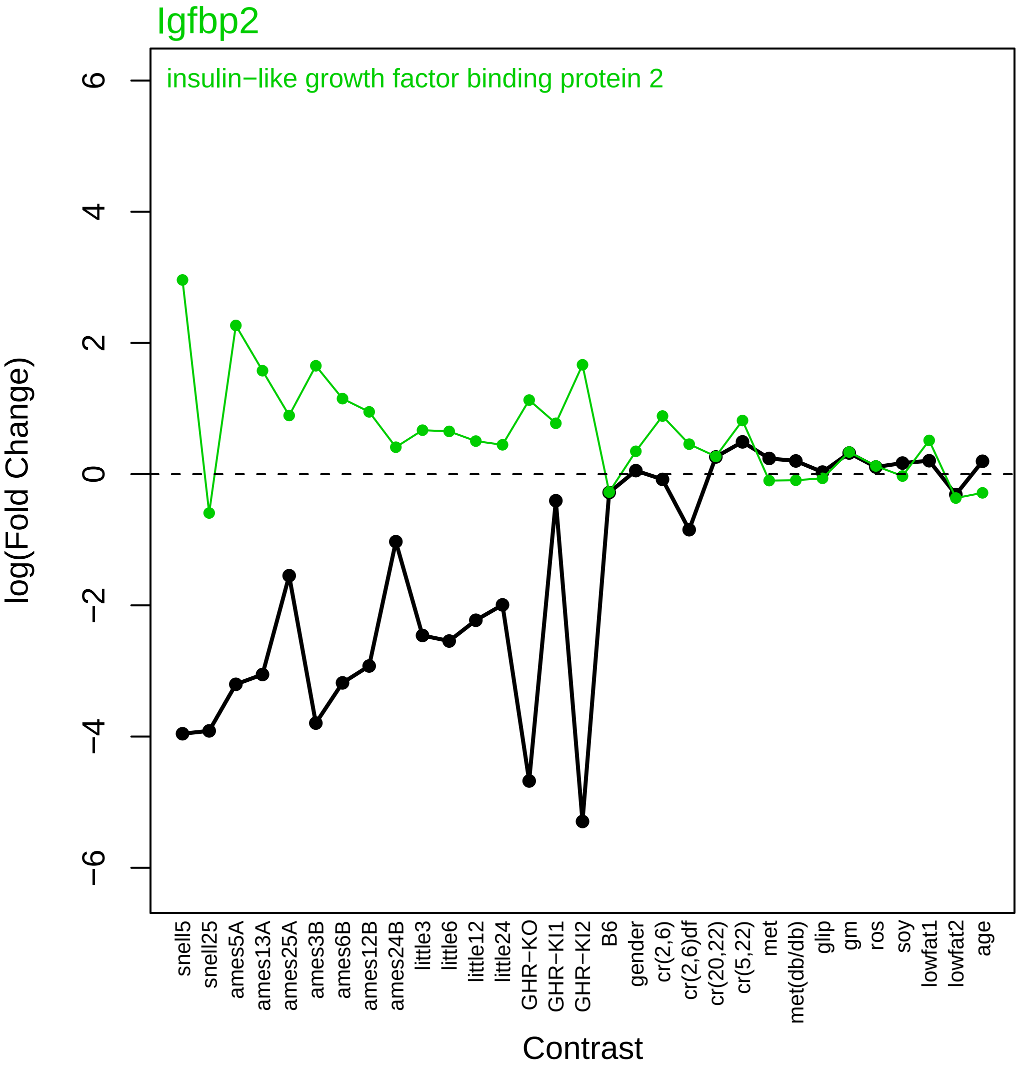

Igfbp2
insulin−like growth factor binding protein 2
log(Fold Change)
Contrast
6
4
2
0
−2
−4
−6
snell5
snell25
ames5A
ames13A
ames25A
ames3B
ames6B
ames12B
ames24B
little3
little6
little12
little24
GHR−KO
GHR−KI1
GHR−KI2
B6
gender
cr(2,6)
cr(2,6)df
cr(20,22)
cr(5,22)
met
met(db/db)
glip
gm
ros
soy
lowfat1
lowfat2
age

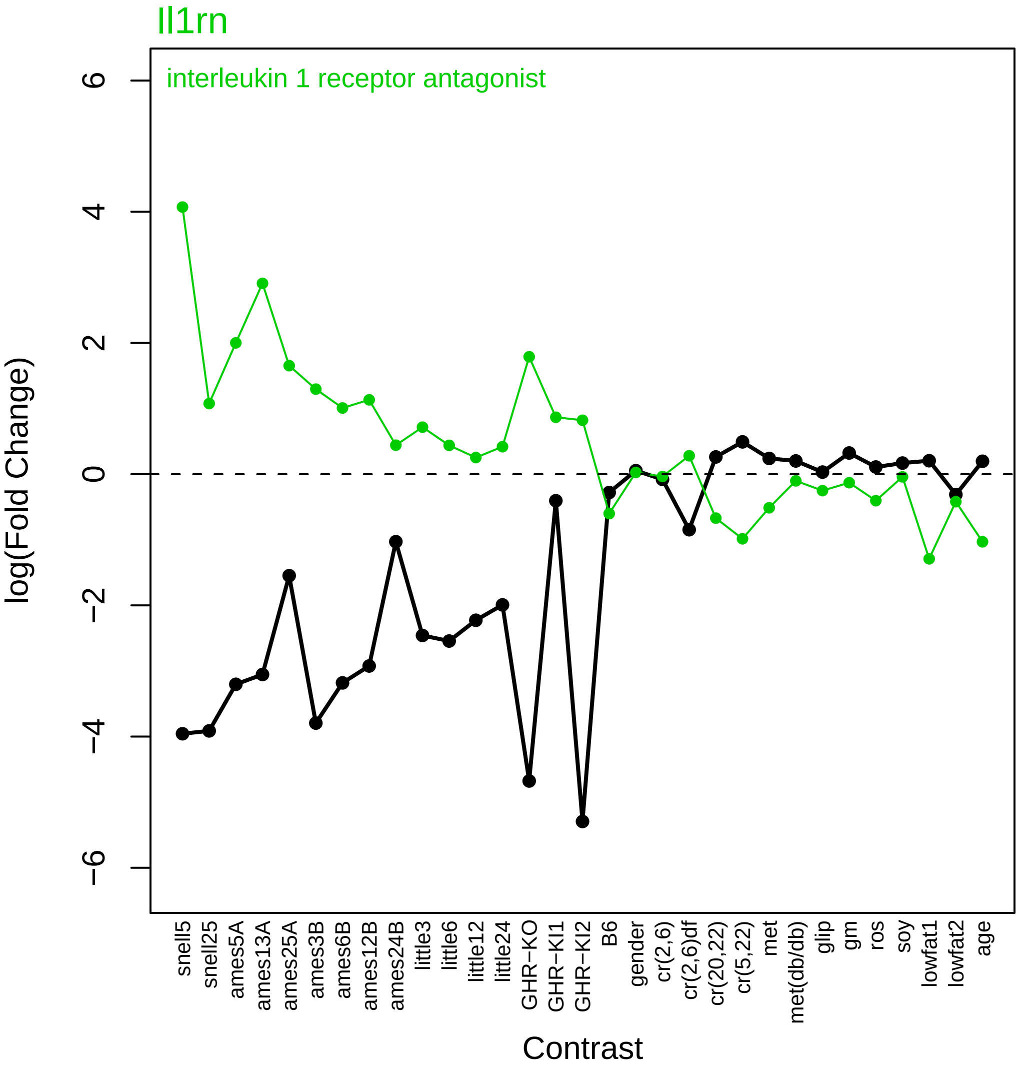

Il1rn
interleukin 1 receptor antagonist
log(Fold Change)
6
4
2
0
−2
−4
−6
snell5
snell25
ames5A
ames13A
ames25A
ames3B
ames6B
ames12B
ames24B
little3
little6
little12
little24
GHR−KO
GHR−KI1
GHR−KI2
B6
gender
cr(2,6)
cr(2,6)df
cr(20,22)
cr(5,22)
met
met(db/db)
glip
gm
ros
soy
lowfat1
lowfat2
age
Contrast

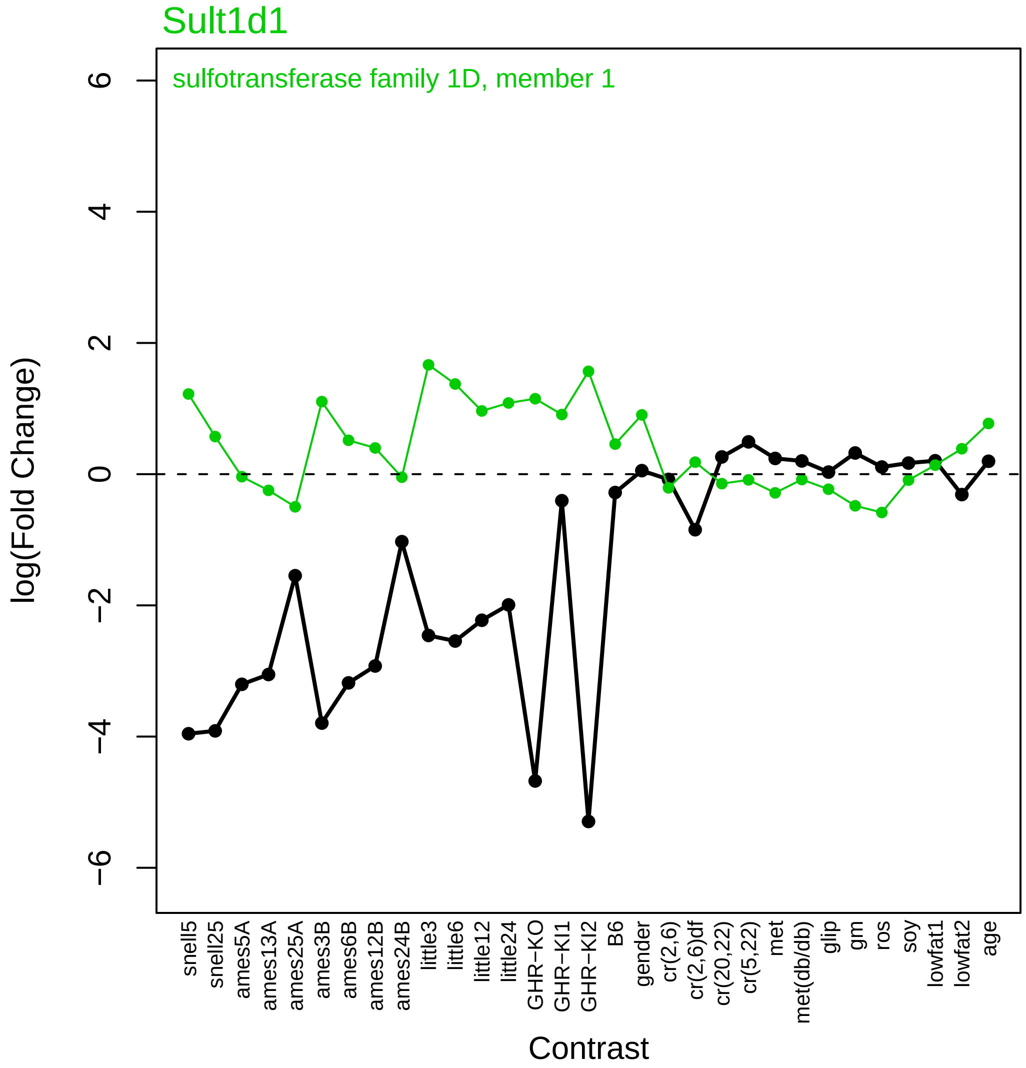
Sult1d1
sulfotransferase family 1D, member 1
log(Fold Change)
6
4
2
0
−2
−4
−6
snell5
snell25
ames5A
ames13A
ames25A
ames3B
ames6B
ames12B
ames24B
little3
little6
little12
little24
GHR−KO
GHR−KI1
GHR−KI2
B6
gender
cr(2,6)
cr(2,6)df
cr(20,22)
cr(5,22)
met
met(db/db)
glip
gm
ros
soy
lowfat1
lowfat2
age
Contrast

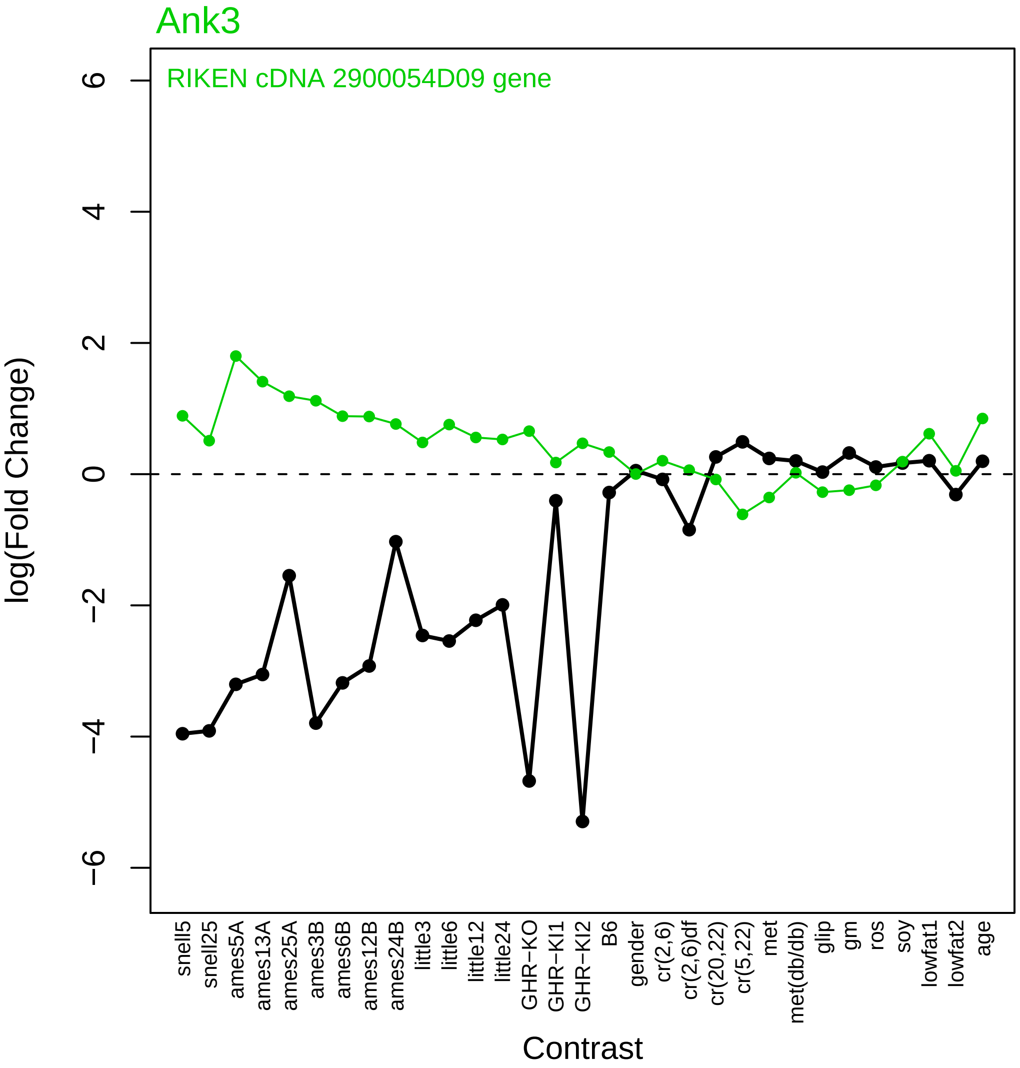
Ank3
RIKEN cDNA 2900054D09 gene
log(Fold Change)
6
4
2
0
−2
−4
−6
snell5
snell25
ames5A
ames13A
ames25A
ames3B
ames6B
ames12B
ames24B
little3
little6
little12
little24
GHR−KO
GHR−KI1
GHR−KI2
B6
gender
cr(2,6)
cr(2,6)df
cr(20,22)
cr(5,22)
met
met(db/db)
glip
gm
ros
soy
lowfat1
lowfat2
age
Contrast

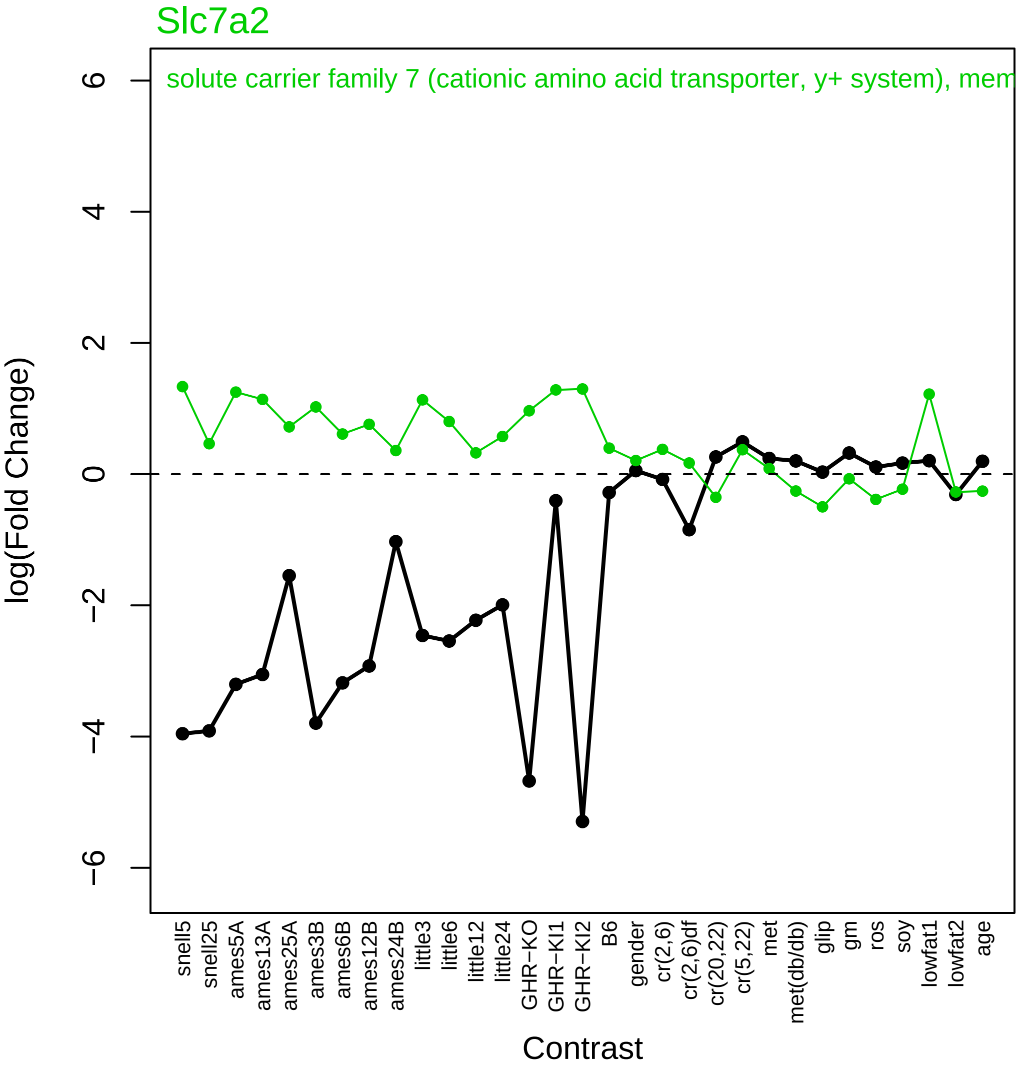
Slc7a2
solute carrier family 7 (cationic amino acid transporter, y+ system), mem
log(Fold Change)
6
4
2
0
−2
−4
−6
snell5
snell25
ames5A
ames13A
ames25A
ames3B
ames6B
ames12B
ames24B
little3
little6
little12
little24
GHR−KO
GHR−KI1
GHR−KI2
B6
gender
cr(2,6)
cr(2,6)df
cr(20,22)
cr(5,22)
met
met(db/db)
glip
gm
ros
soy
lowfat1
lowfat2
age
Contrast

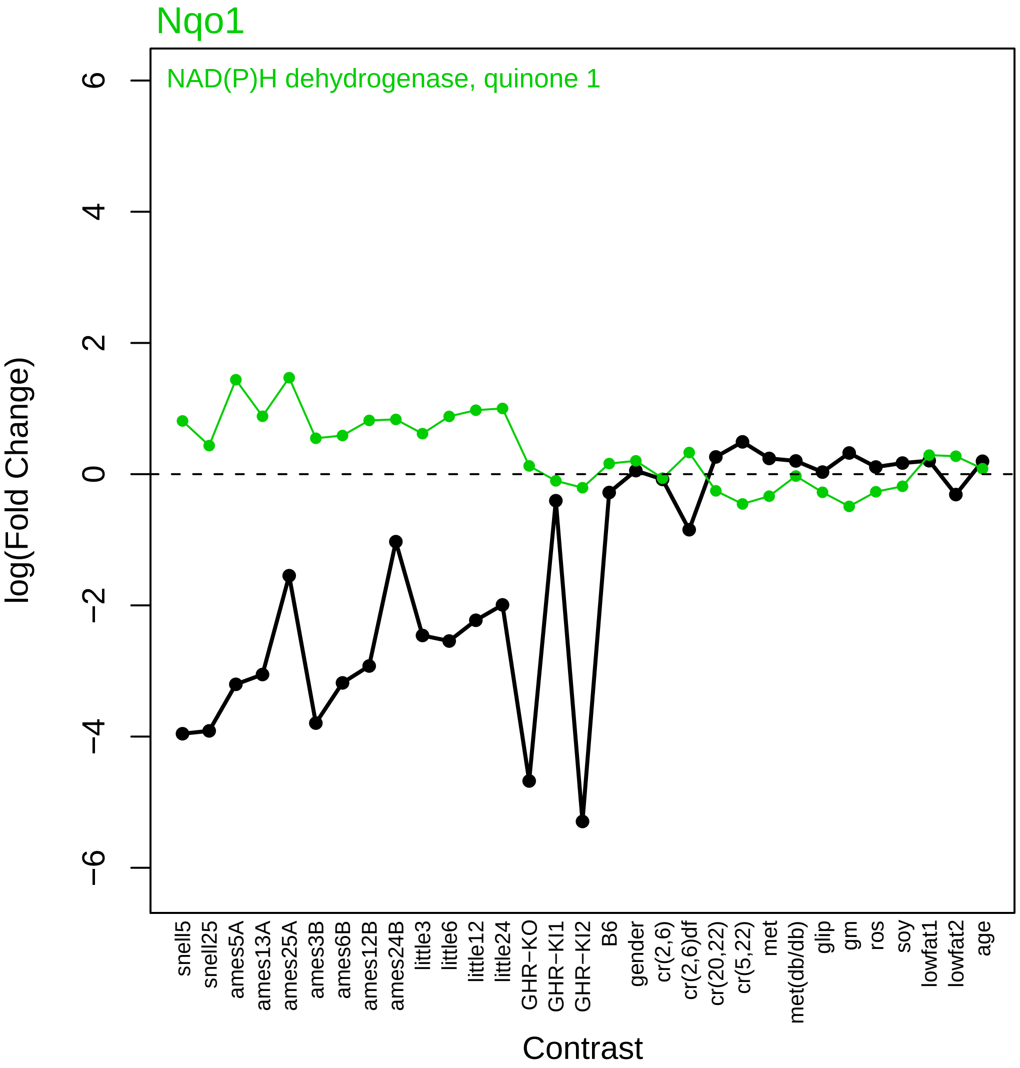
Nqo1
NAD(P)H dehydrogenase, quinone 1
log(Fold Change)
6
4
2
0
−2
−4
−6
snell5
snell25
ames5A
ames13A
ames25A
ames3B
ames6B
ames12B
ames24B
little3
little6
little12
little24
GHR−KO
GHR−KI1
GHR−KI2
B6
gender
cr(2,6)
cr(2,6)df
cr(20,22)
cr(5,22)
met
met(db/db)
glip
gm
ros
soy
lowfat1
lowfat2
age
Contrast

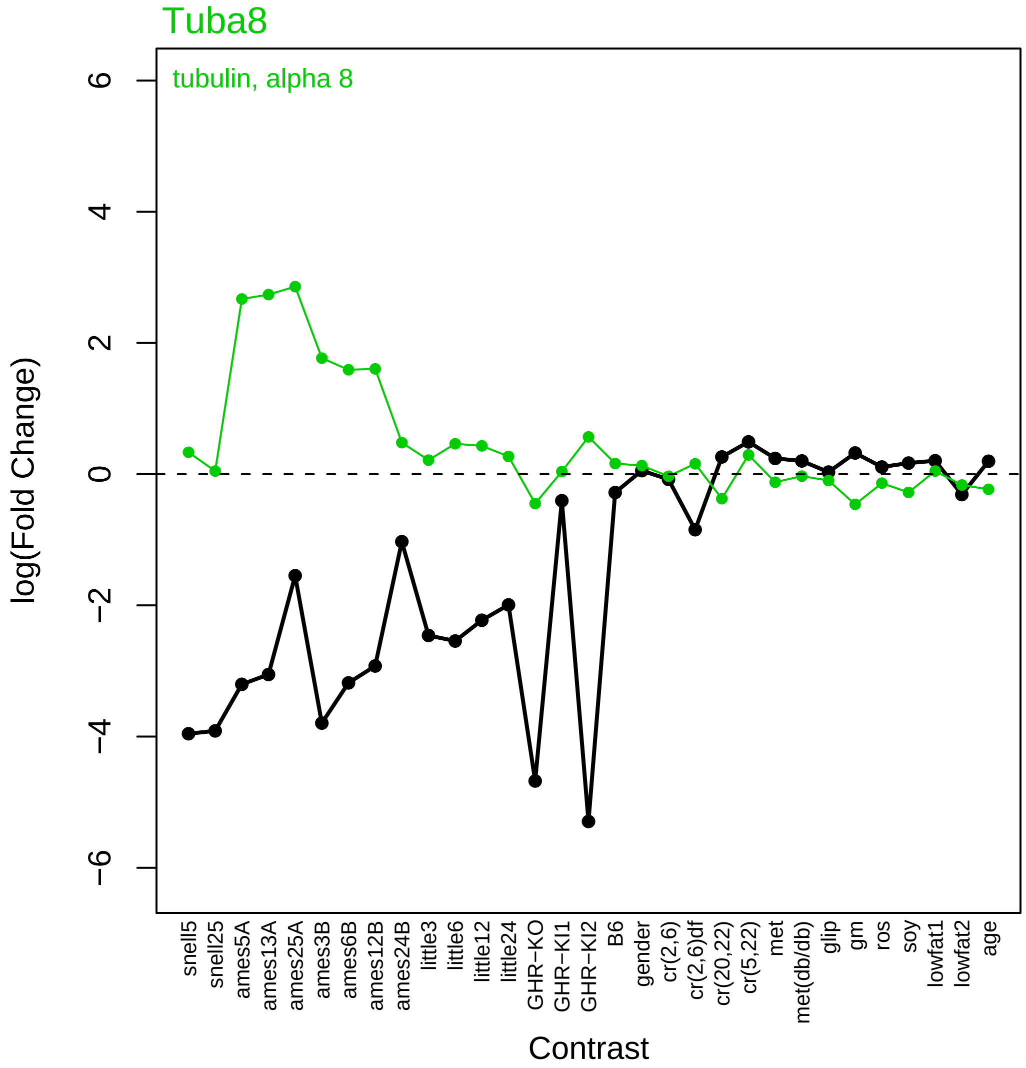

Tuba8
tubulin, alpha 8
log(Fold Change)
6
4
2
0
−2
−4
−6
snell5
snell25
ames5A
ames13A
ames25A
ames3B
ames6B
ames12B
ames24B
little3
little6
little12
little24
GHR−KO
GHR−KI1
GHR−KI2
B6
gender
cr(2,6)
cr(2,6)df
cr(20,22)
cr(5,22)
met
met(db/db)
glip
gm
ros
soy
lowfat1
lowfat2
age
Contrast

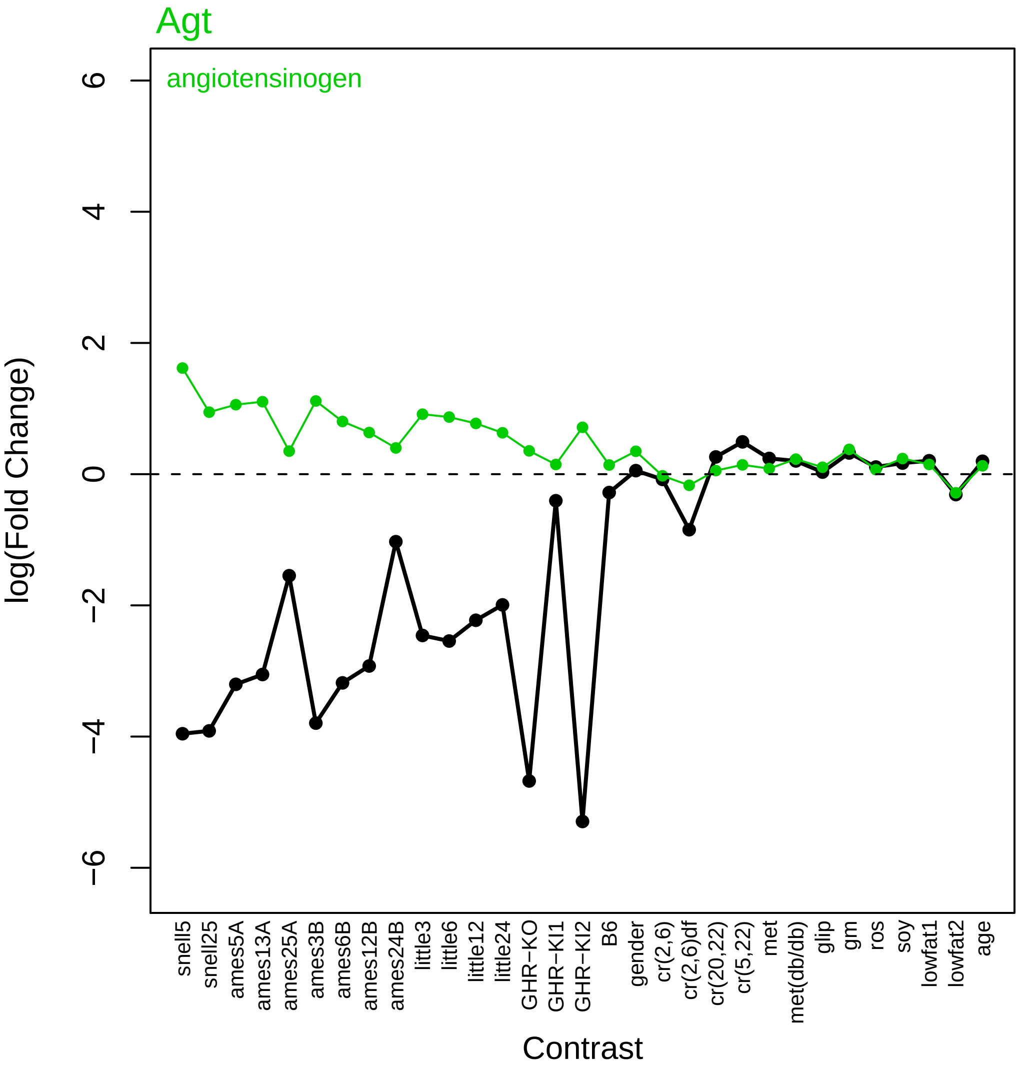
Agt
angiotensinogen
log(Fold Change)
6
4
2
0
−2
−4
−6
Contrast
snell5
snell25
ames5A
ames13A
ames25A
ames3B
ames6B
ames12B
ames24B
little3
little6
little12
little24
GHR−KO
GHR−KI1
GHR−KI2
B6
gender
cr(2,6)
cr(2,6)df
cr(20,22)
cr(5,22)
met
met(db/db)
glip
gm
ros
soy
lowfat1
lowfat2
age

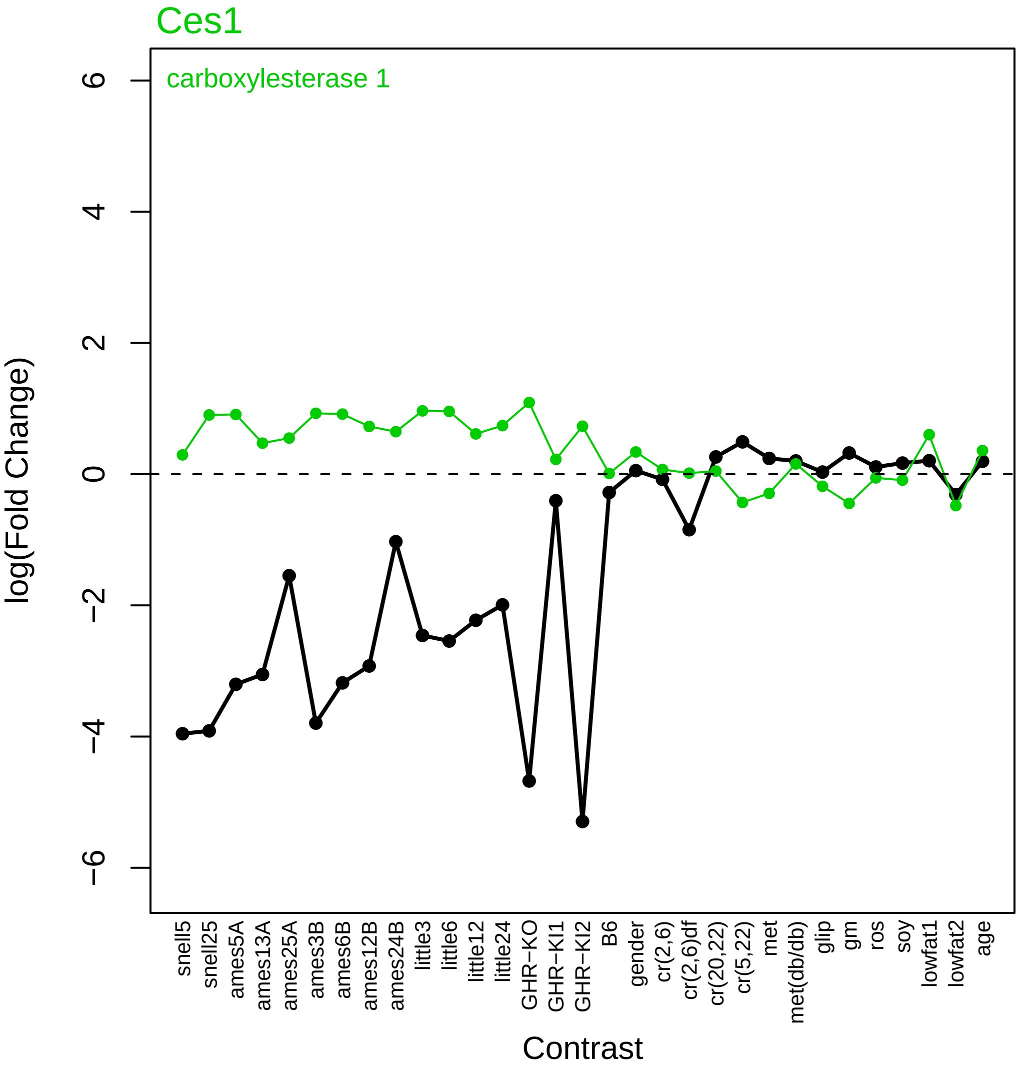

Ces1
carboxylesterase 1
log(Fold Change)
6
4
2
0
−2
−4
−6
Contrast
snell5
snell25
ames5A
ames13A
ames25A
ames3B
ames6B
ames12B
ames24B
little3
little6
little12
little24
GHR−KO
GHR−KI1
GHR−KI2
B6
gender
cr(2,6)
cr(2,6)df
cr(20,22)
cr(5,22)
met
met(db/db)
glip
gm
ros
soy
lowfat1
lowfat2
age

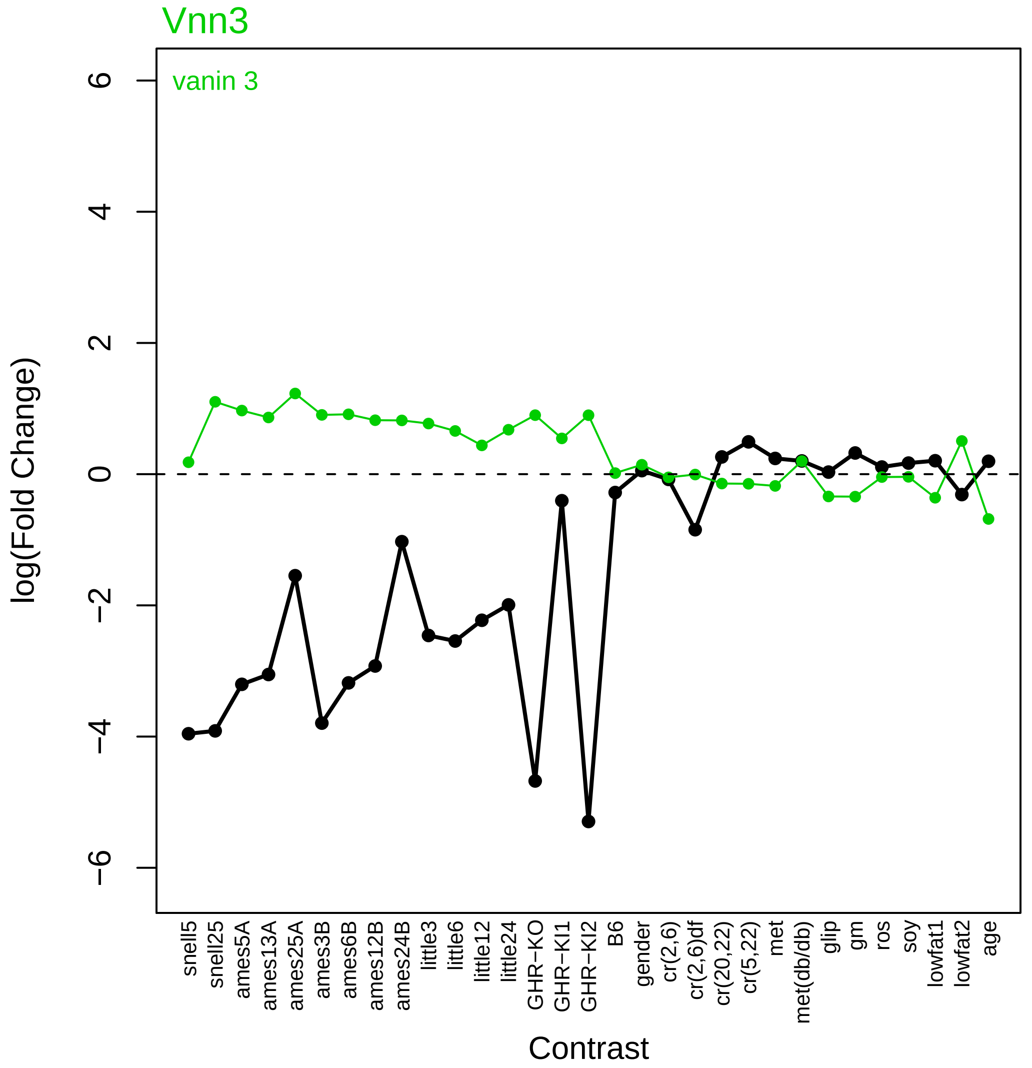

Vnn3
vanin 3
log(Fold Change)
6
4
2
0
−2
−4
−6
snell5
snell25
ames5A
ames13A
ames25A
ames3B
ames6B
ames12B
ames24B
little3
little6
little12
little24
GHR−KO
GHR−KI1
GHR−KI2
B6
gender
cr(2,6)
cr(2,6)df
cr(20,22)
cr(5,22)
met
met(db/db)
glip
gm
ros
soy
lowfat1
lowfat2
age
Contrast

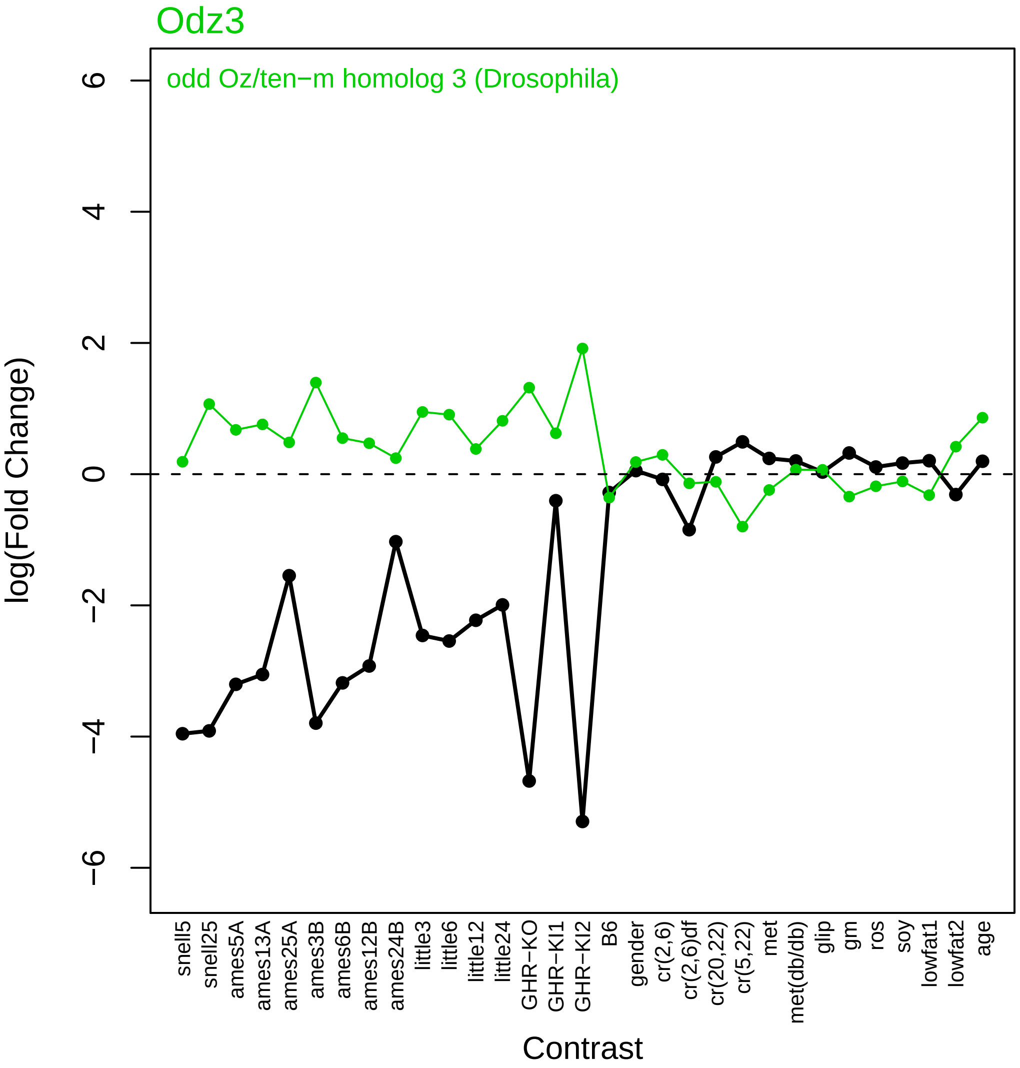
Odz3
odd Oz/ten−m homolog 3 (Drosophila)
log(Fold Change)
6
4
2
0
−2
−4
−6
snell5
snell25
ames5A
ames13A
ames25A
ames3B
ames6B
ames12B
ames24B
little3
little6
little12
little24
GHR−KO
GHR−KI1
GHR−KI2
B6
gender
cr(2,6)
cr(2,6)df
cr(20,22)
cr(5,22)
met
met(db/db)
glip
gm
ros
soy
lowfat1
lowfat2
age
Contrast
